# Supplementary figures and images for: Receptor tyrosine kinase inhibition leads to regression of acral melanoma by targeting the tumor microenvironment
Source: J Exp Clin Cancer Res. 2024 Dec 3;43:317. doi: 10.1186/s13046-024-03234-1 (PMC11613472; doi:10.1186/s13046-024-03234-1)

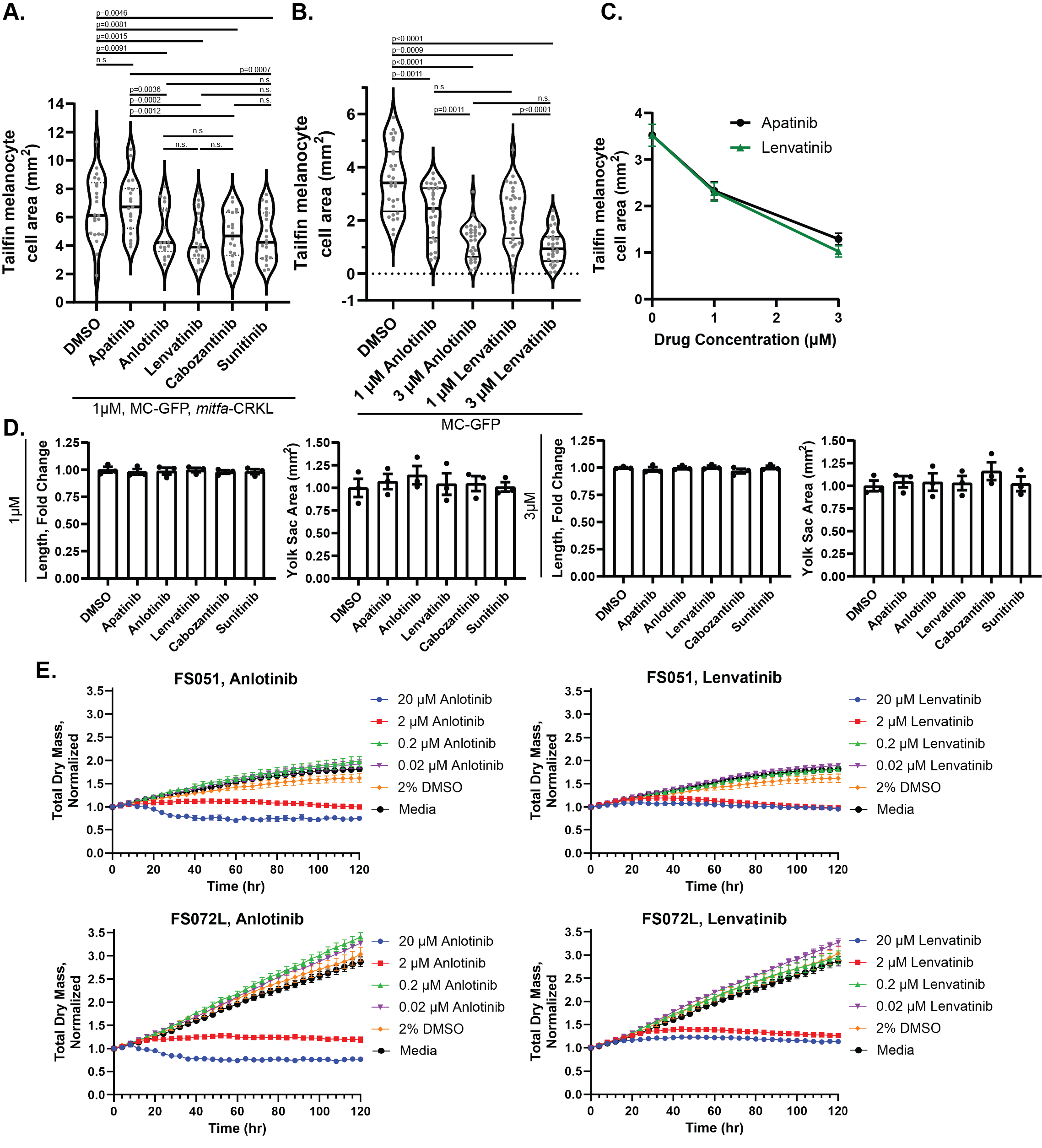

Supplement: Supplementary file 1 — Supplementary Material 1. Figure S1: Multi-RTK inhibitors prevent melanogenesis in wild-type and mitfa-CRKL premalignant zebrafish in a dose-dependent manner without organism-level toxicity. (A) Tailfin melanocyte cell area at 1µM dose of multi-RTK inhibitors in the mitfa-CRKL MC-GFP model. (B-C) Tailfin melanocyte cell area with treatment of Anlotinib and Lenvatinib at 1 and 3µM in CRKL wild-type MC-GFP cells. (D) Organism-level toxicity evaluation by length and yolk-sac area for each multi-RTK inhibitor in mitfa-CRKL MC-GFP zebrafish.(E) Dark (FS051) and lightly pigmented (FS072L) primary human melanocytes were treated with Anlotinib and Lenvatinib at different doses and imaged for five days with quantitative phase imaging on a Livecyte instrument. The average total dry mass of three replicate experiments are plotted. [file 13046_2024_3234_MOESM1_ESM.png]

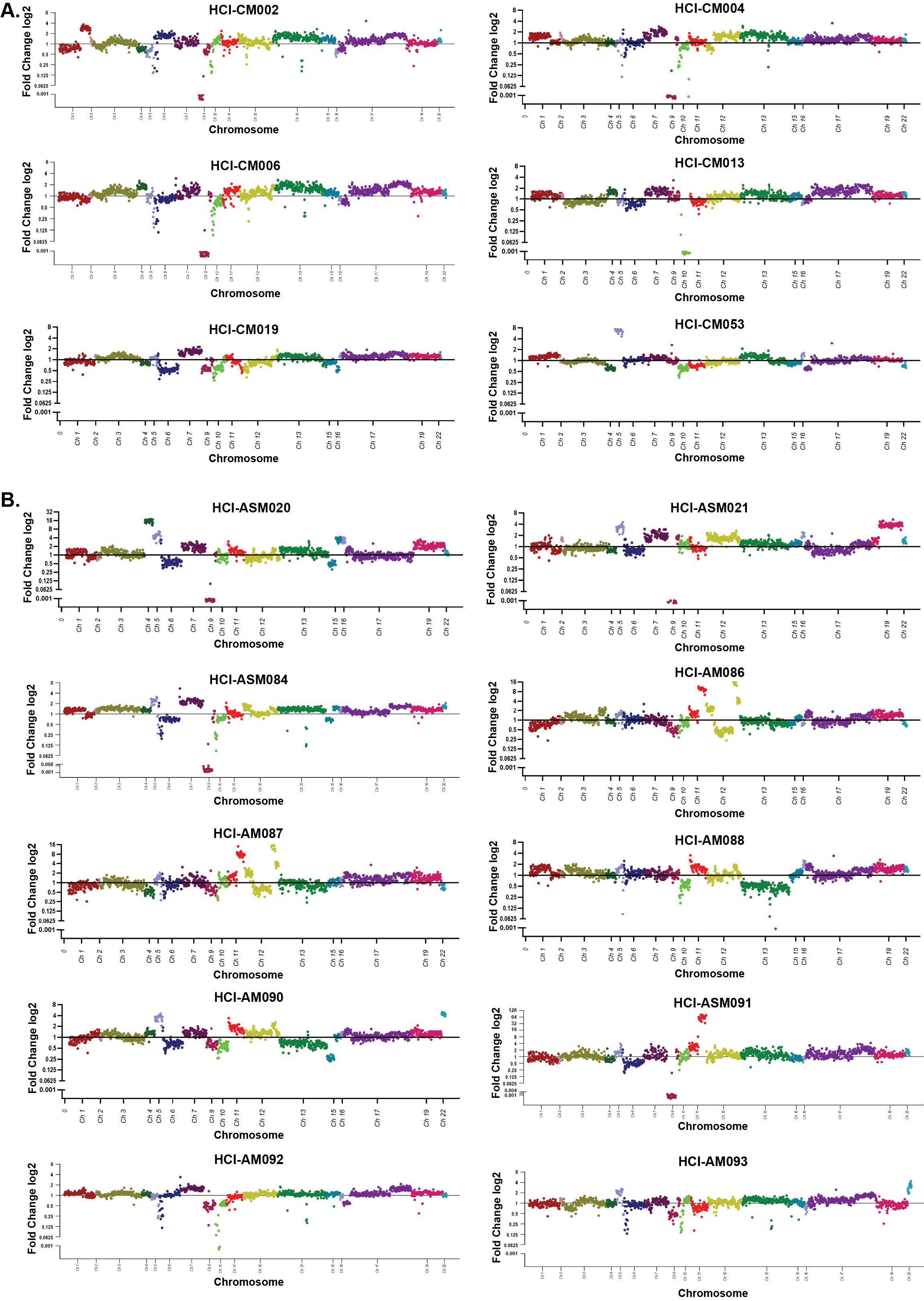

Supplement: Supplementary file 2 — Supplementary Material 2. Figure S2: Raindrop plots representing CNV for each PDX model. (A) CM CNV plots and (B) AM CNV plots. Paired PDX models developed from the same patient include HCI-CM004 and HCI-CM019, HCI-ASM020 and HCI-ASM021, and HCI-AM086 and HCI-AM087. [file 13046_2024_3234_MOESM2_ESM.png]

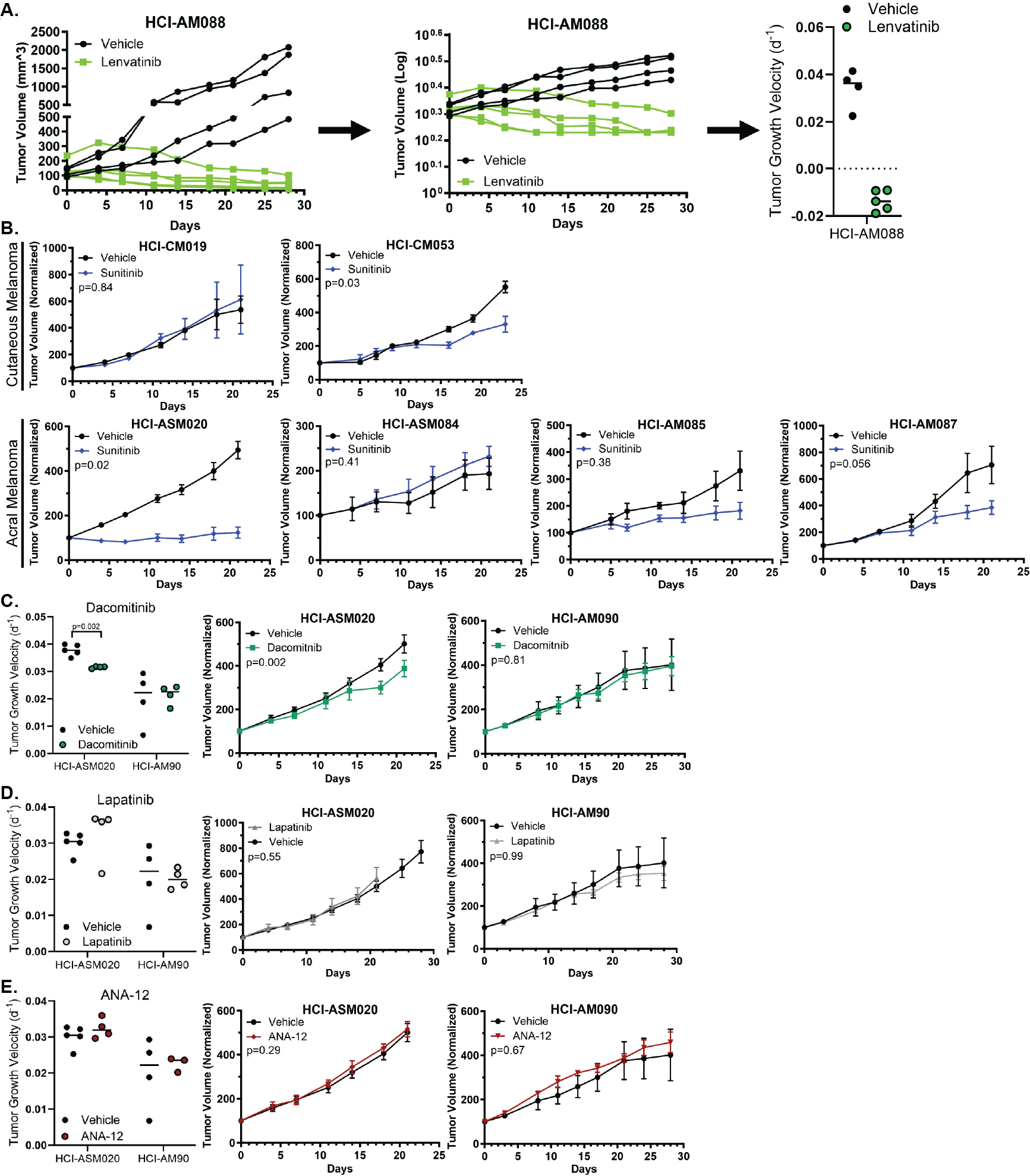

Supplement: Supplementary file 3 — Supplementary Material 3. Figure S3: Other kinase inhibitor responses in PDX tumor models. (A) An example of the transformations for creating tumor growth velocity and HCI-AM088 is shown per Hather 2014 [ 49 ]. In the left panel, individual tumor sizes are shown in the HCI-AM088 Lenvatinib and vehicle cohorts. These values undergo Log10 transformation using an absolute minimum tumor size of 50mm3 to avoid exponential data skewing from small tumor volumes (middle panel). In the right panel, the logarithmic slope of each tumor is plotted as a single dot in the tumor growth velocity plot. Negative average growth rates represent regression and positive growth rates indicate tumor growth. Near-zero average growth rates are oncostatic. (B) Average normalized tumor sizes are shown for each PDX model treated with Sunitinib. Tumor growth velocities and averaged normalized tumor sizes are shown for (C) Dacomitinib, (D) Lapatinib, and (E) ANA-12. All tested PDX models are listed for each drug. P-values are calculated with the student T-test per Hather 2014 [ 49 ]. [file 13046_2024_3234_MOESM3_ESM.png]

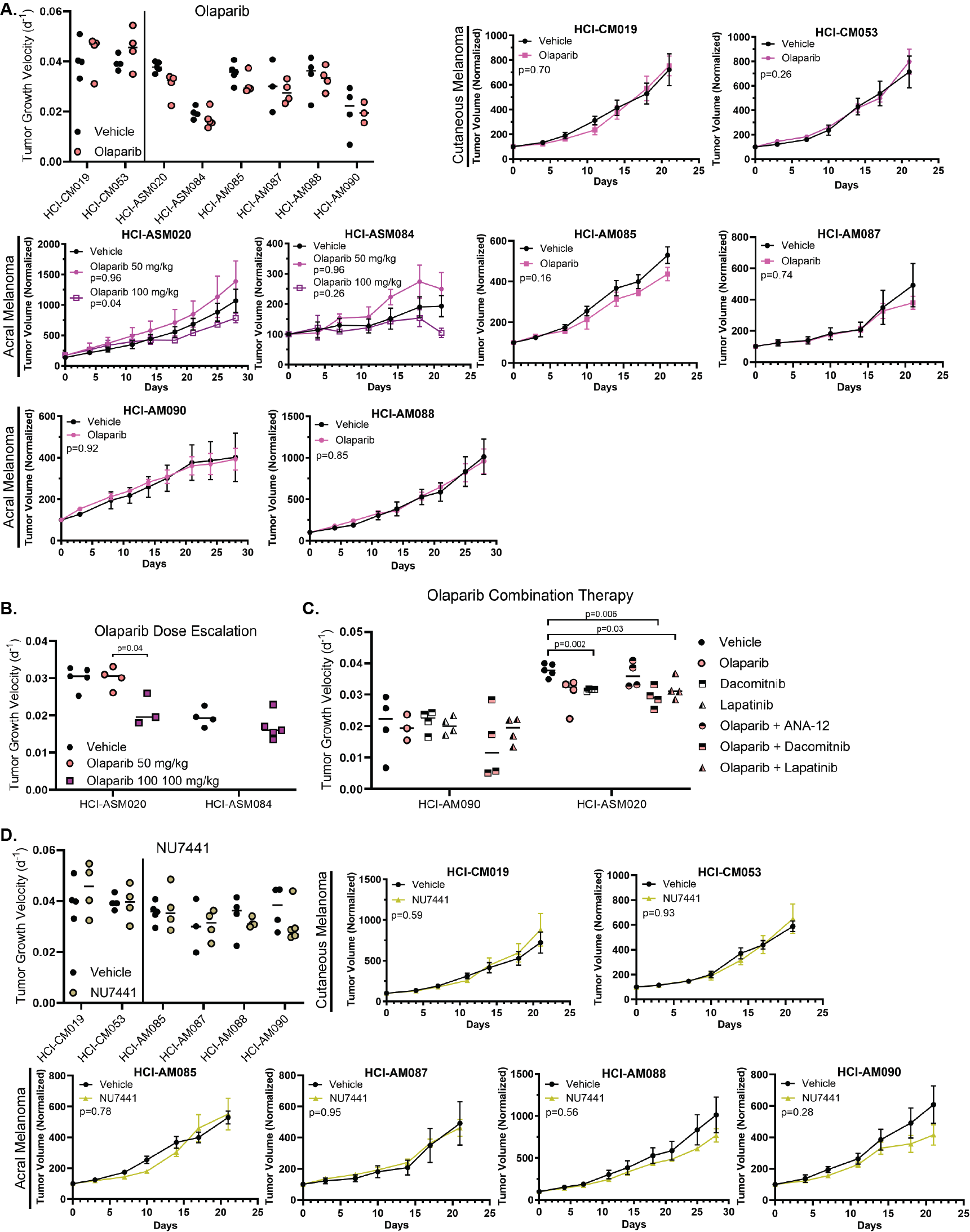

Supplement: Supplementary file 4 — Supplementary Material 4. Figure S4: DNA repair inhibitor responses in all tested PDX tumor models. (A) Tumor growth velocities and normalized tumor volume are shown for each PDX model treated with Olaparib. (B) Dose-escalation and (C) combination therapy with other agents were performed in select PDX models. (D) Growth velocity and normalized tumor volume are shown with NU7441 treatment. All tested PDX models are listed for each drug. P-values are calculated with the student T-test per Hather 2014 [ 49 ]. [file 13046_2024_3234_MOESM4_ESM.png]

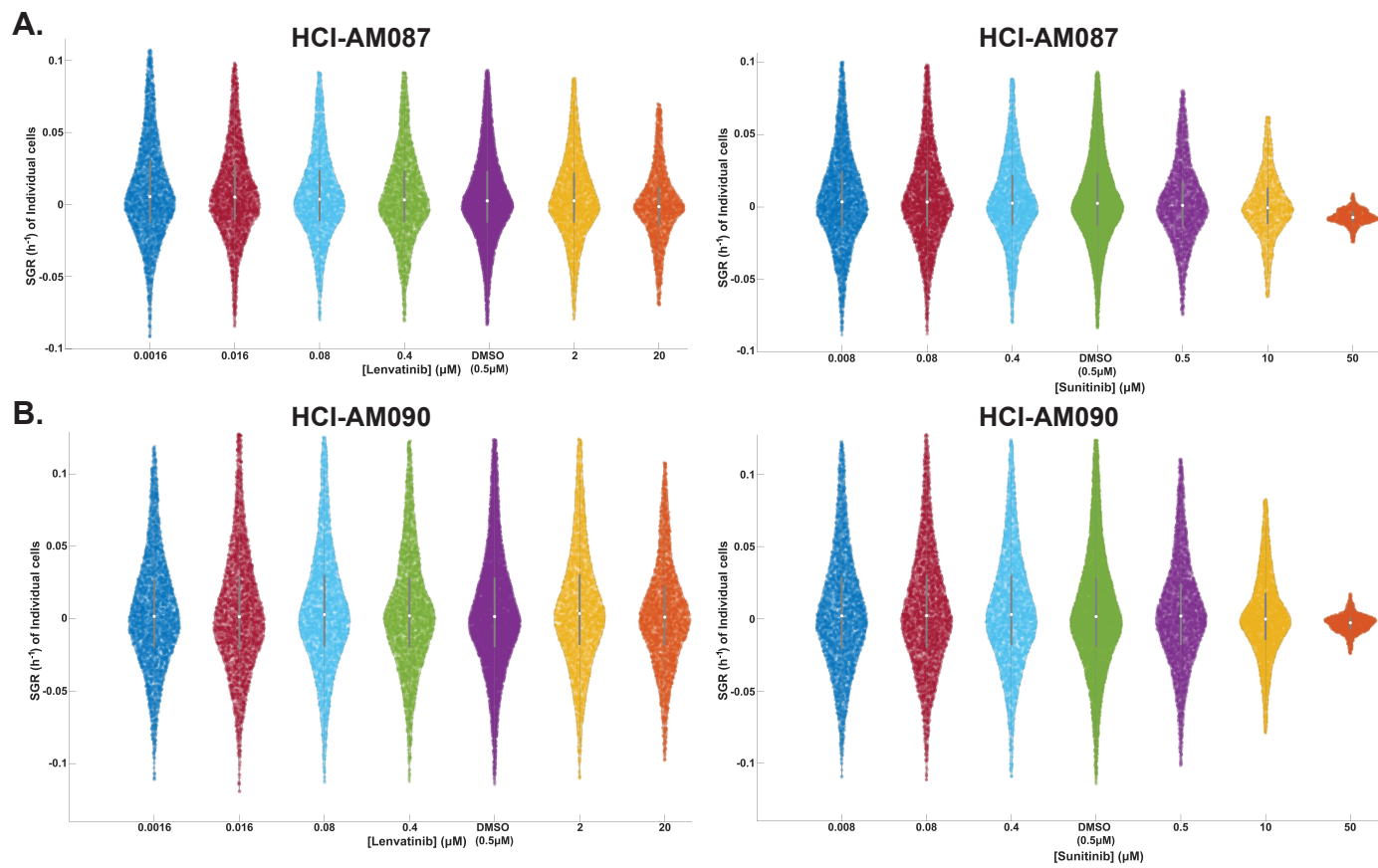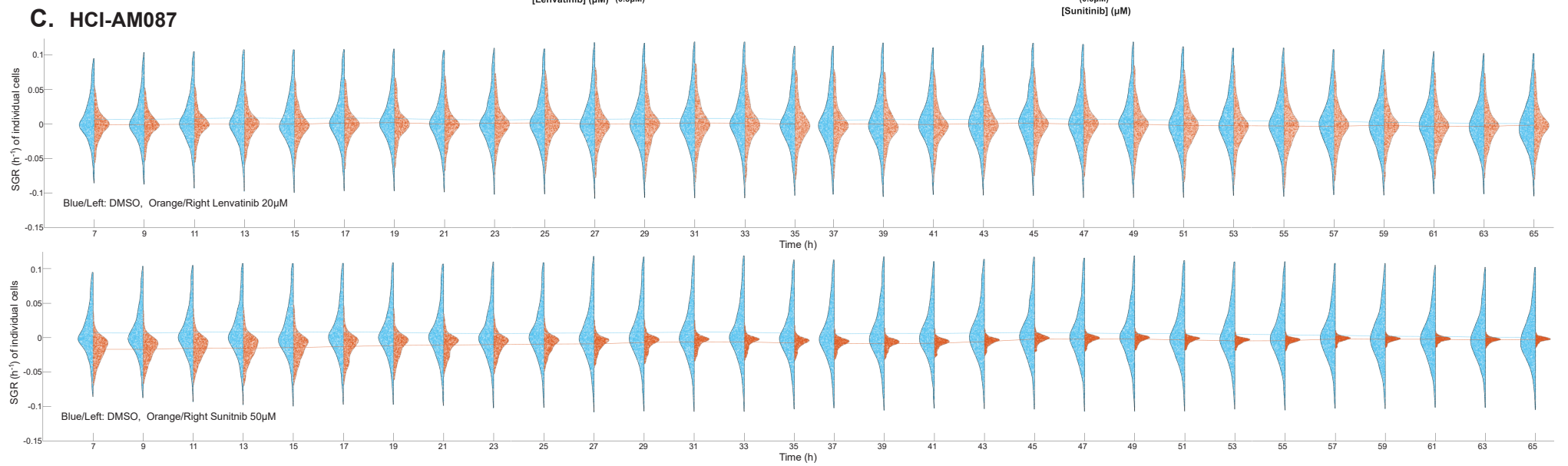

Supplement: Supplementary file 5 — Supplementary Material 5. Figure S5: QPI representative SGR population dynamics. Representative SGR violin plots of all analyzed (A) HCI-AM087 and (B) HCI-AM090 cells are shown at tested concentrations of Lenvatinib and Sunitinib. The white central dot represents the average, grey bars represent standard deviation, and individual colored dots represents individual cells. Higher concentrations of drugs often have fewer cells, leading to smaller violins. (C) Representative two-sided violin plots show differences in SGR for DMSO (blue, left) and either Lenvatinib or Sunitinib (orange, right). Colored lines represent mean of each population, x-axis represents time (h), and y-axis represent SGR (h-1). [file 13046_2024_3234_MOESM5_ESM.pdf]

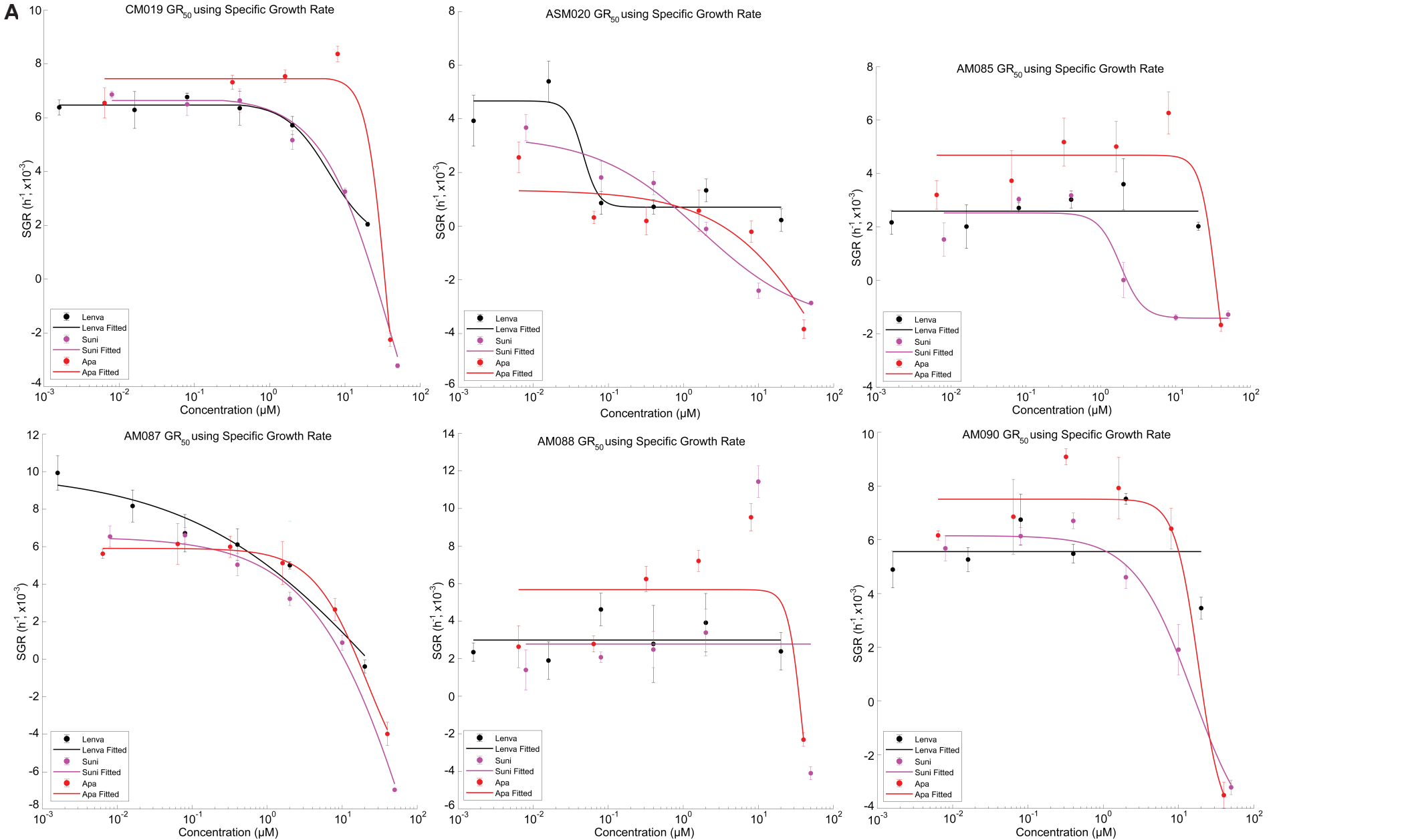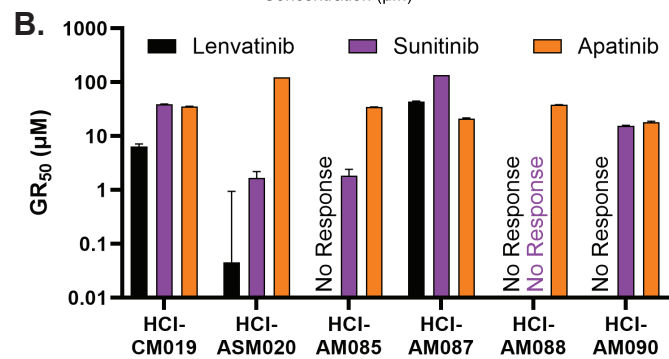

Supplement: Supplementary file 6 — Supplementary Material 6. Figure S6: QPI GR50 best-fit curves. (A) Best fit curves used to identify GR50 in the different PDX cell cultures. Horizontal lines indicate failure to calculate or extrapolate a GR50 value. (B) Comparison of GR50 in each cell line for Sunitinib and the dual FGFR/VEGFR inhibitors Lenvatinib and Apatanib. [file 13046_2024_3234_MOESM6_ESM.pdf]
