# Supplementary material for: Receptor tyrosine kinase inhibition leads to regression of acral melanoma by targeting the tumor microenvironment
Source: J Exp Clin Cancer Res. 2024 Dec 3;43:317. doi: 10.1186/s13046-024-03234-1 (PMC11613472; doi:10.1186/s13046-024-03234-1)
Supplement: Supplementary file 13 — Supplementary Material 13. Supplemental Data 6: PDX Model Data Sheets. [file 13046_2024_3234_MOESM13_ESM.pdf]

HCI-CM002, Cutaneous Melanoma

Primary Tumor Location	PDX Tumor Location	Other Known Metastatic Locations
Right Cheek Skin	Right Superficial Parotid Gland	Lung

Clinical Tumor Histology

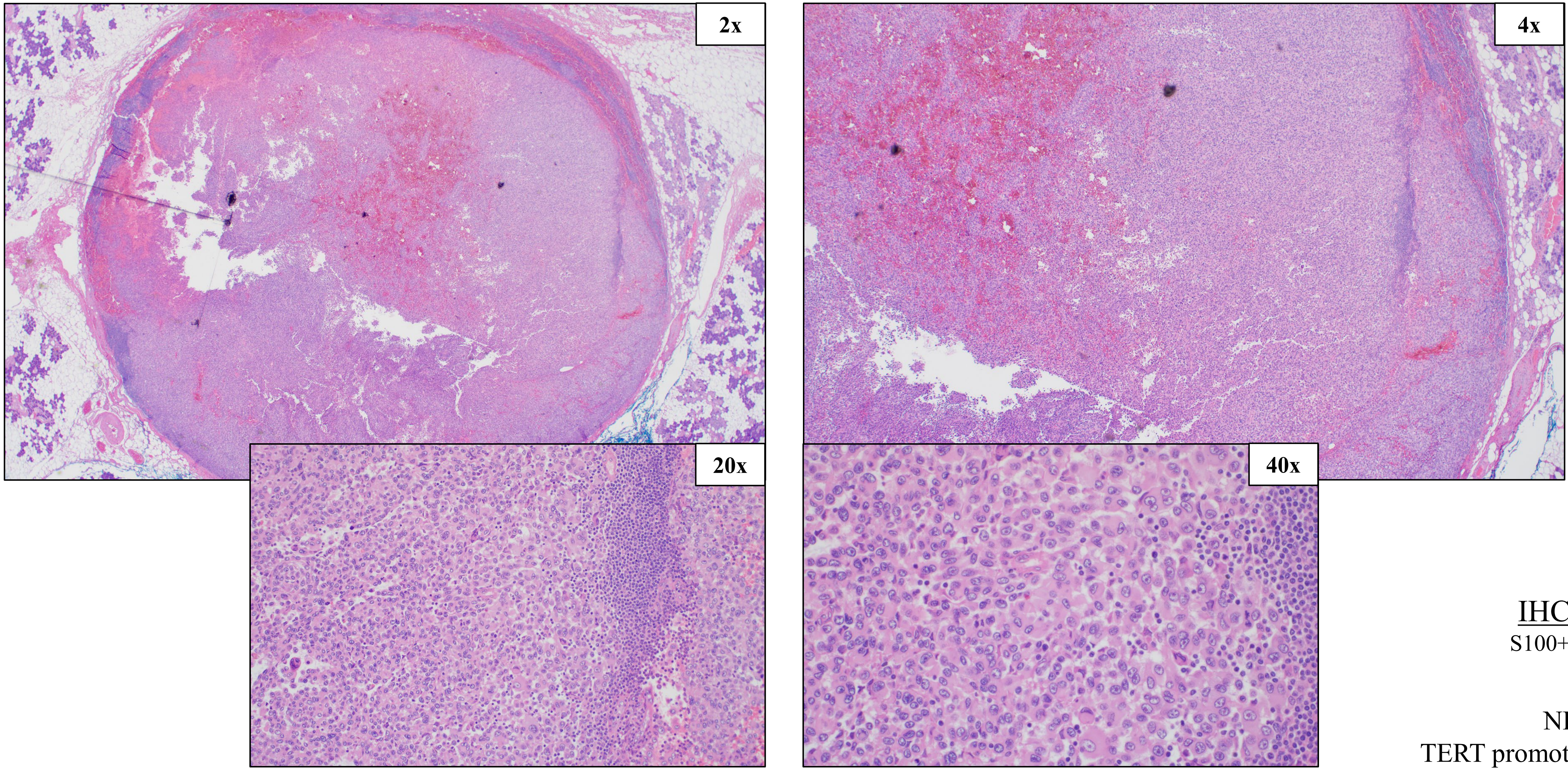

IHC Stains:
S100+, MelanA+

NRAS Q61R,
TERT promoter mutation

HCI-CM002, Cutaneous Melanoma

SNV (%VAF)

NRAS c.182A>G p.Q61R (87%)
TERT promoter, chr5:1295242GG>AA (94%)

CNV

Amplification: NRAS, BRCA1
Loss: PTEN, TERT, MC1R
Deep deletion: CDKN2A

PDX Tumor Histology, Passage 2

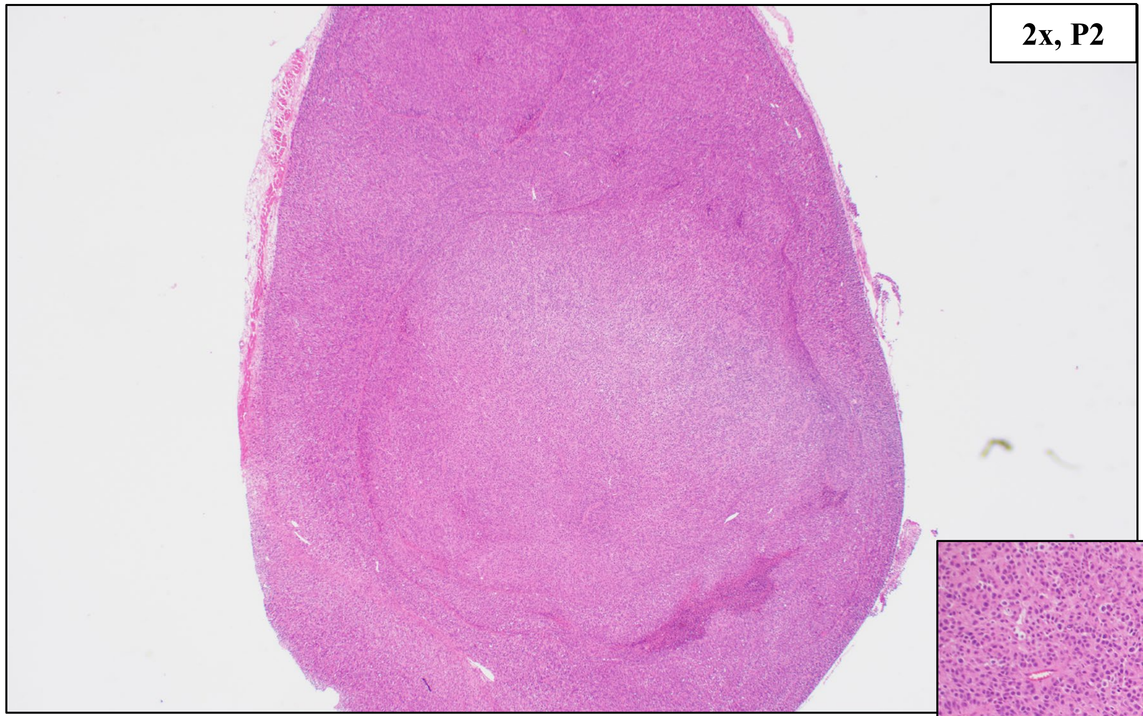

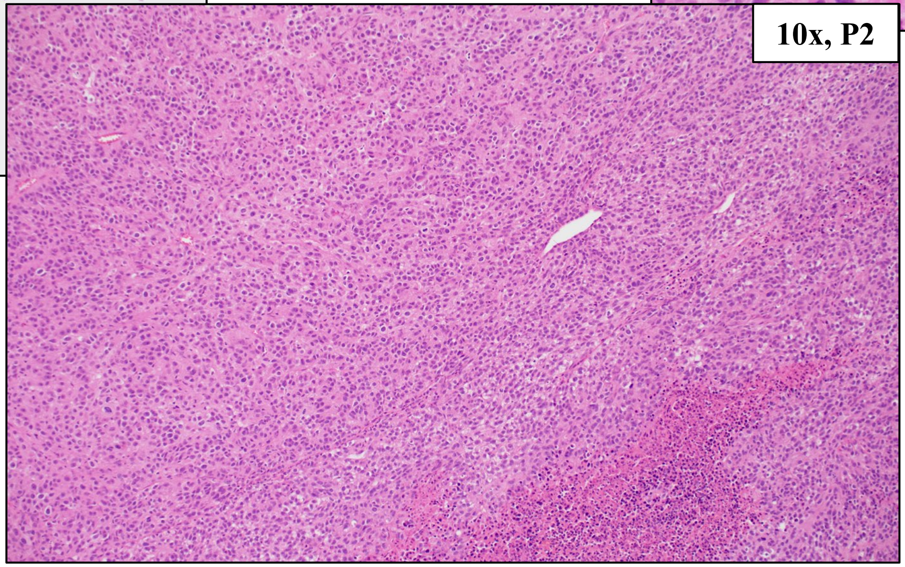

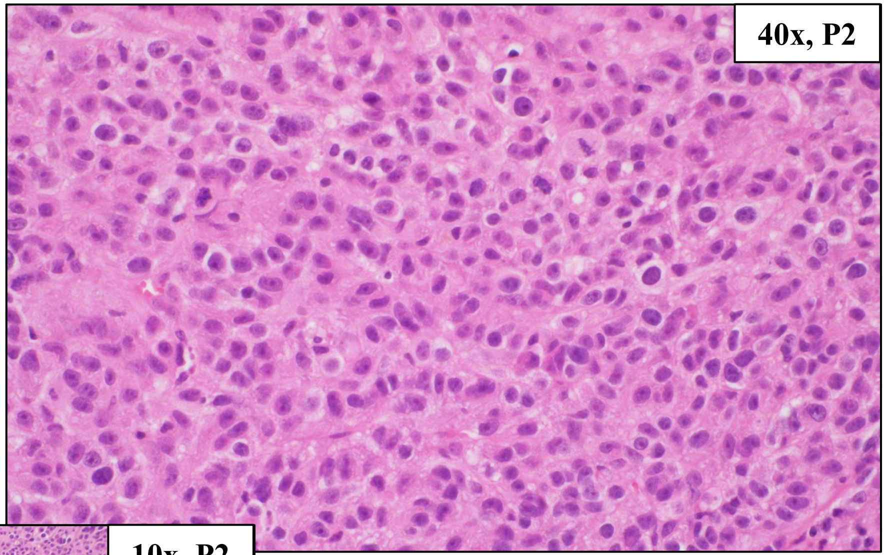

NRAS Q61R, TERT promoter mutation

HCI-CM004, Cutaneous Melanoma (from same patient as HCI-CM019)

Primary Tumor Location	PDX Tumor Location	Other Known Metastatic Locations
Scalp	Omentum	Brain, Bilateral Lungs, Back Skin, Supraclavicular Lymph Nodes, Brain

Clinical Tumor Histology

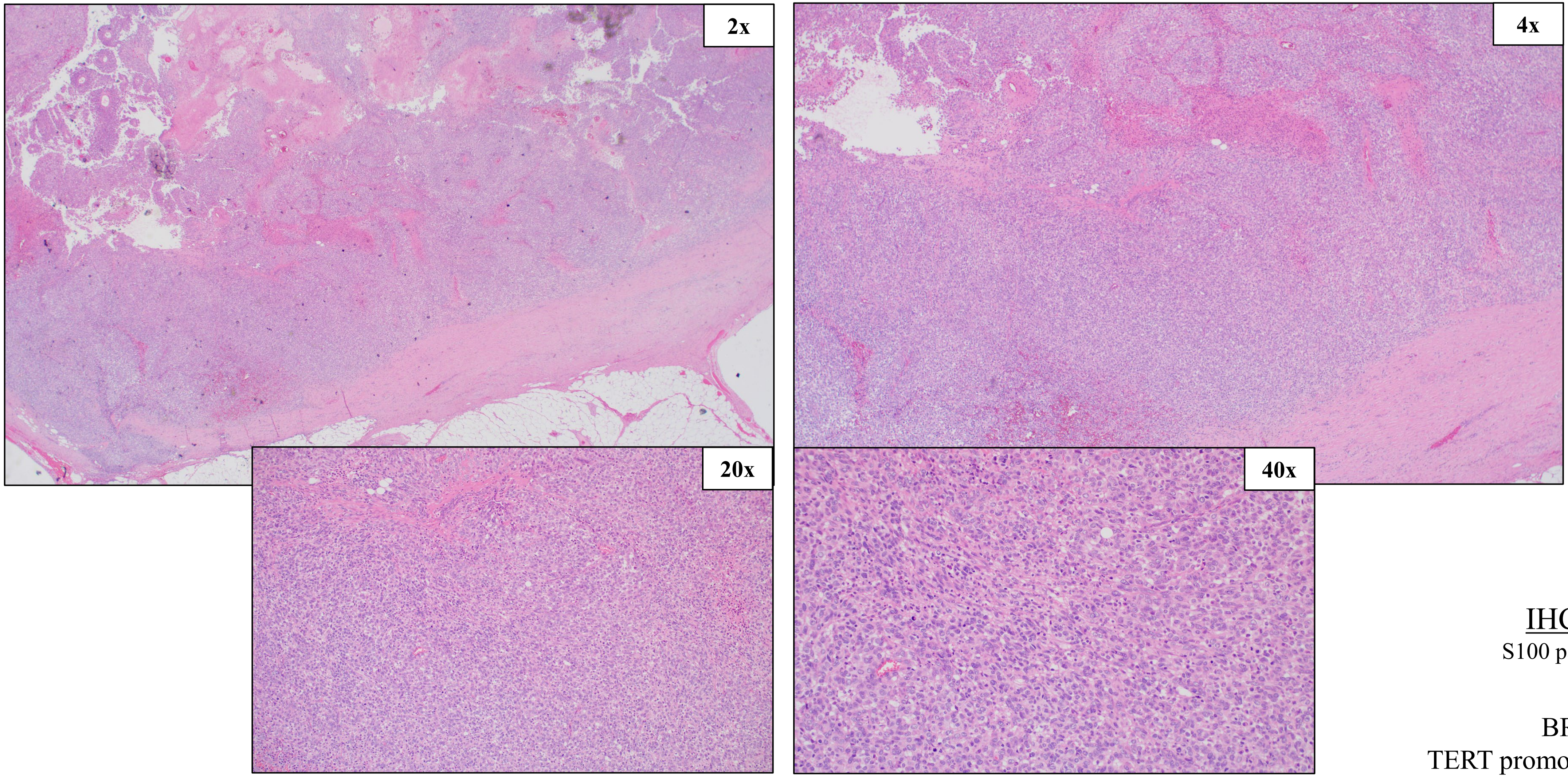

IHC Stains:
S100 partial positive

BRAF V600E,
TERT promoter mutation

HCI-CM004, Cutaneous Melanoma (from same patient as HCI-CM004)

<u>SNV (%VAF)</u>

BRAF c.1799T>A p.V600E (83%),
TERT chr5:1295250G>A (66%)

<u>CNV</u>

Amplification: MET, BRAF, BRCA2
Loss: PTEN
Deep deletion: CDKN2A

<u>PDX Tumor Histology, Passages 2 and 4</u>

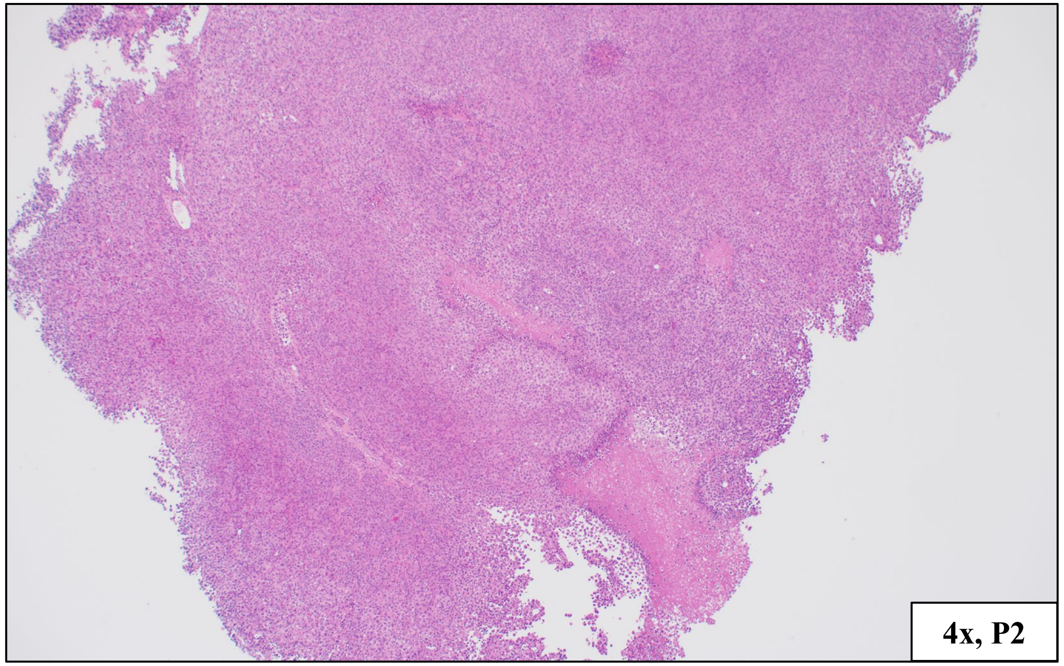

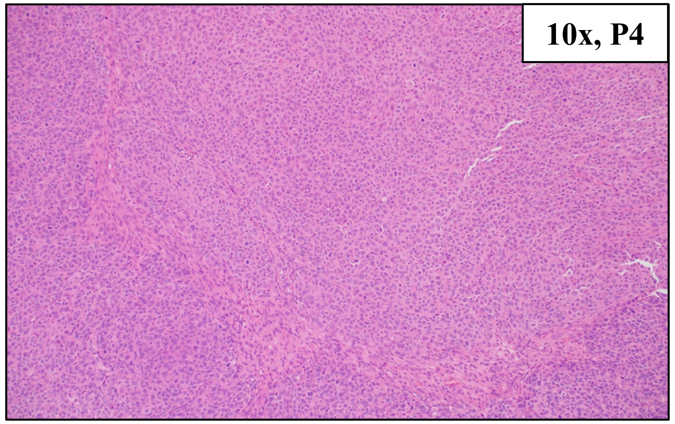

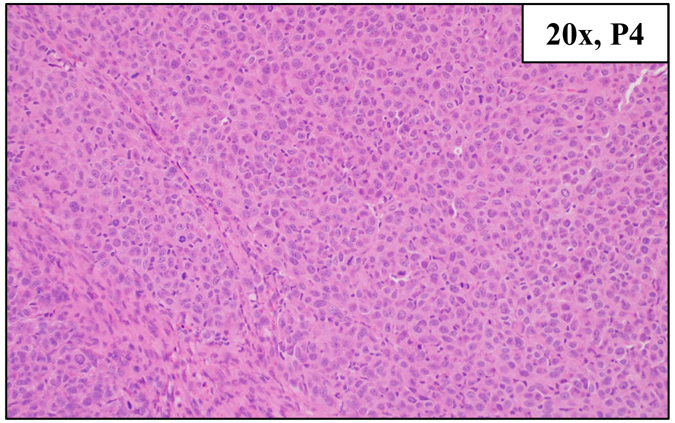

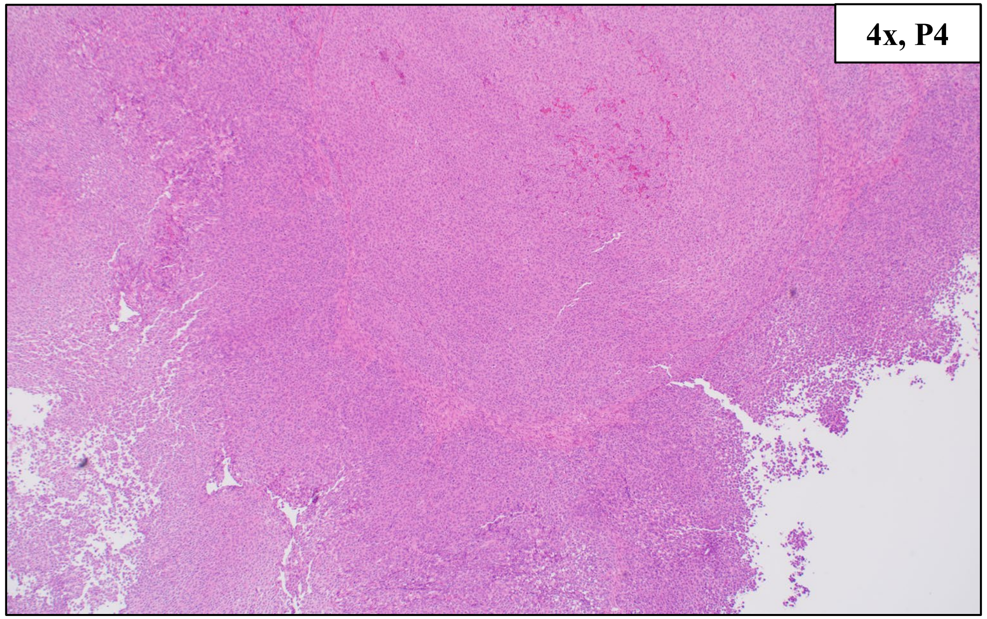

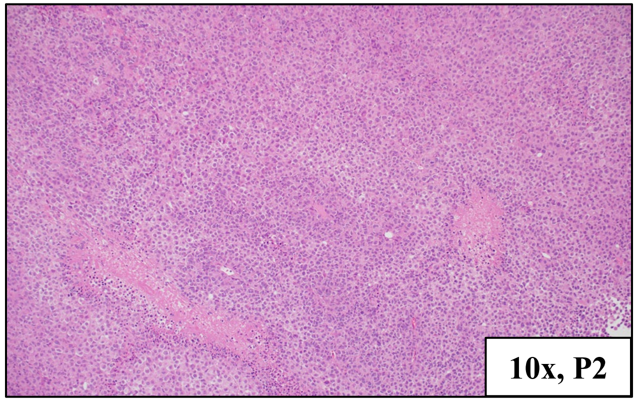

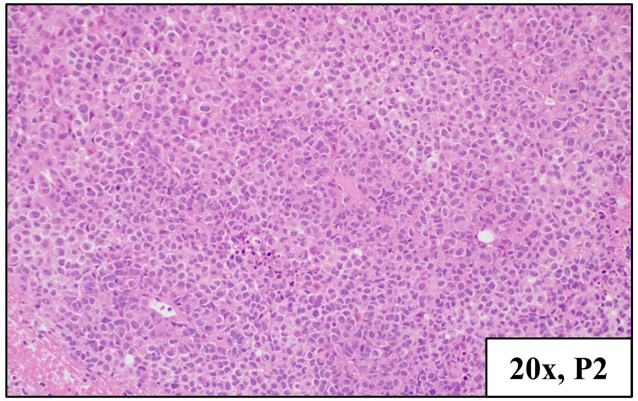

BRAF V600E, TERT promoter mutation

HCI-CM005, Cutaneous Melanoma

Primary Tumor Location	PDX Tumor Location	Other Known Metastatic Locations
Left Temporal Face Skin	Left Parotid	None

Clinical Tumor Histology

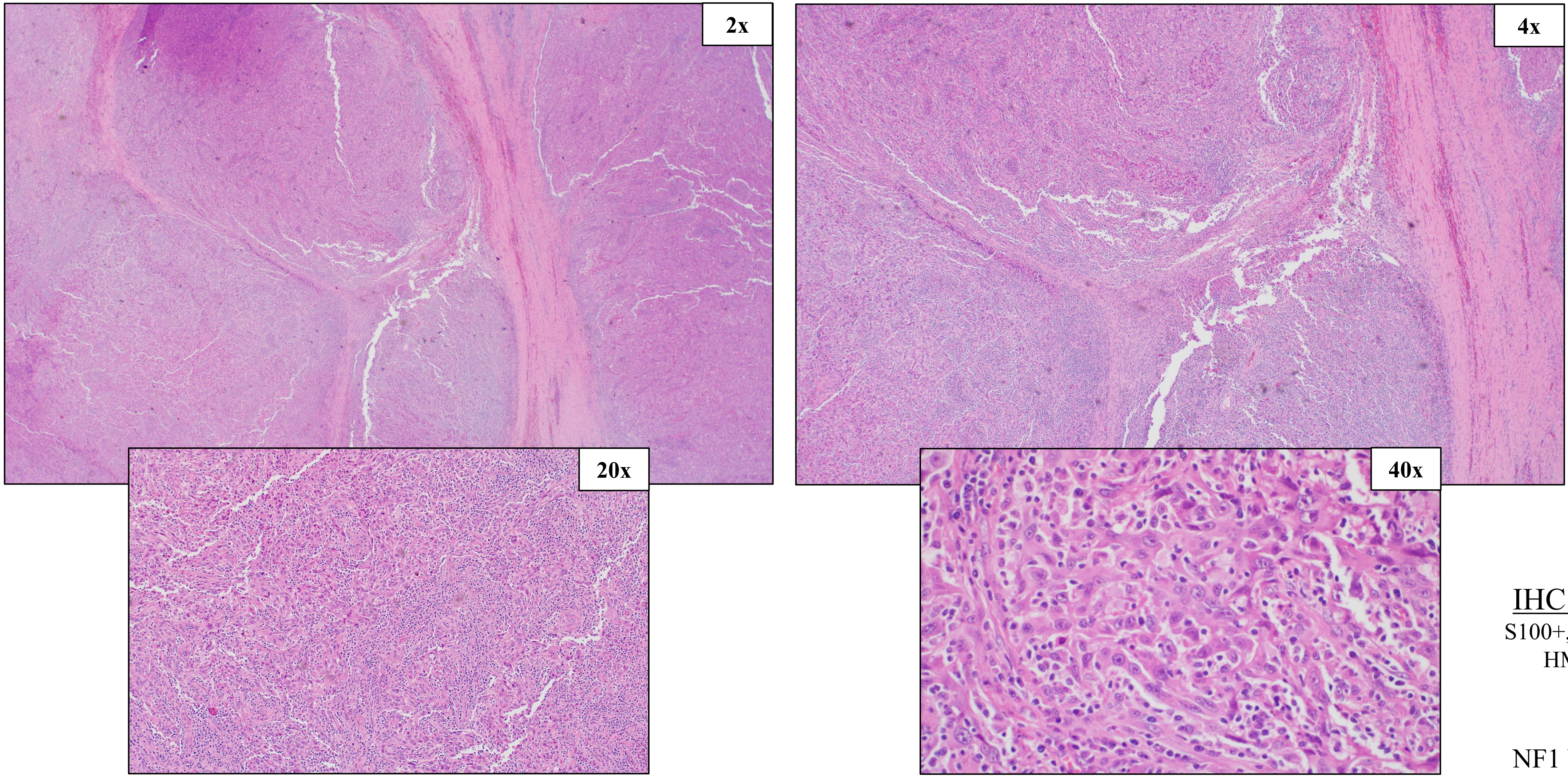

IHC Stains:
S100+, MelanA-, HMB45-

NF1 W221/Q282

HCI-CM005, Cutaneous Melanoma

<u>SNV (%VAF)</u>

NF1 c.662G>A, p.W221* (47%)
NF1 c.844C>T, p. Q282* (49%)
TP53 c.637C>T, p.R213* (99%)

<u>CNV</u>

Amplification: BRCA1
Loss: ARID1B, MET, PTEN, BRCA2, RB1, TP53

<u>PDX Tumor Histology</u>

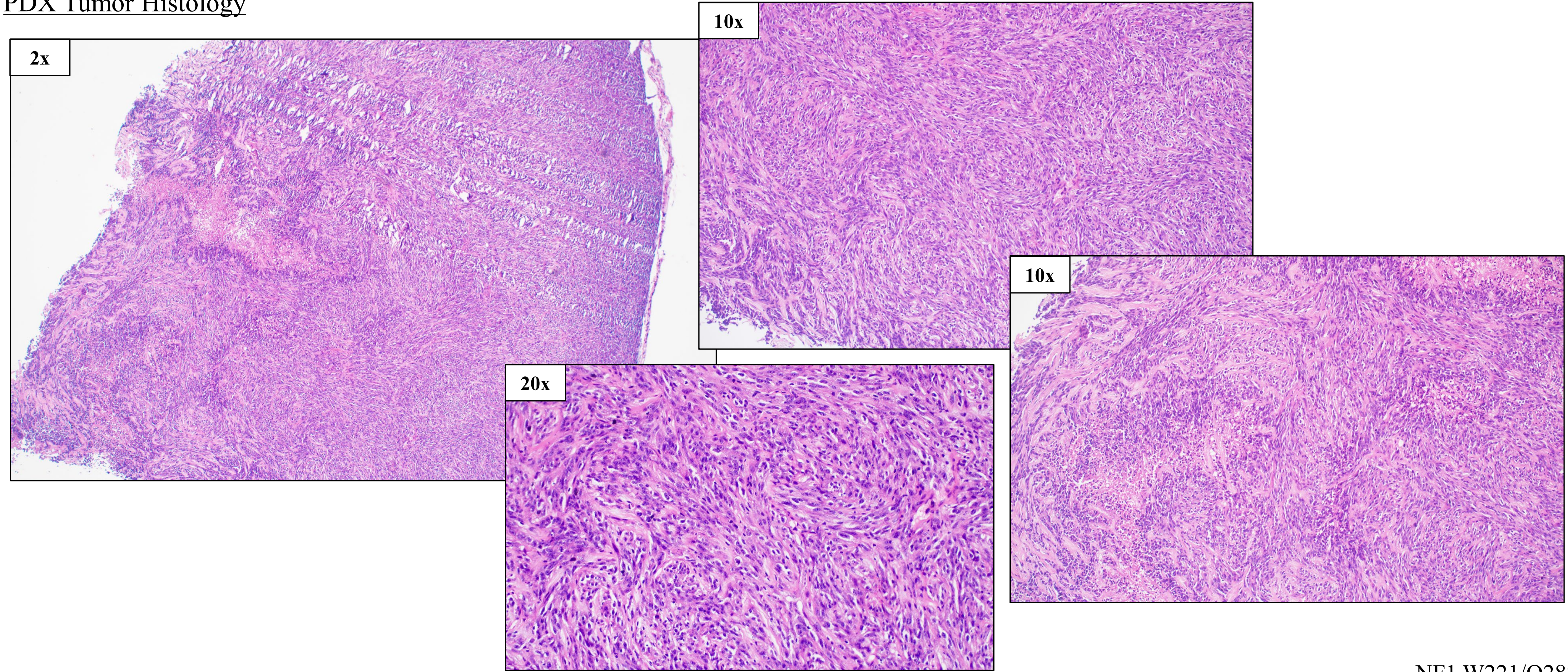

NF1 W221/Q282

HCI-CM019, Cutaneous Melanoma (from same patient as HCI-CM004)

Primary Tumor Location	PDX Tumor Location	Other Known Metastatic Locations
Scalp	Right Posterior Back Lymph Node	Omentum, Right and Left Lower Lung Lobes, Supraclavicular and Posterior Neck Lymph Nodes

Clinical Tumor Histology

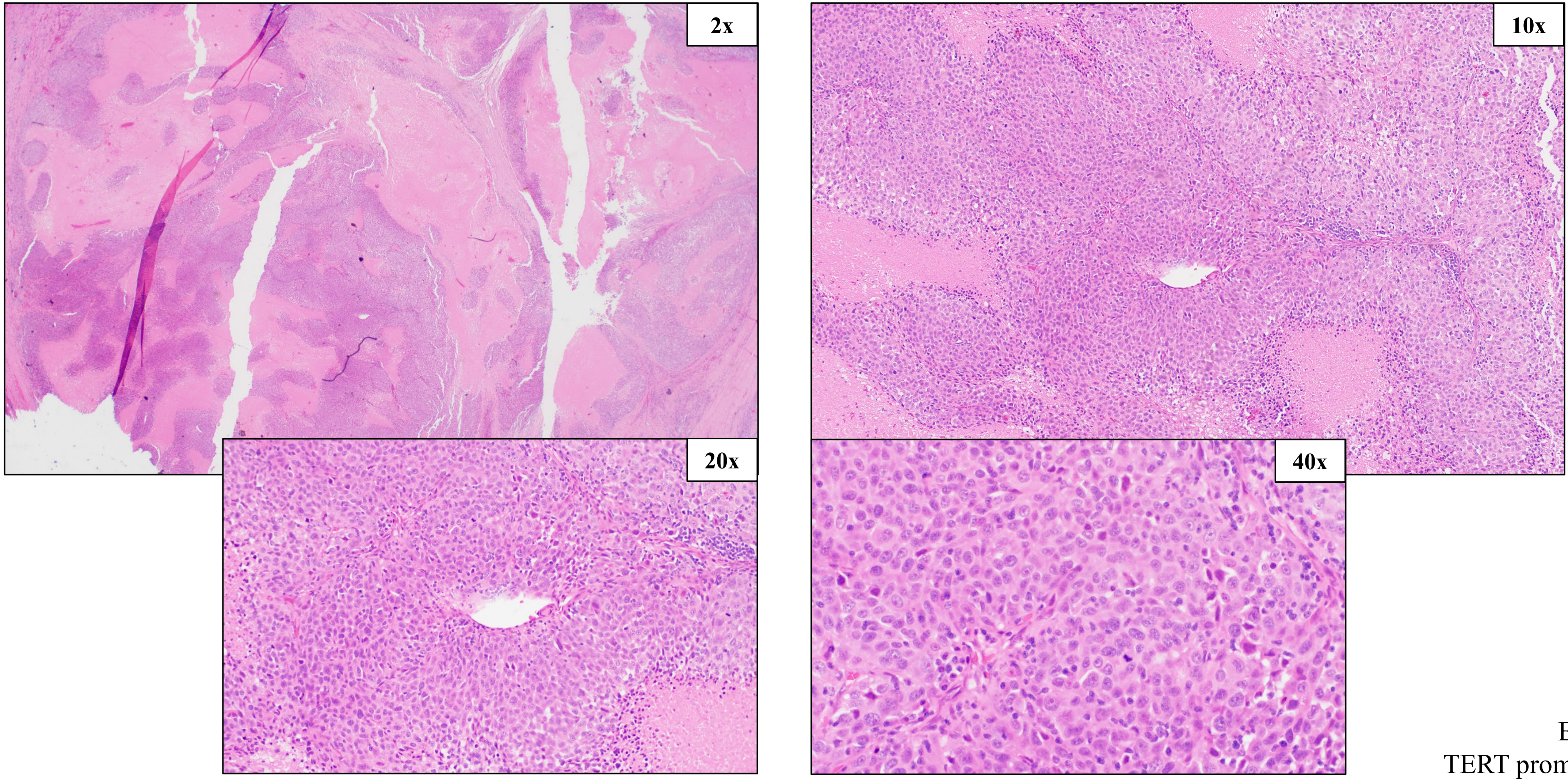

BRAF V600E,
TERT promoter mutation

HCI-CM019, Cutaneous Melanoma (from same patient as HCI-CM004)

<u>SNV (%VAF)</u>

BRAF c.1799T>A p.V600E (83%),
TERT chr5:1295250G>A (52%)

<u>CNV</u>

Amplification: MET
Loss: ARID1B, CDKN2A, PTEN, KRAS, MC1R

<u>PDX Tumor Histology, Passages 2 and 4</u>

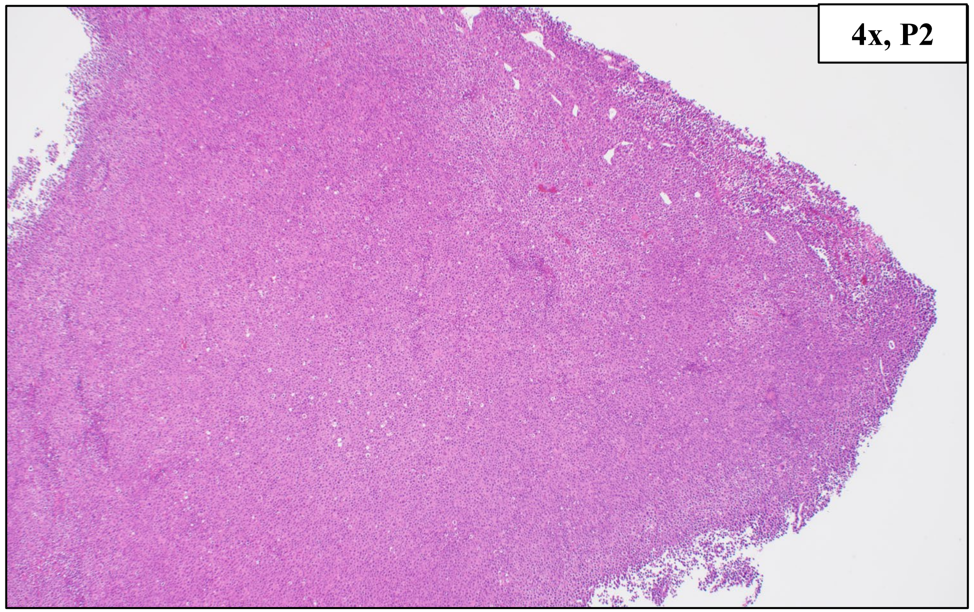

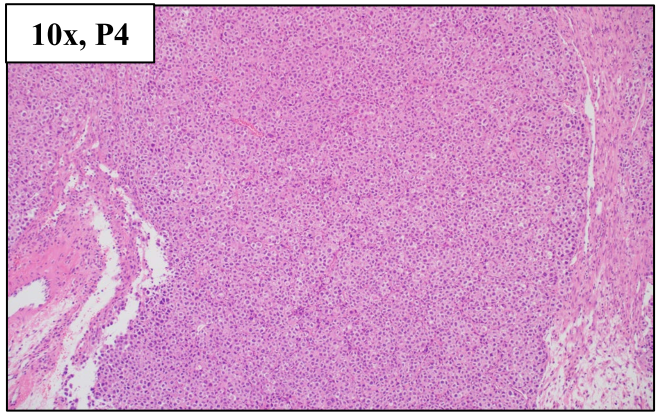

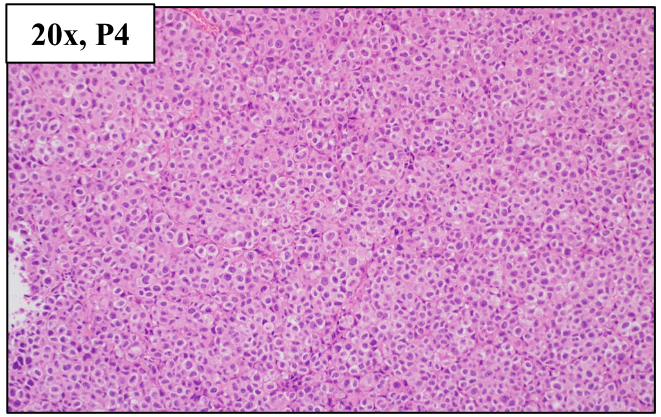

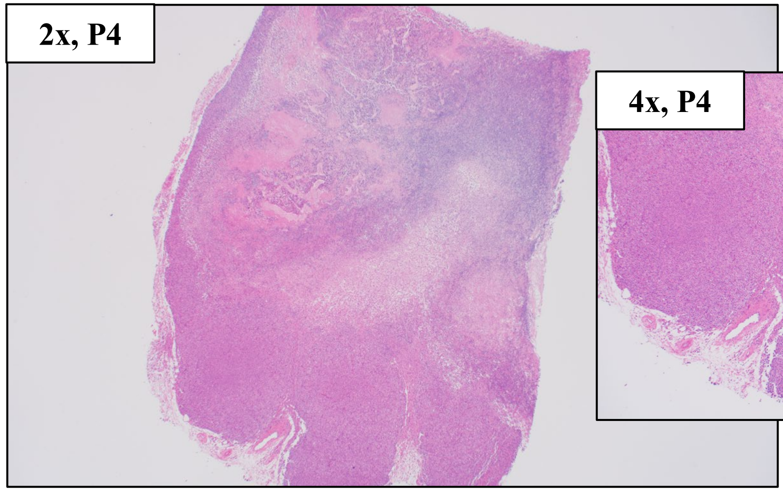

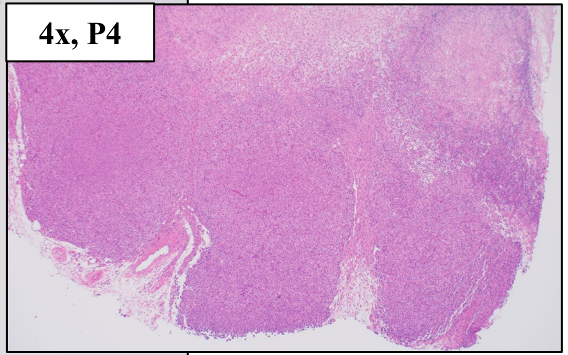

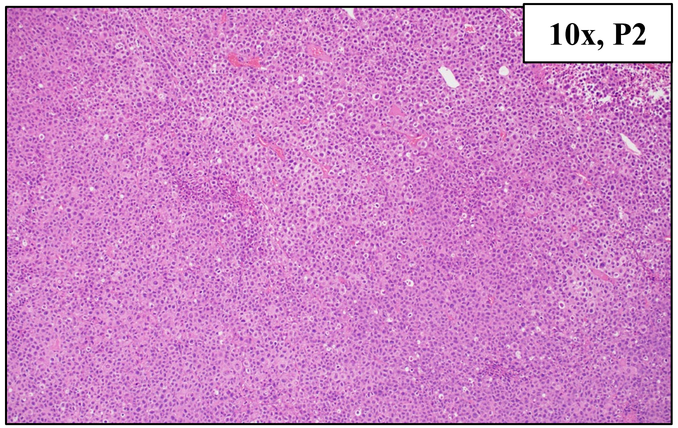

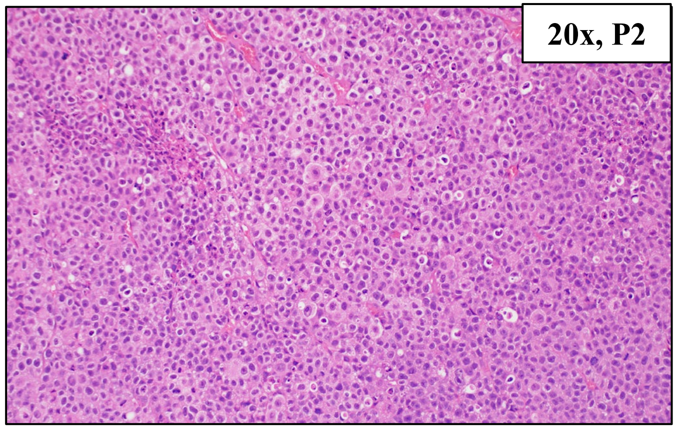

BRAF V600E, TERT promoter mutation

HCI-ASM020, Acral Melanoma (from same patient as HCI-ASM021)

Primary Tumor Location	PDX Tumor Location	Other Known Metastatic Locations
Left Hallux Toenail	Lower Abdominal Wall Lymph Node	Left Inguinal Lymph Nodes, Iliac Lymph nodes, Left Iliac Wing

Clinical Tumor Histology

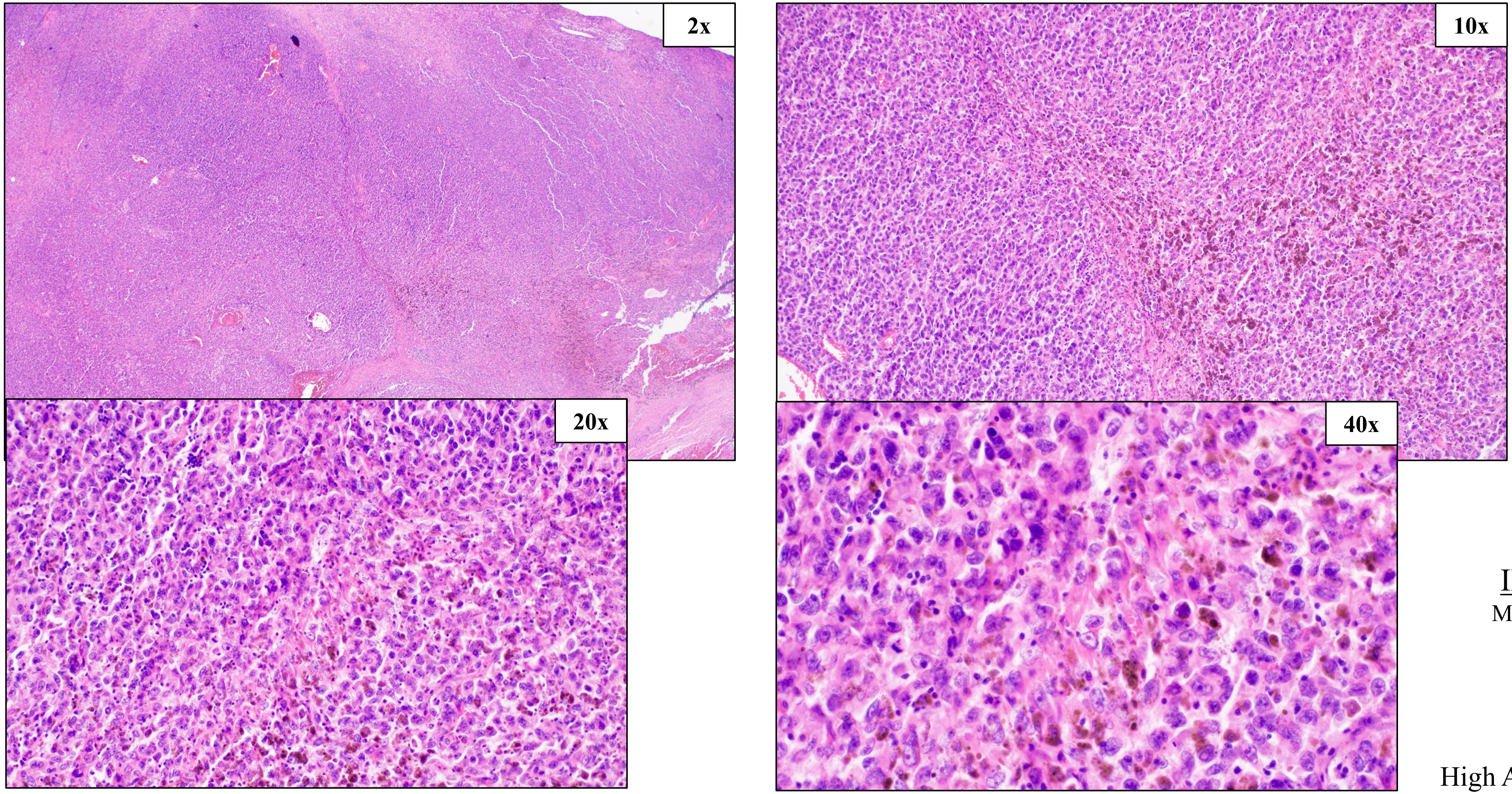

IHC Stains:

MelanA+, S100+

KIT N822K

High AMP: KIT, TERT

HCI-ASM020, Acral Melanoma (from same patient as HCI-ASM021)

SNV (%VAF)

KIT c.2466T>G, p.N822K (89%)
SPRED1 c.867dup, p.S290IfsTer10 (95%)

CNV

Deep Deletion: CDKN2A
Loss: ARID1B, SPRED1
Amplification: EGFR, MET, HRAS, MC1R, MAP2K2, SMARCA4
Amplification ≥8 copies: KIT, TERT, MAP2K1

PDX Tumor Histology, Passages 2 and 5

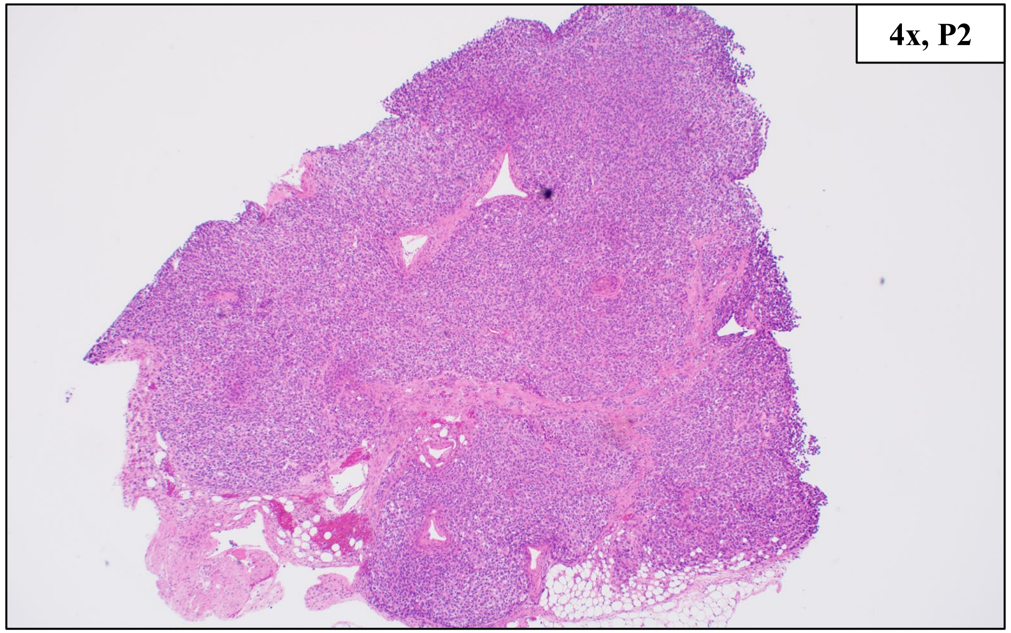

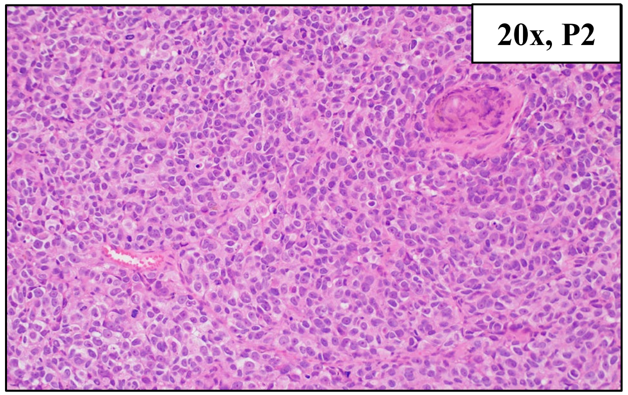

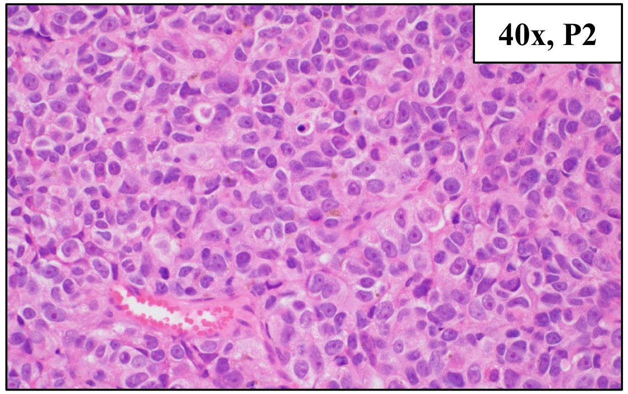

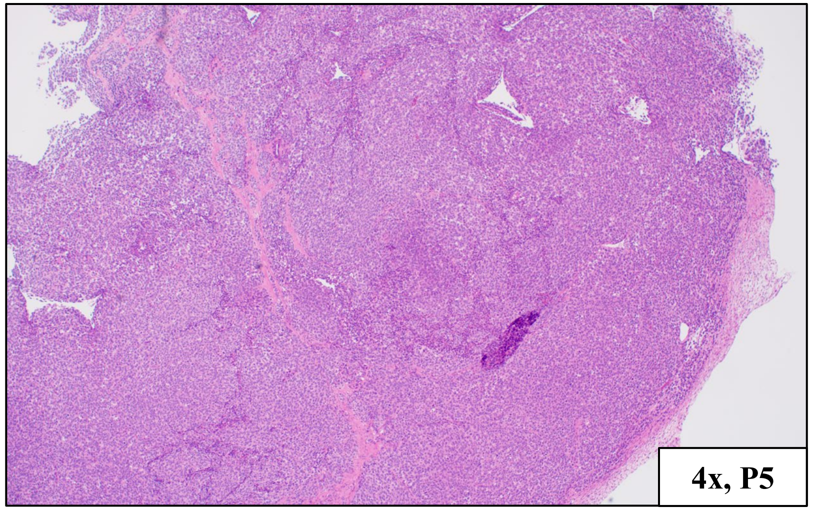

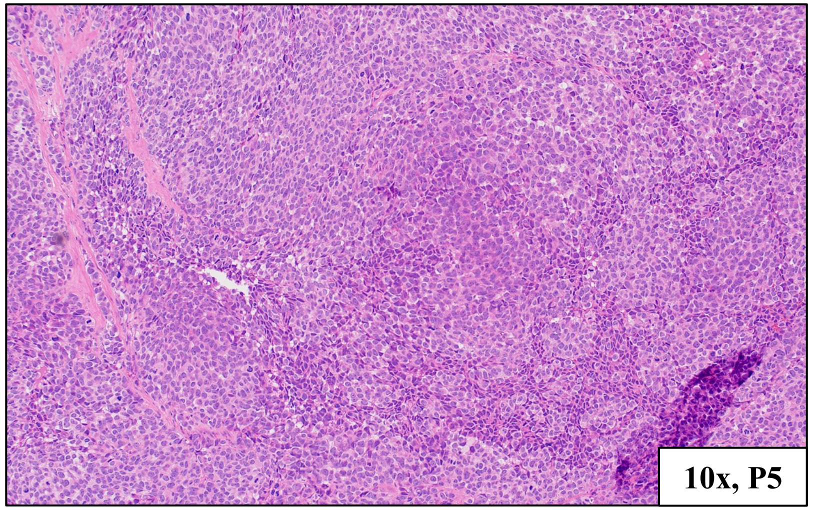

KIT N822K
High AMP: KIT, TERT

HCI-ASM021, Acral Melanoma (from same patient as HCI-ASM020)

Primary Tumor Location

Left Hallux Toenail

PDX Tumor Location

Left Great Toe

Other Known Metastatic Locations

Left Lower Abdominal Wall and Inguinal Lymph Nodes, Iliac Lymph nodes, Left Iliac Wing

Clinical Tumor Histology

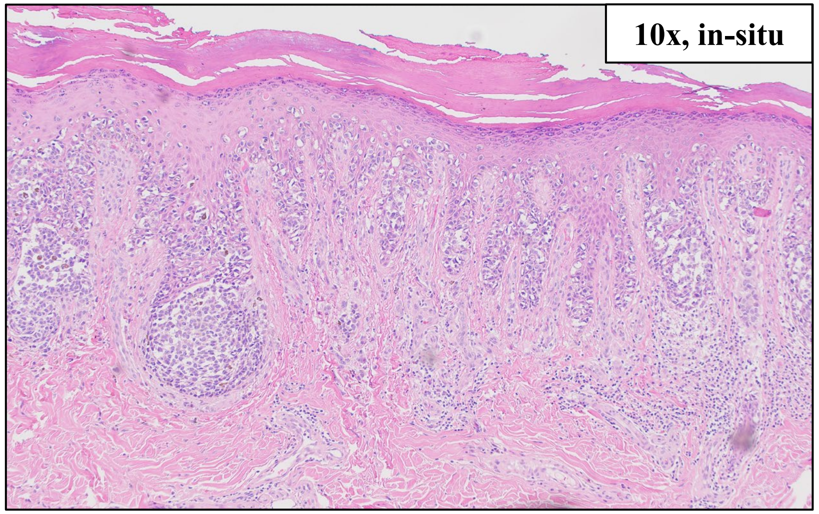

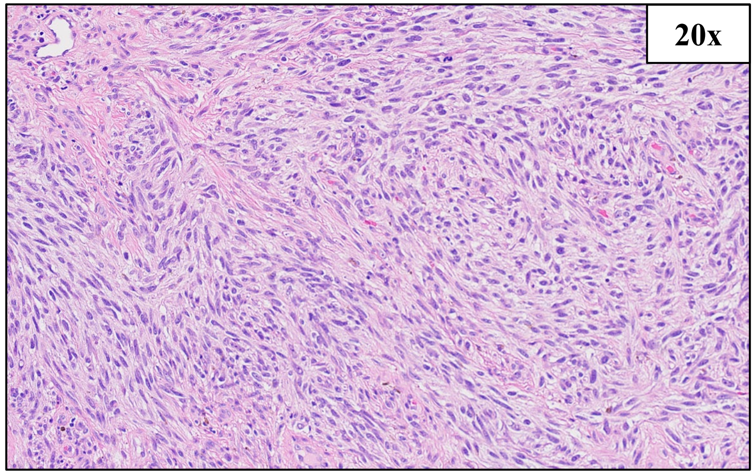

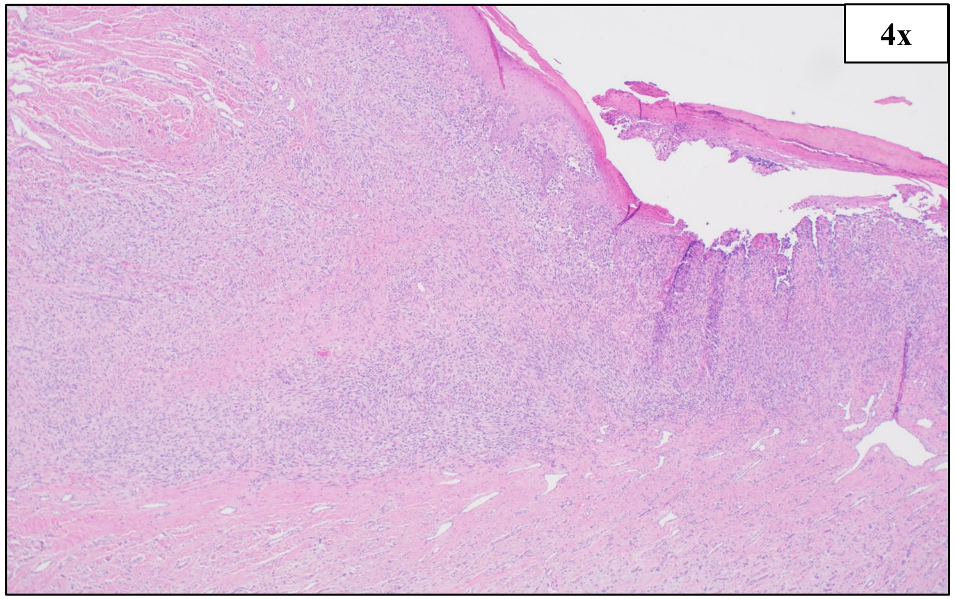

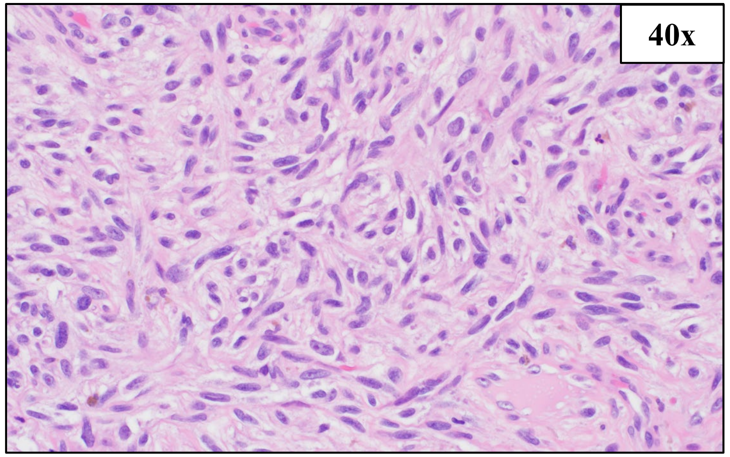

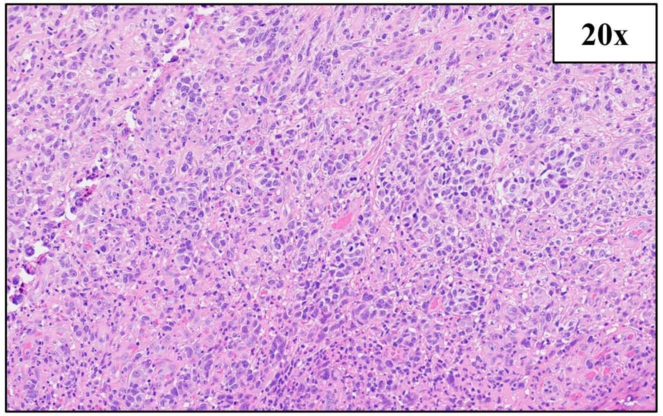

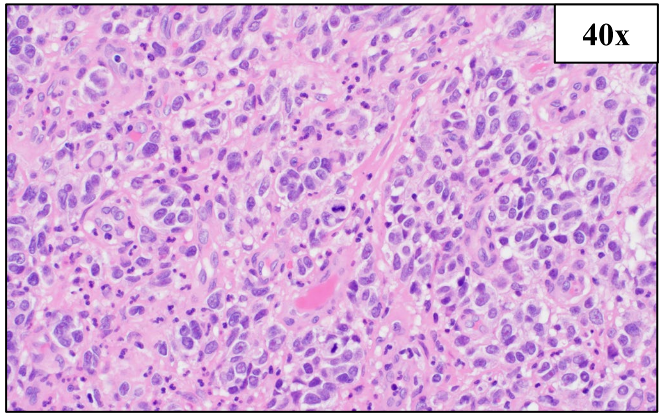

IHC Stains:

MelanA+, S100+

BRAF V600E, TERT promoter mutation,
High Amp: SMARCA4

HCI-ASM021, Acral Melanoma (from same patient as HCI-ASM020)

SNV (%VAF)

BRAF c.1799T>A, p.V600E (63%)
TERT chr5:1295250G>A (99%)

CNV

Amplification ≥8 copies: SMARCA4
Amplification: TERT, MET, BRAF, KRAS, CDK4, MC1R
Deep Deletion: CDKN2A

PDX Tumor Histology, Passage 3

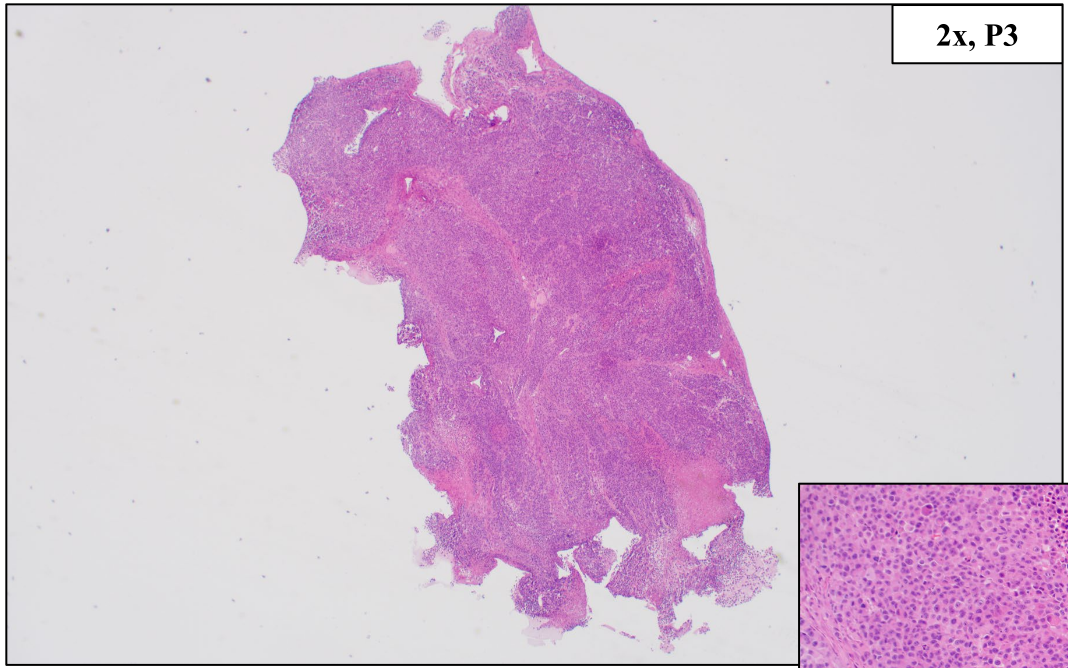

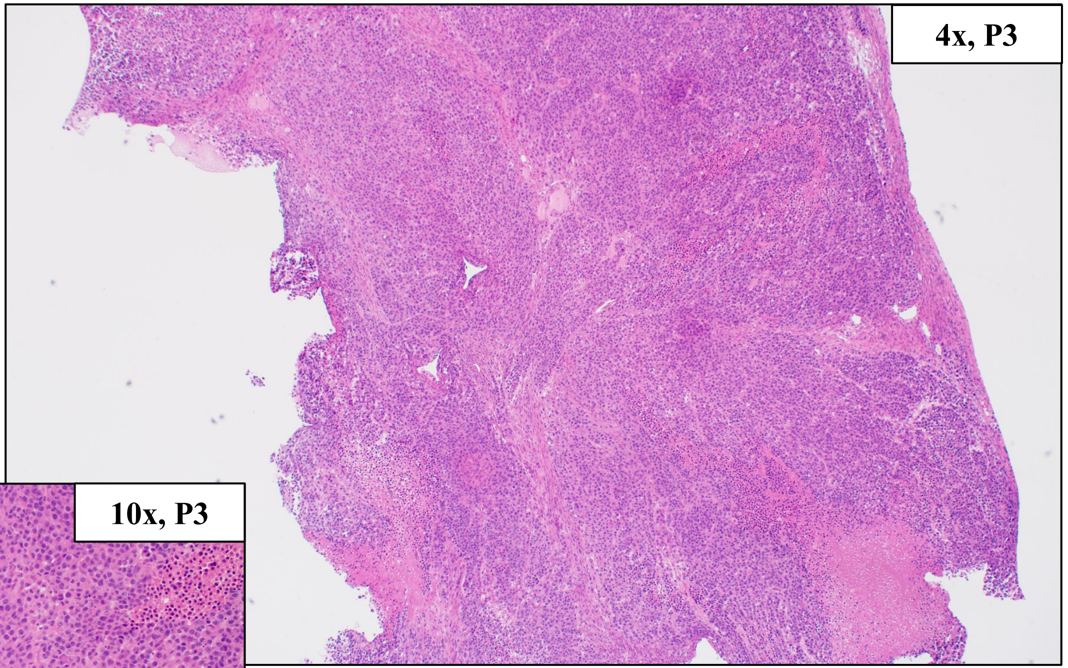

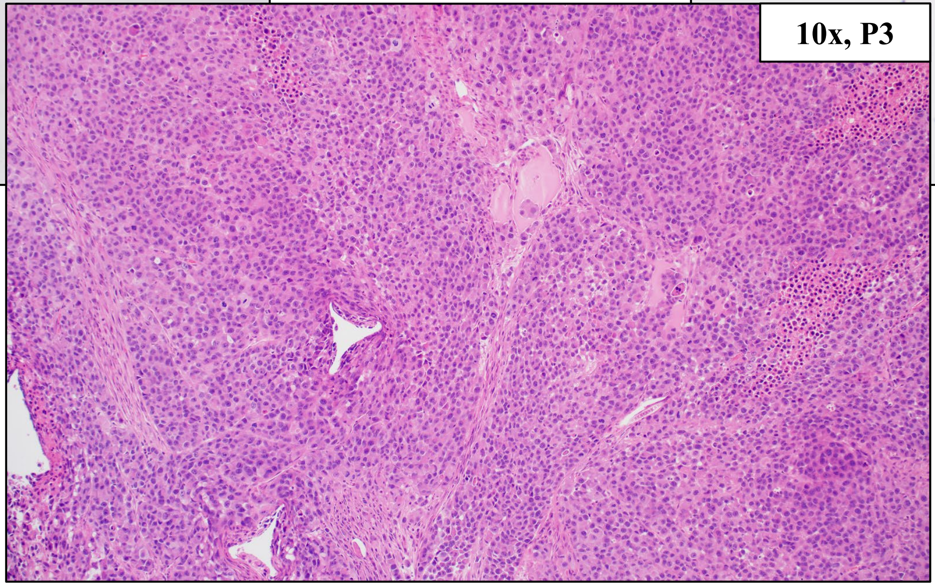

BRAF V600E, TERT promoter mutation,
High Amp: SMARCA4

HCI-CM053, Cutaneous Melanoma

Primary Tumor Location	PDX Tumor Location	Other Known Metastatic Locations
L Shoulder	L Axillary Lymph Node	None

Clinical Tumor Histology

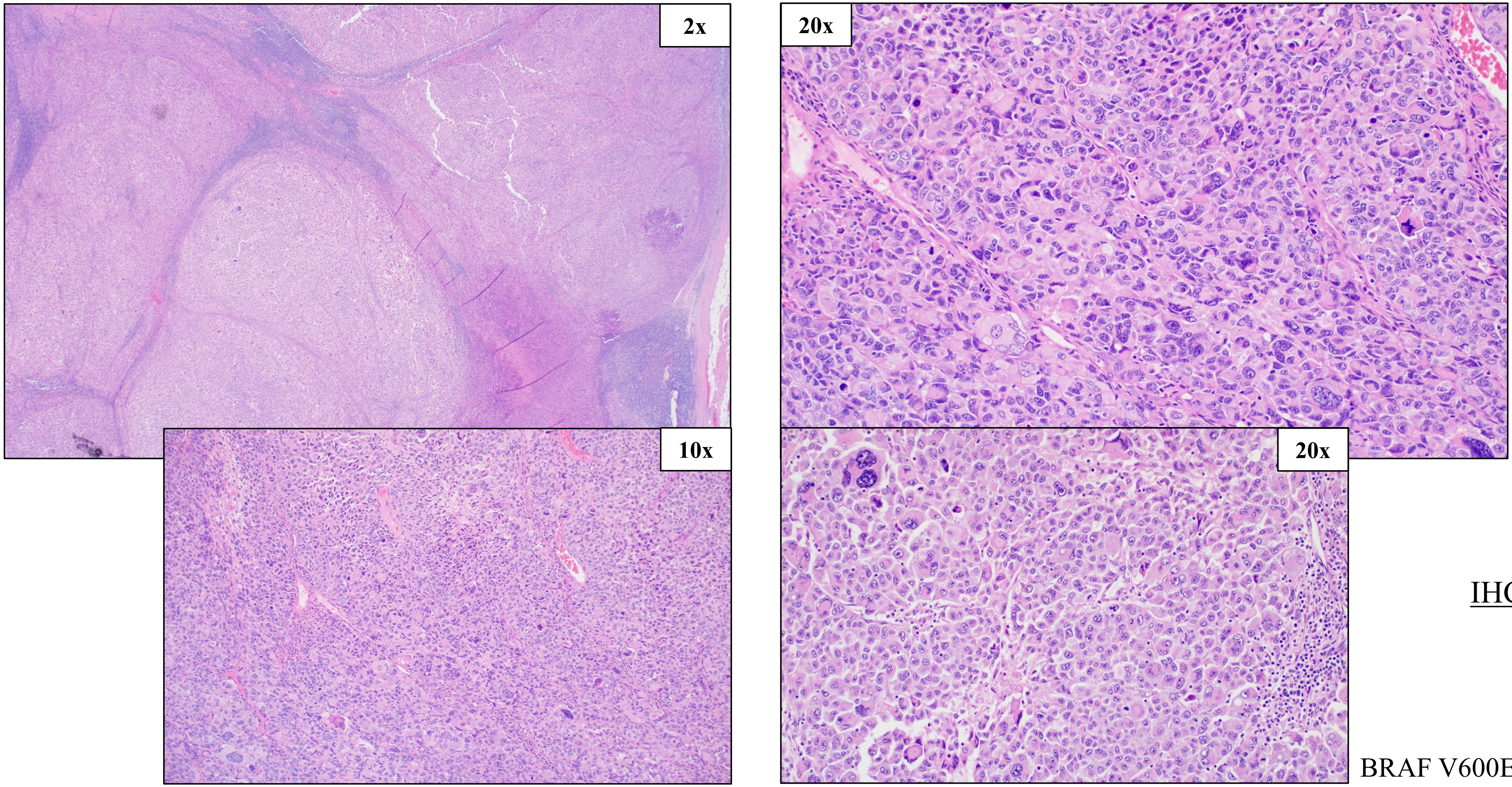

IHC Stains:
S100+

BRAF V600E, High Amp: TERT

HCI-CM053, Cutaneous Melanoma

SNV (%VAF)

BRAF c.1799T>A, p.V600E (50%)
MC1R c.86dup, p.N29KfsTer14 (35%)
TP53 c.722C>T, p.S241F (99%)
RB1 c.802G>T, p.E268* (99%)

CNV

Amplification ≥8 copies: TERT
Loss: PDGFRA, KIT, PTEN, CCND1, TP53

PDX Tumor Histology, Passages 2 and 4

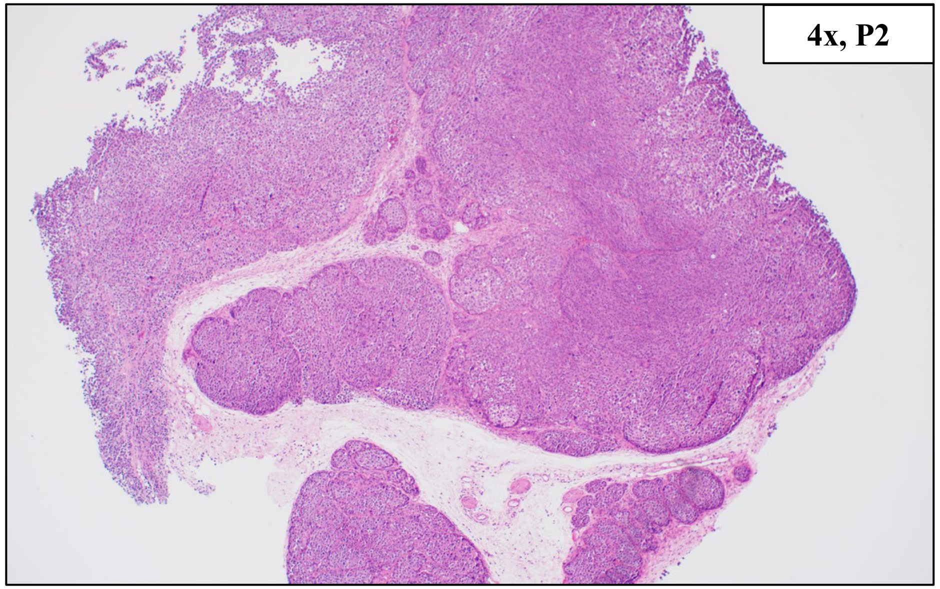

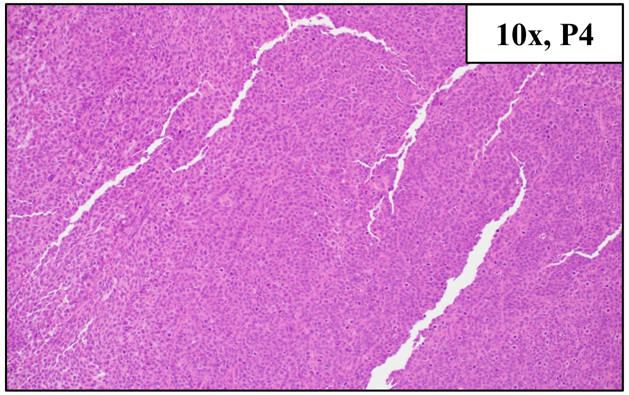

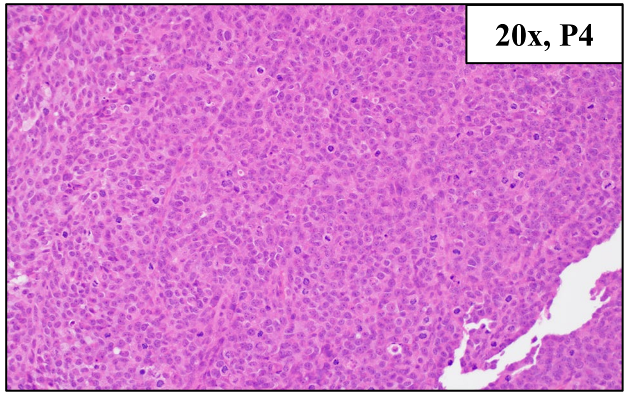

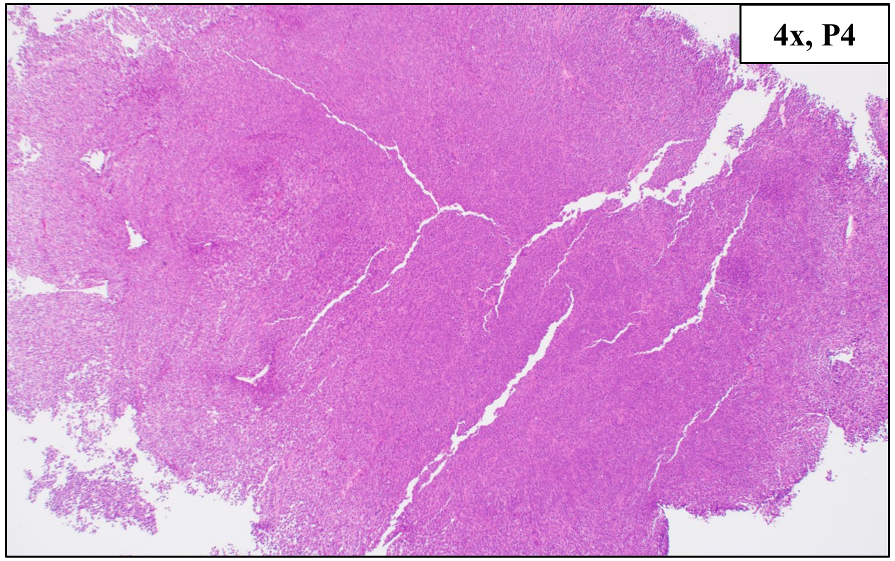

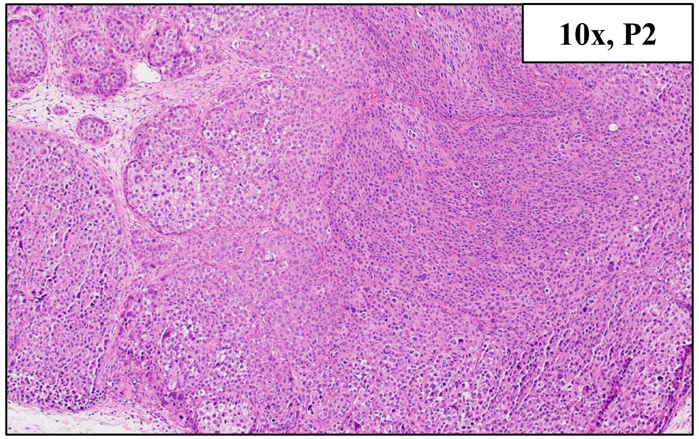

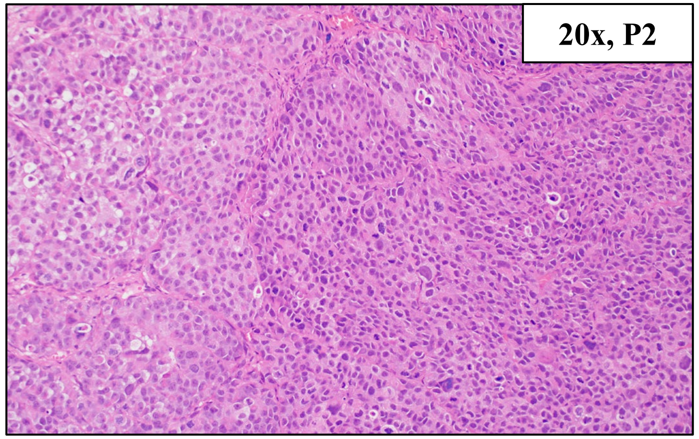

BRAF V600E, High Amp: TERT

HCI-ASM084, Acral Melanoma

Primary Tumor Location	PDX Tumor Location	Other Known Metastatic Locations
Left Thumb Skin	Left Lower Back or Right Anterior Thigh	Bilateral Lungs, Right Upper Arm, Left Lower Back, Right Anterior Thigh

Clinical Tumor Histology

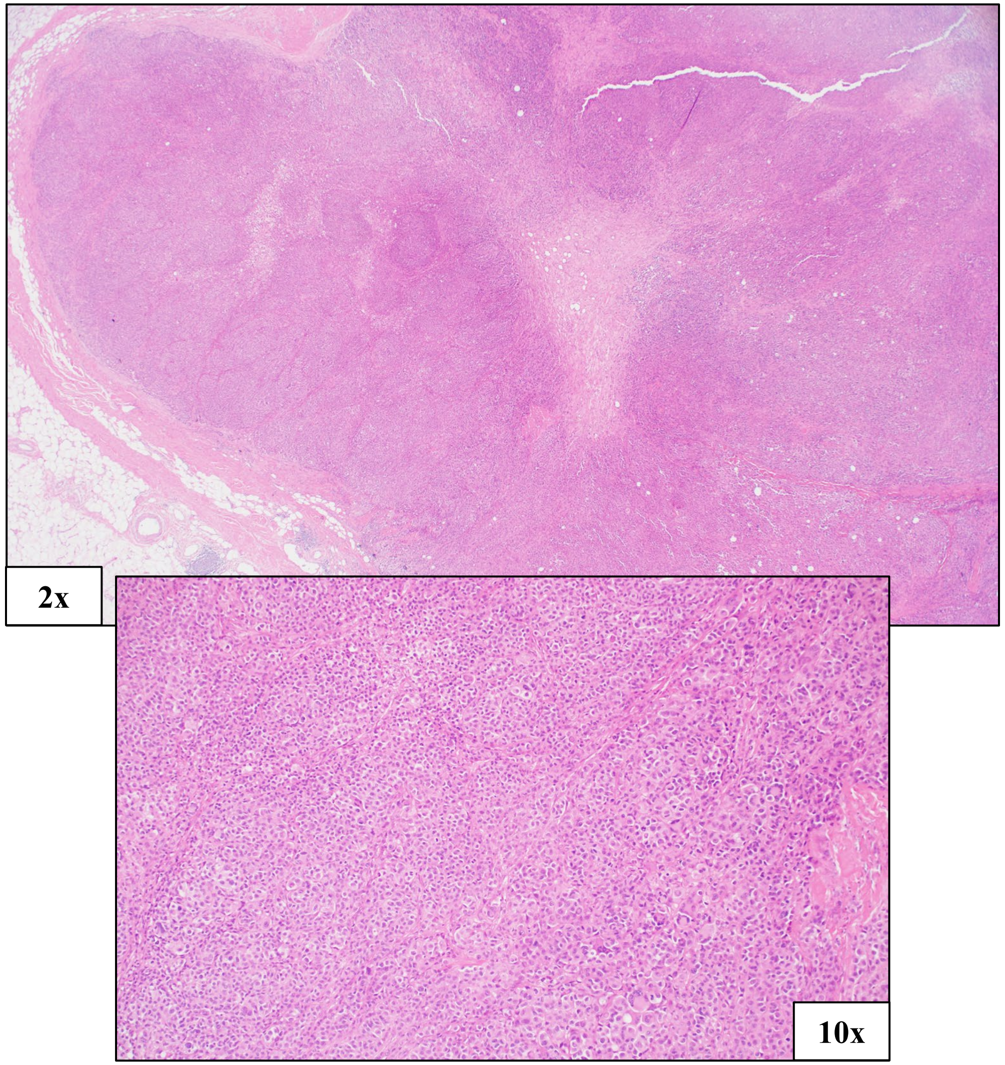

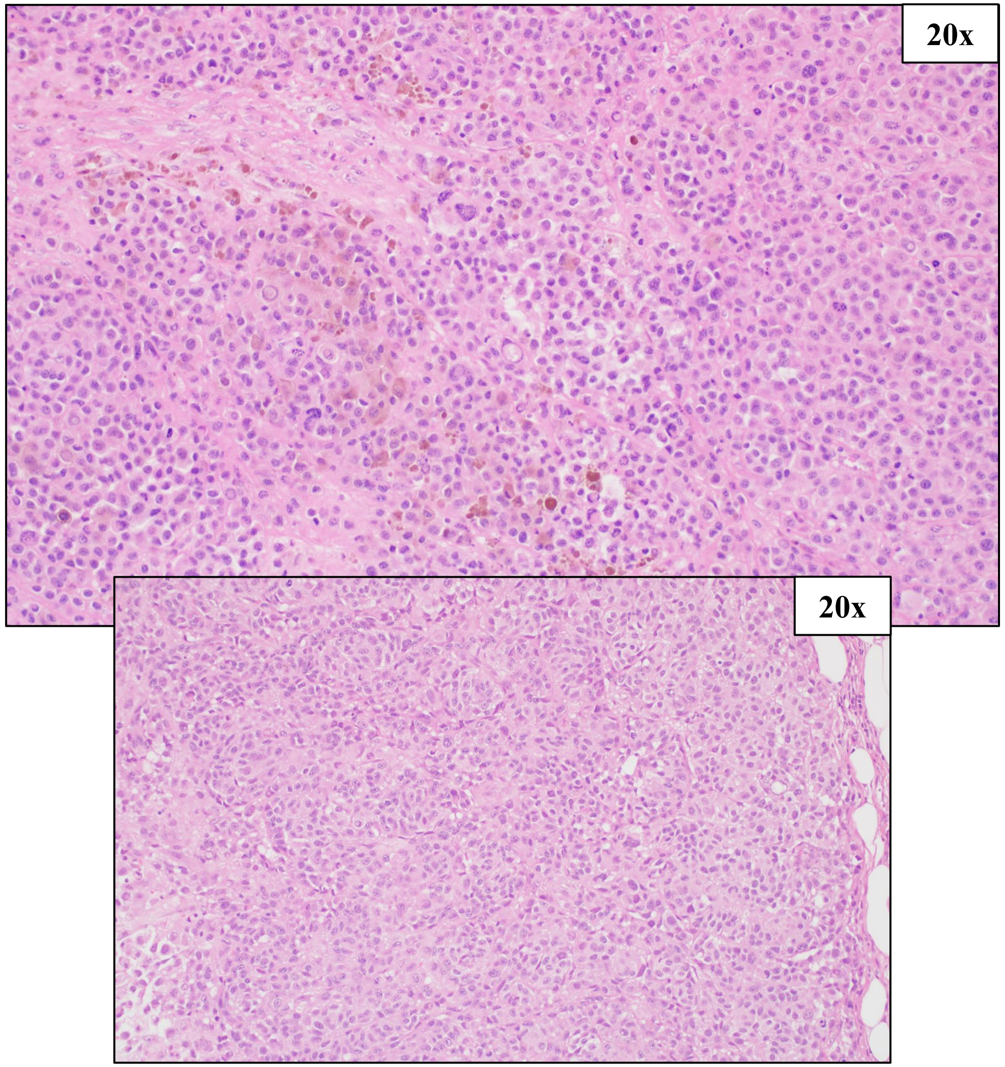

IHC Stains:

Not reported

BRAF G469A

HCI-ASM084, Acral Melanoma

SNV (%VAF)

BRAF c.1406G>C, p.G469A (69%)

CNV

Amplification: TERT, EGFR, MET, BRAF
Loss: ARID1A, PTEN, SPRED1
Deep Deletion: CDKN2A

PDX Tumor Histology, Passages 2 and 4

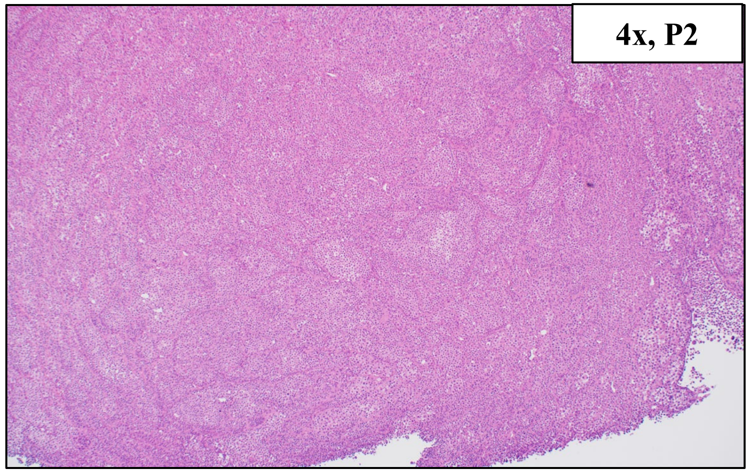

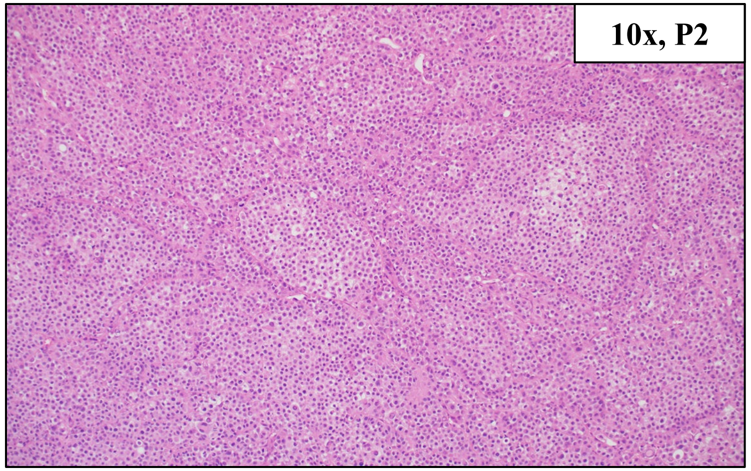

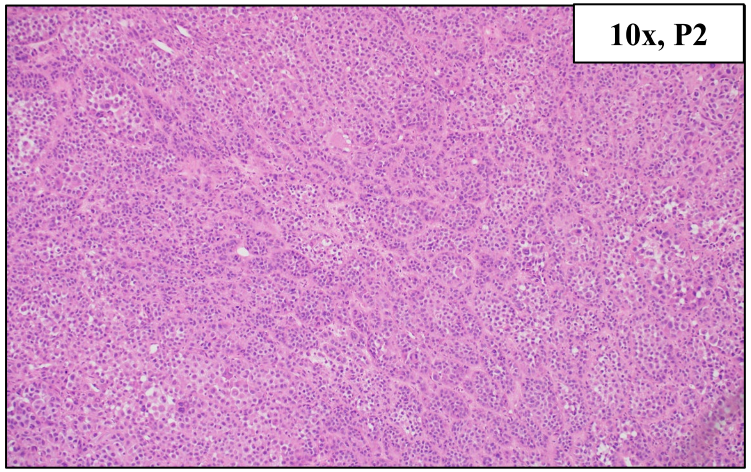

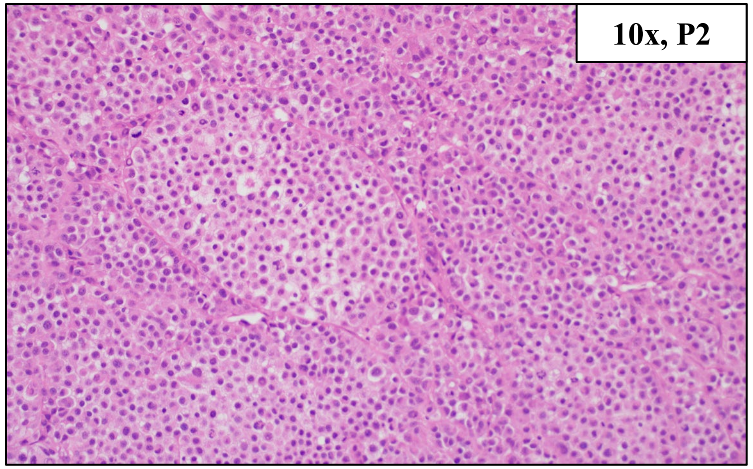

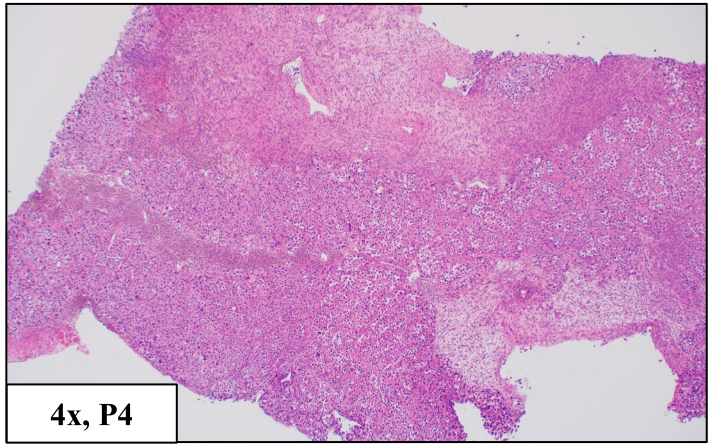

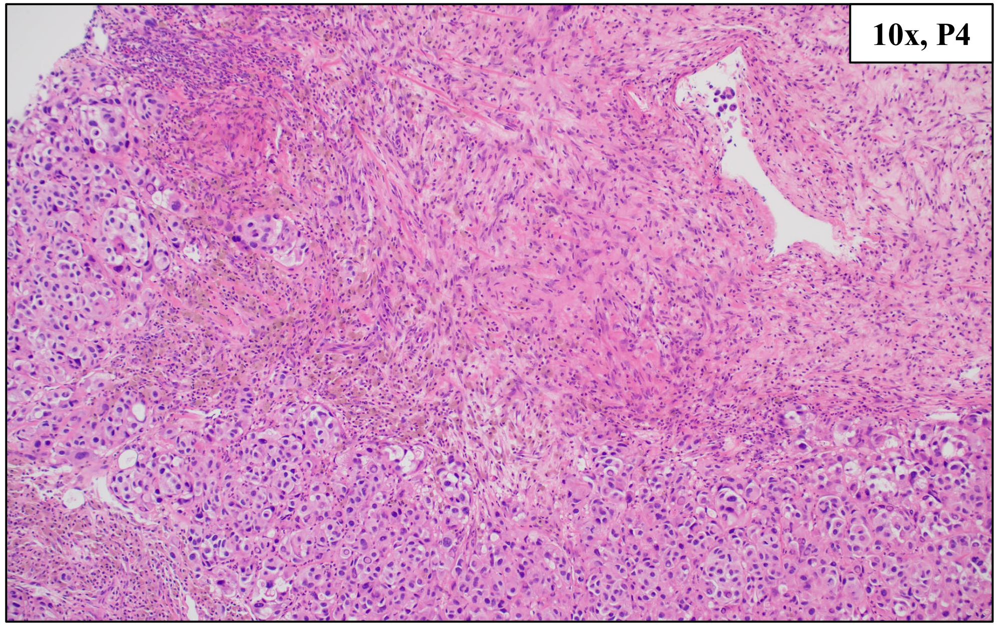

BRAF G469A

HCI-AM085, Acral Melanoma

Primary Tumor Location	PDX Tumor Location	Other Known Metastatic Locations
Right Thumb Skin	Right Middle Lobe Lung	none

Clinical Tumor Histology

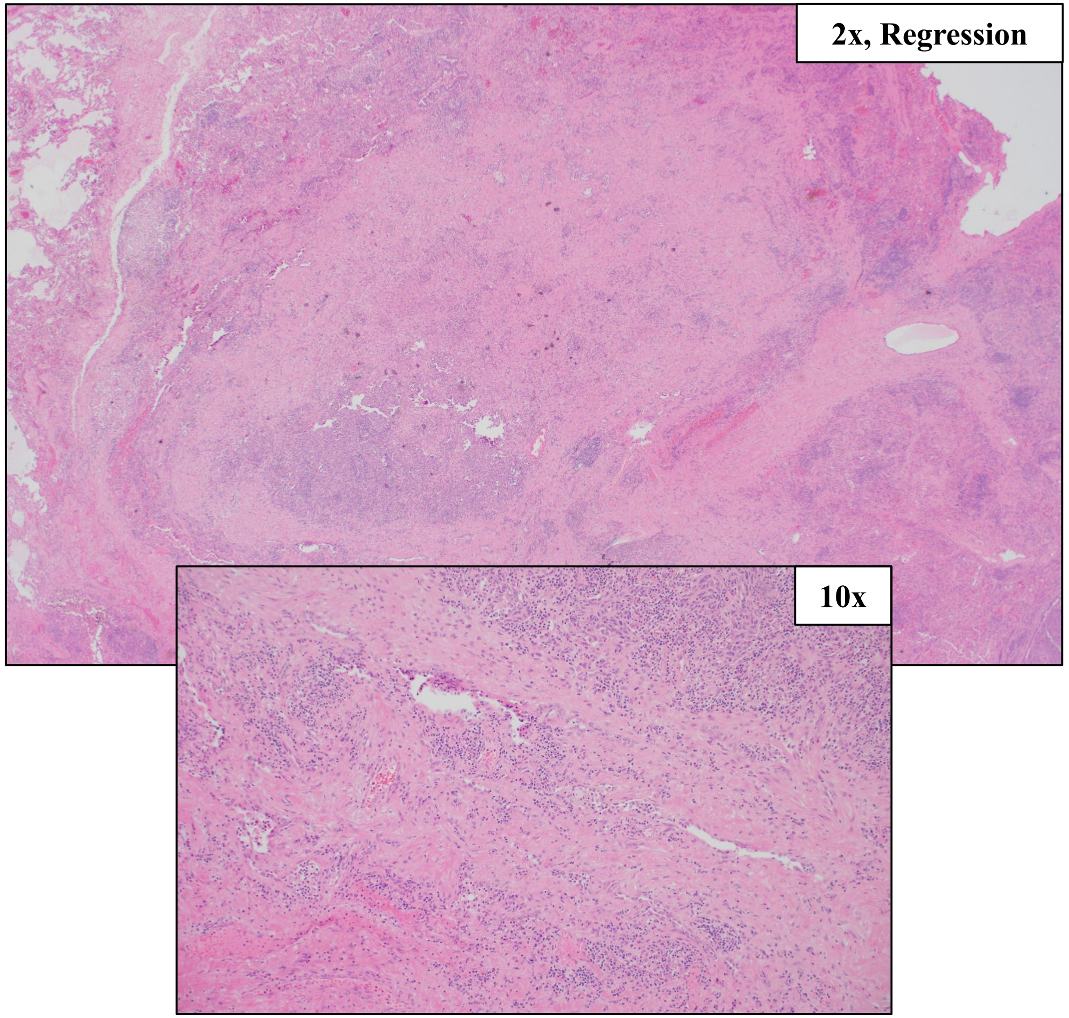

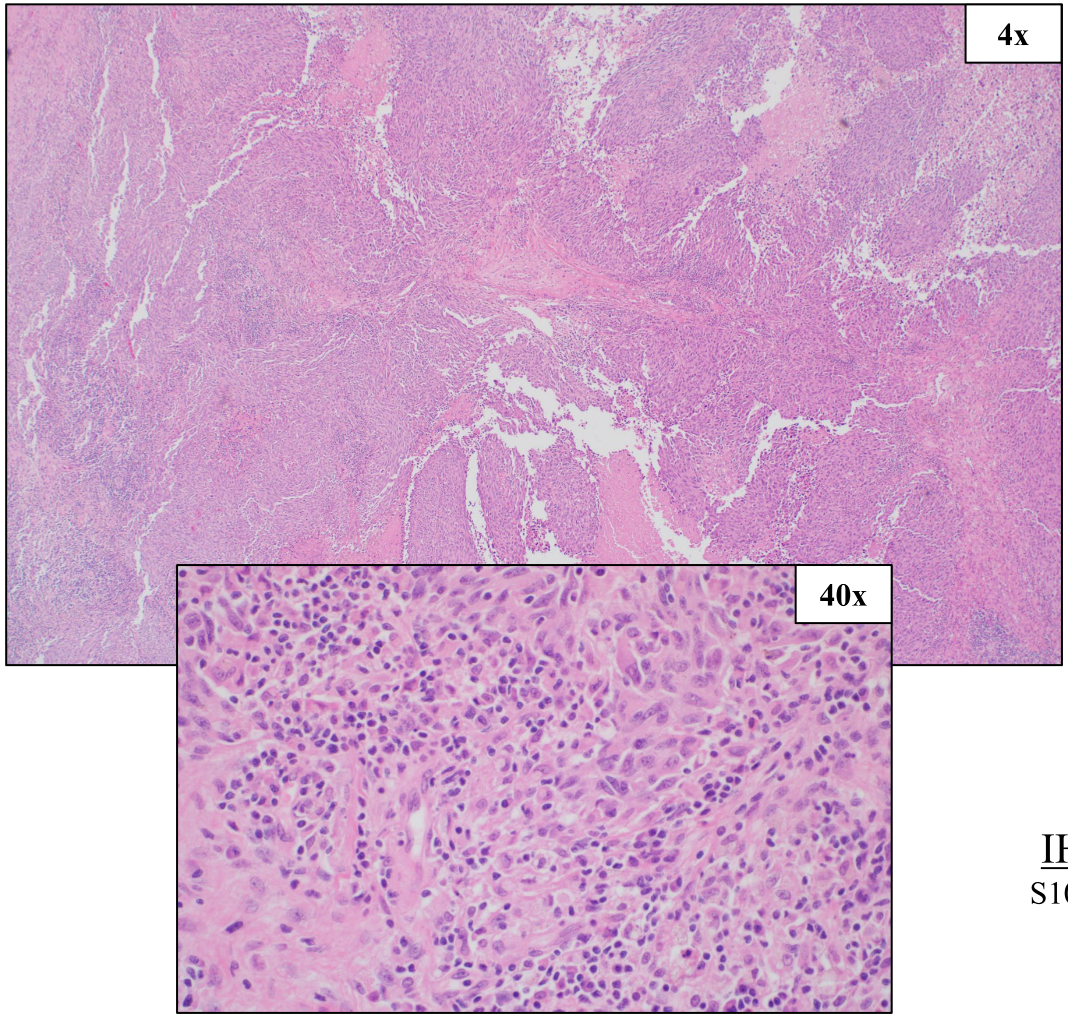

IHC Stains:
S100+, MelanA+

NF1 splice donor variant, High Amp: CDK4, MDM2, MC1R

HCI-AM085, Acral Melanoma

SNV (%VAF)

NF1 Splice Donor Variant c.4763_4772+4del (99%)

CNV

Amplification ≥8 copies: CDK4, MDM2, MC1R
Amplification: MITF, TERT, MET, HRAS
Loss: ARID1B, PTEN, NF1

PDX Tumor Histology, Passages 2 and 4

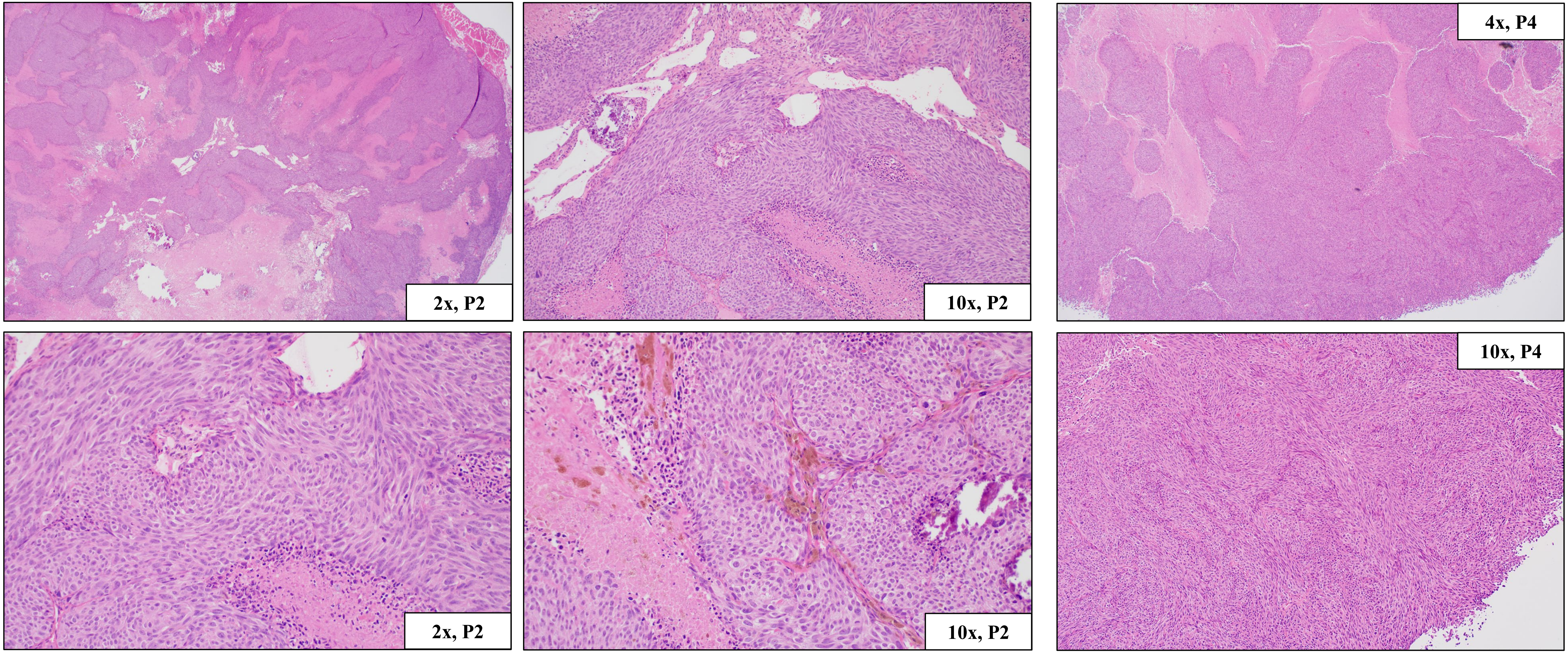

NF1 splice donor variant, High Amp: CDK4, MDM2, MC1R

HCI-AM086, Acral Melanoma (same patient as HCI-AM087)

Primary Tumor Location	PDX Tumor Location	Other Known Metastatic Locations
Left Great Toe Skin	Left Groin Soft Tissue	Left Inguinal Lymph Nodes, Epidural Spine, C2 Vertebral Lamina, Bilateral Lungs

Clinical Tumor Histology

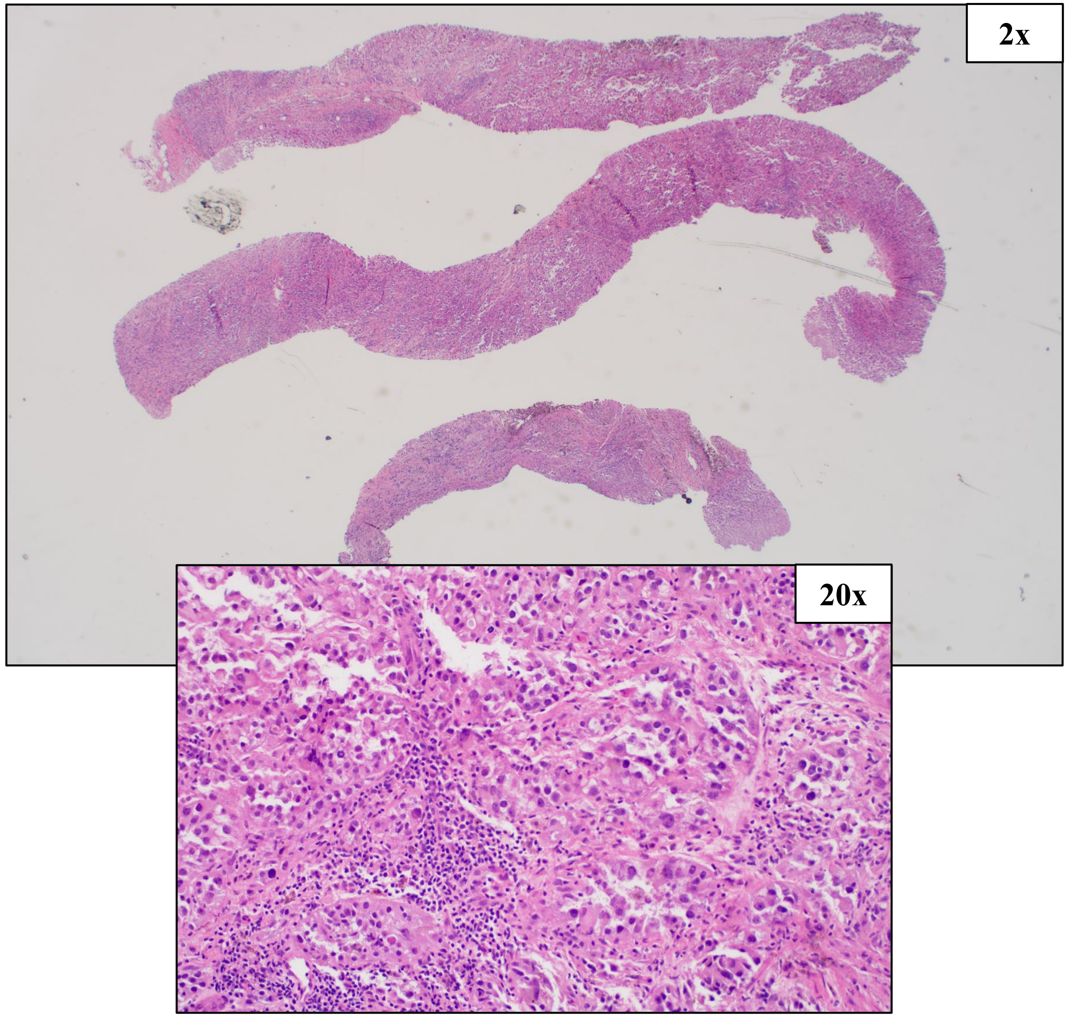

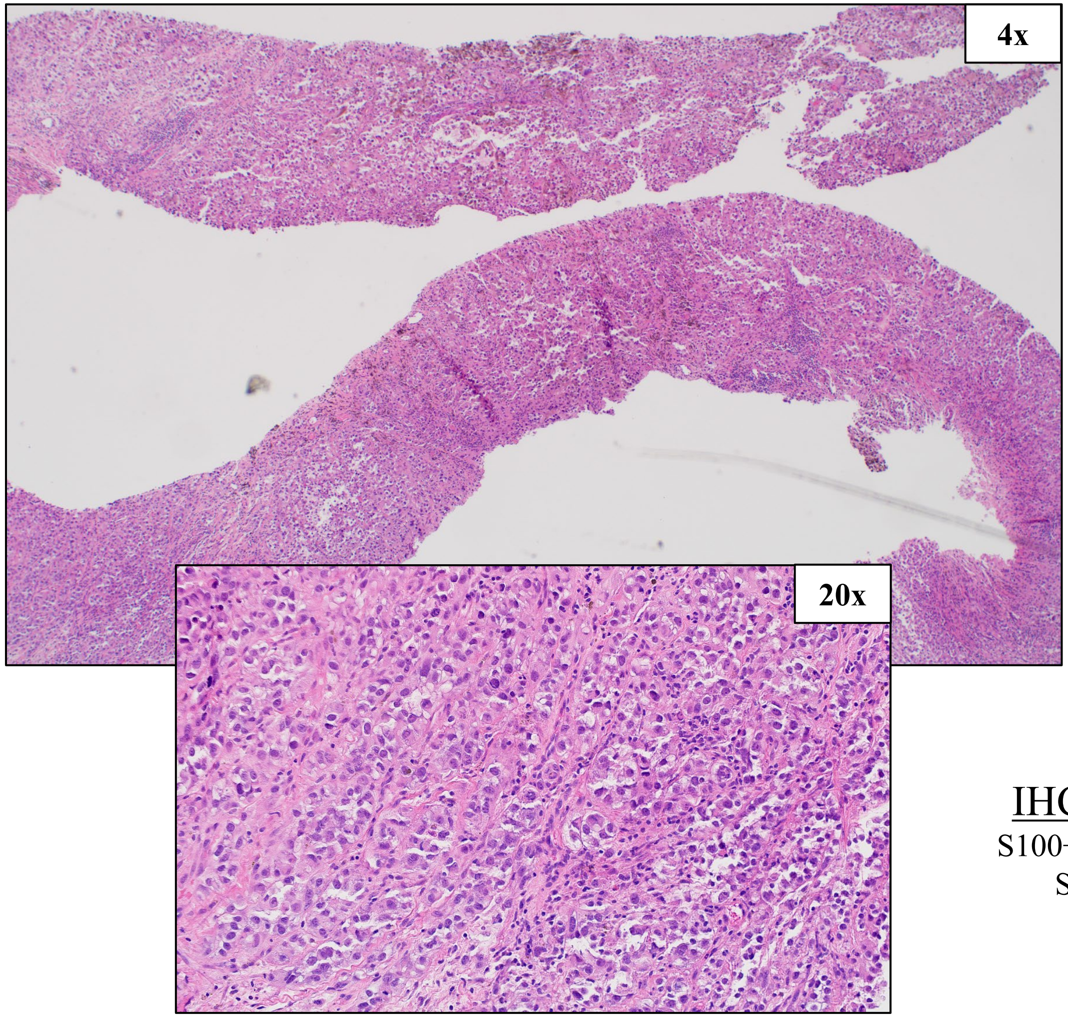

IHC Stains:
S100+, MelanA+, SOX10+

KRAS G12D, High Amp: CDK4, GAB2, MDM2

HCI-AM086, Acral Melanoma (same patient as HCI-AM087)

SNV (%VAF)

KRAS c.35G>A, p.G12D (83%)

CNV

Amplification ≥8 copies: GAB2, CDK4, MDM2
Amplification: MITF, HRAS, KRAS
Loss: CDKN2A, ARID2

PDX Tumor Histology, Passages 2 and 4

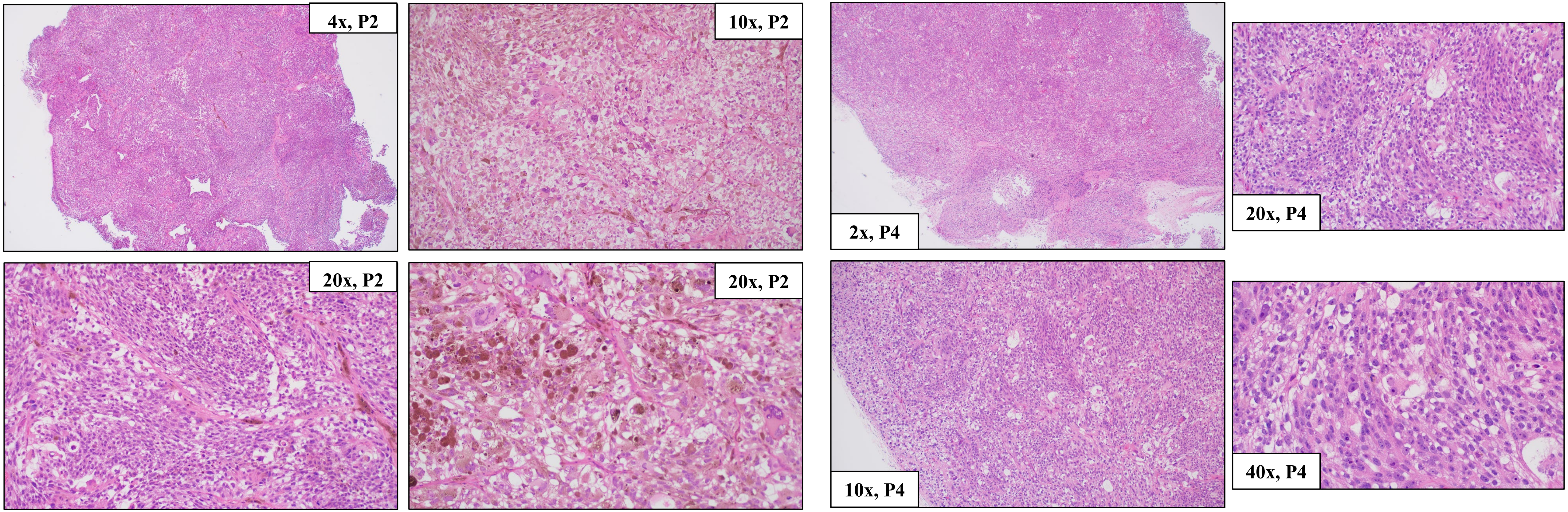

KRAS G12D, High Amp: CDK4, GAB2, MDM2

HCI-AM087, Acral Melanoma (same patient as HCI-AM086)

Primary Tumor Location	PDX Tumor Location	Other Known Metastatic Locations
Left Great Toe Skin	Left Groin Soft Tissue	Left Inguinal Lymph Nodes, Epidural Spine, C2 Vertebral Lamina, Bilateral Lungs

Clinical Tumor Histology

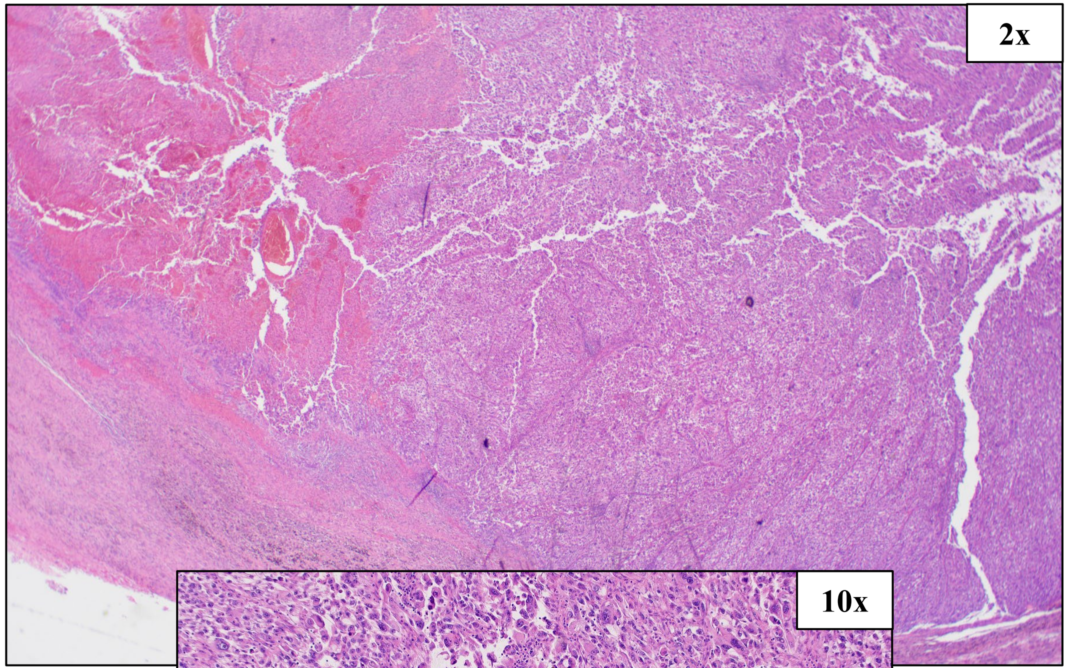

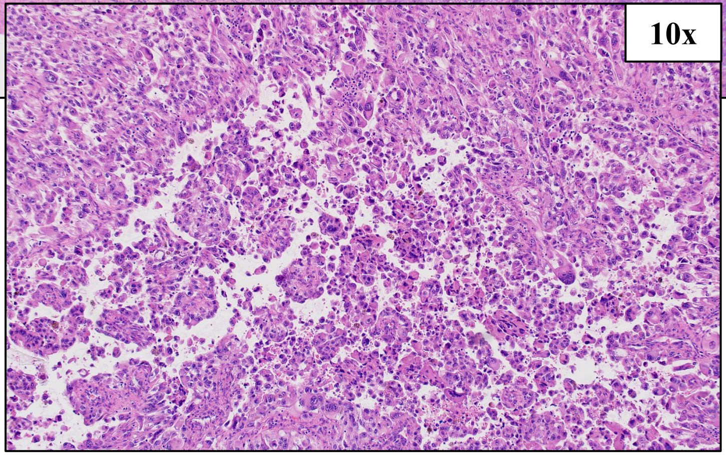

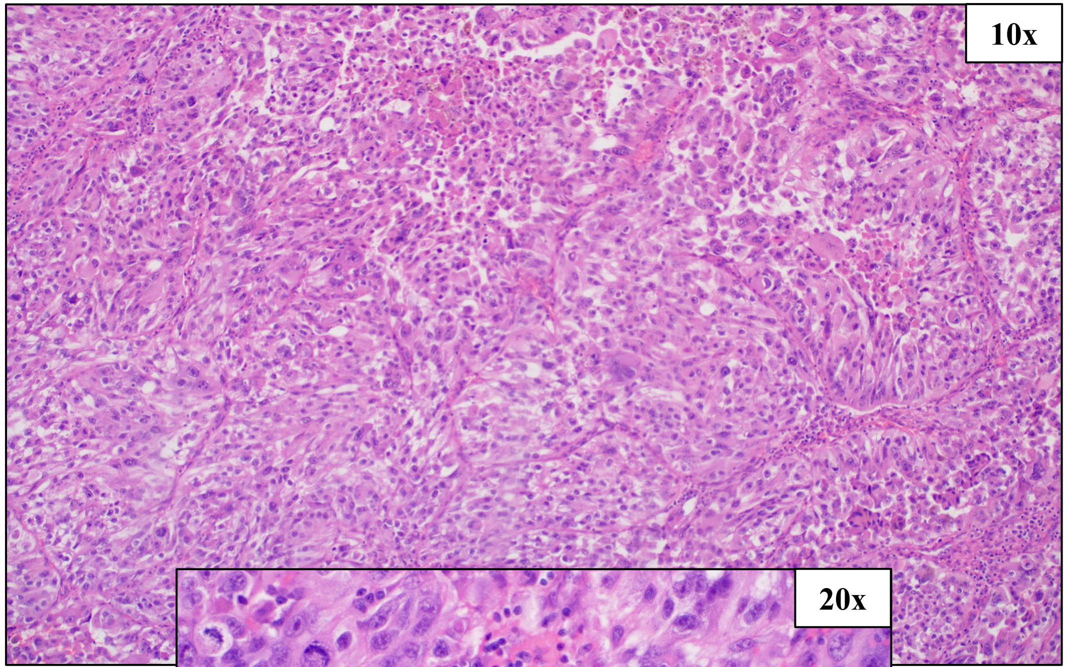

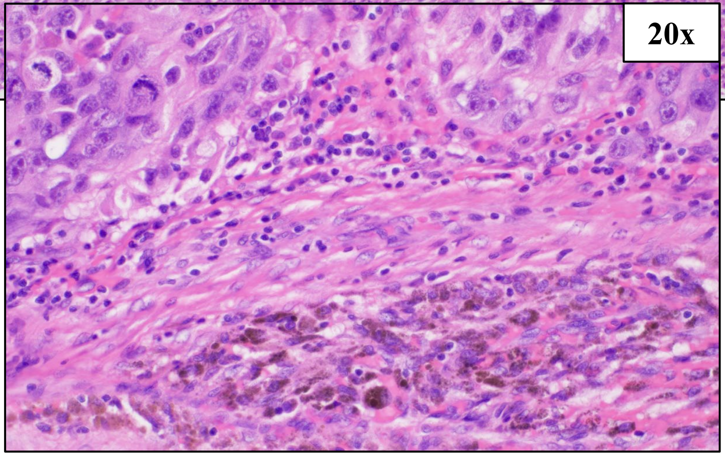

IHC Stains:
S100+, MelanA+,
SOX10+

KRAS G12D, High Amp: CDK4, GAB2, MDM2

HCI-AM087, Acral Melanoma (same patient as HCI-AM086)

SNV (%VAF)

KRAS c.35G>A, p.G12D (74%)

CNV

Amplification ≥8 copies: GAB2, CDK4, MDM2
Amplification: MET, KRAS, BRCA1
Loss: PDGFRA, KIT, ARID1B, CDKN3A, ARID2

PDX Tumor Histology, Passages 2 and 5

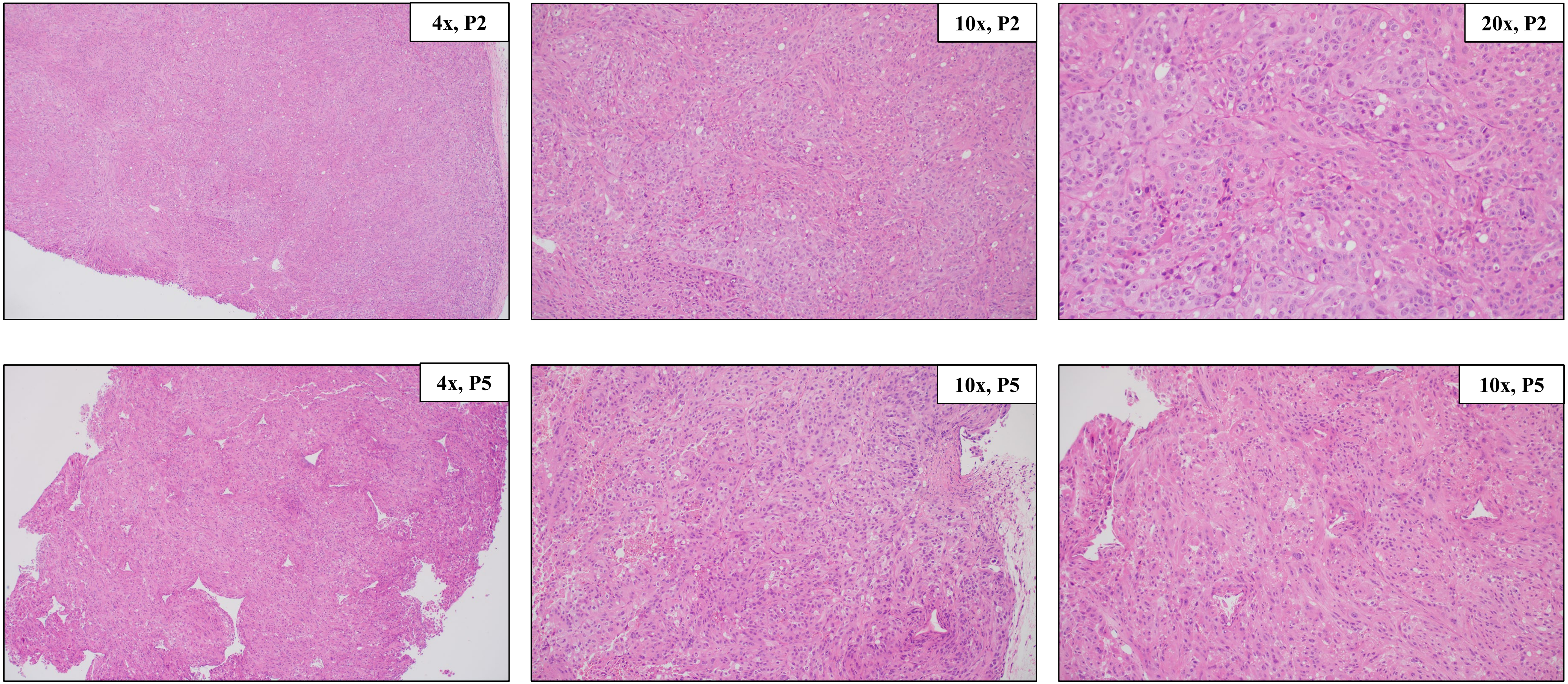

KRAS G12D, High Amp: CDK4, GAB2, MDM2

HCI-AM088, Acral Melanoma

Primary Tumor Location	PDX Tumor Location	Other Known Metastatic Locations
Right Foot Acral Skin	R Groin Lymph Node	Right Inguinal LN, R Humerus, T1-2 Vertebral Bodies, C1-C5 Epidural Soft Tissue, Brain

Clinical Tumor Histology

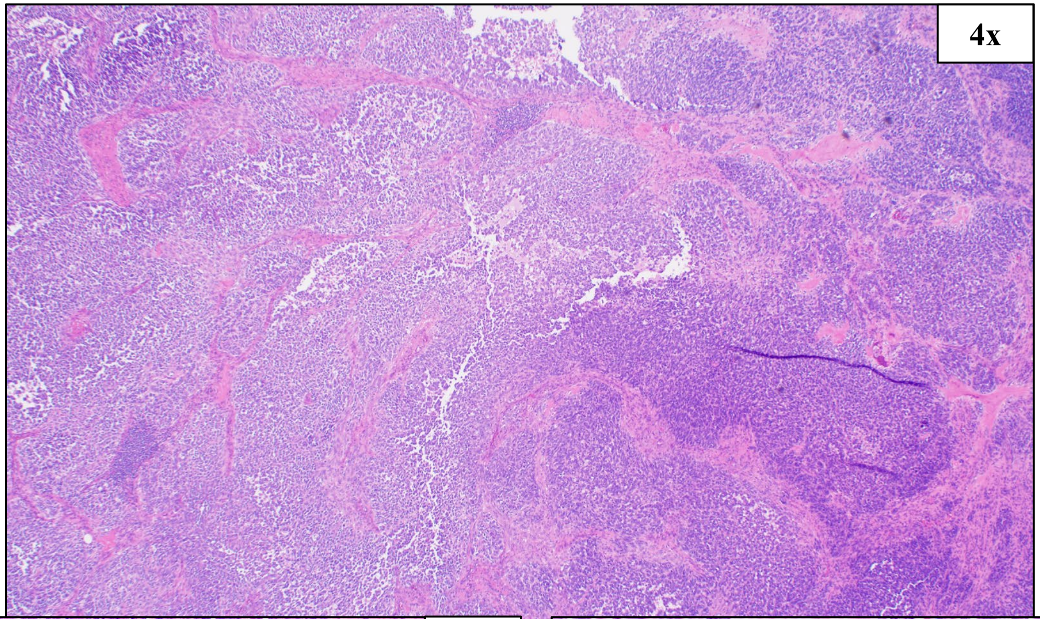

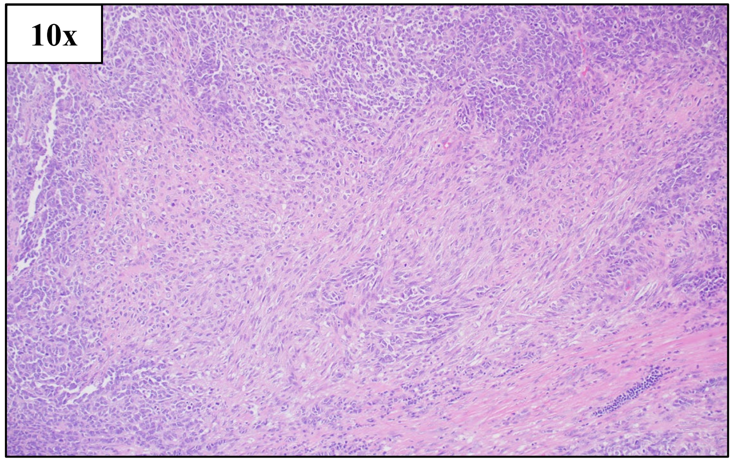

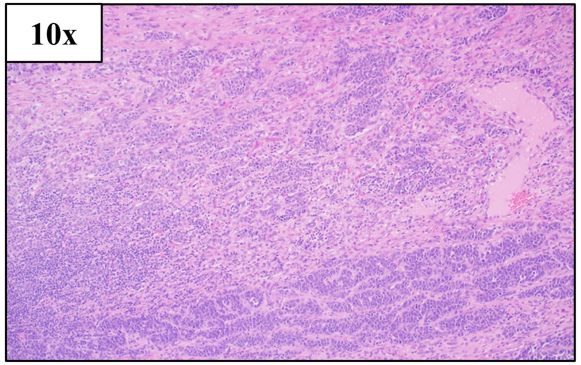

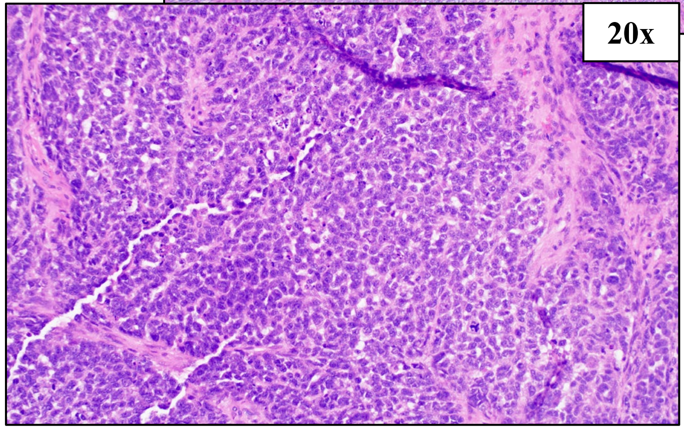

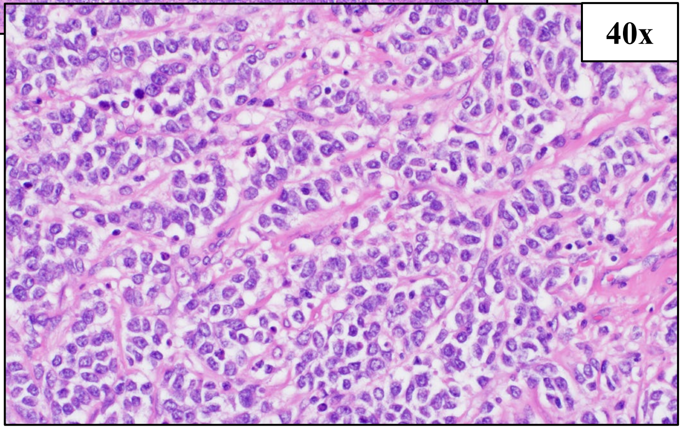

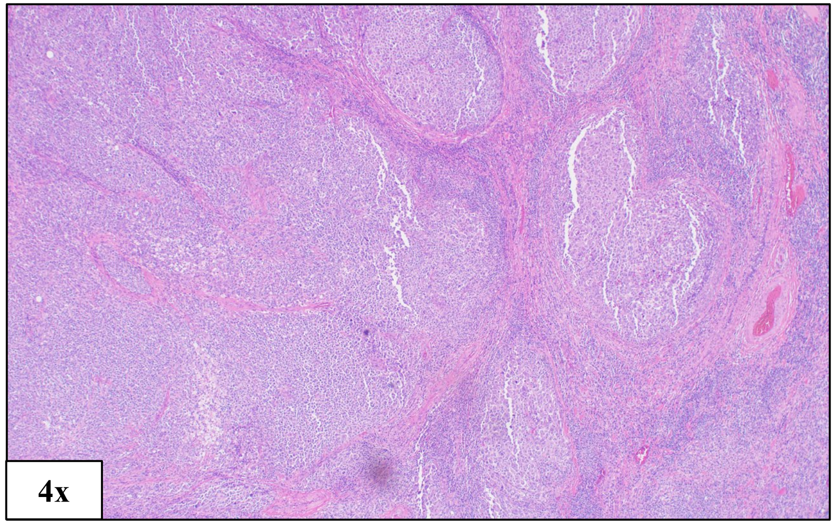

IHC Stains:

S100+/-, MelanA+

BRAF V600E

HCI-AM088, Acral Melanoma

SNV (%VAF)

BRAF c.1799T>A p.V600E (50%)
PTEN c.697C>T p.R233* (97%)
PIK3CA c.1035T>A p.N345K (Clinical NGS)

CNV

Amplification: HRAS, MC1R
Loss: PTEN, BRCA2, RB1

PDX Tumor Histology, Passage 2 and 3

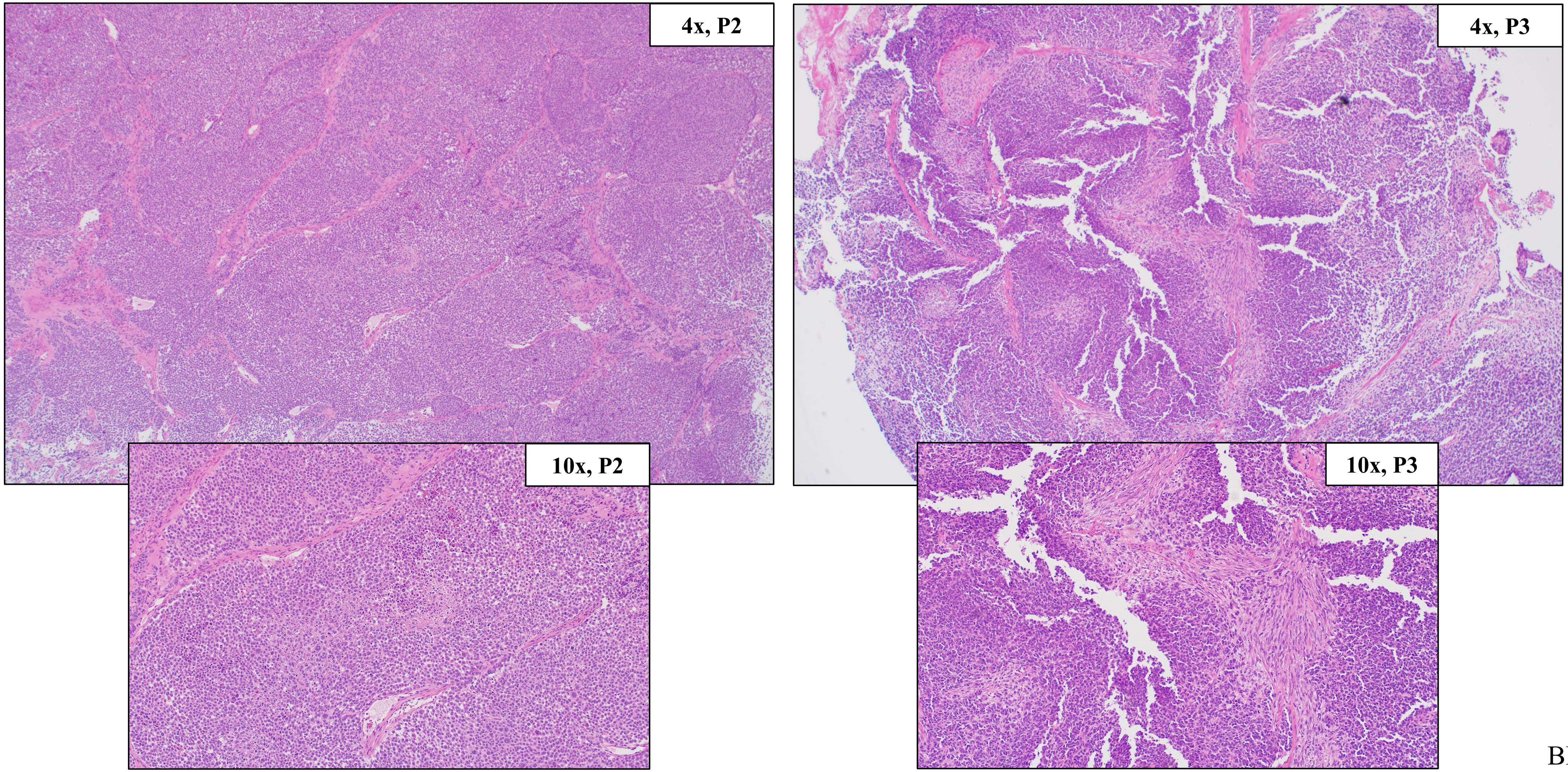

BRAF V600E

HCI-AM090, Acral Melanoma

Primary Tumor Location	PDX Tumor Location	Other Known Metastatic Locations
Left 4th Dorsal Toe and Nail Bed	Primary Tumor	Left Groin Lymph Node

Clinical Tumor Histology

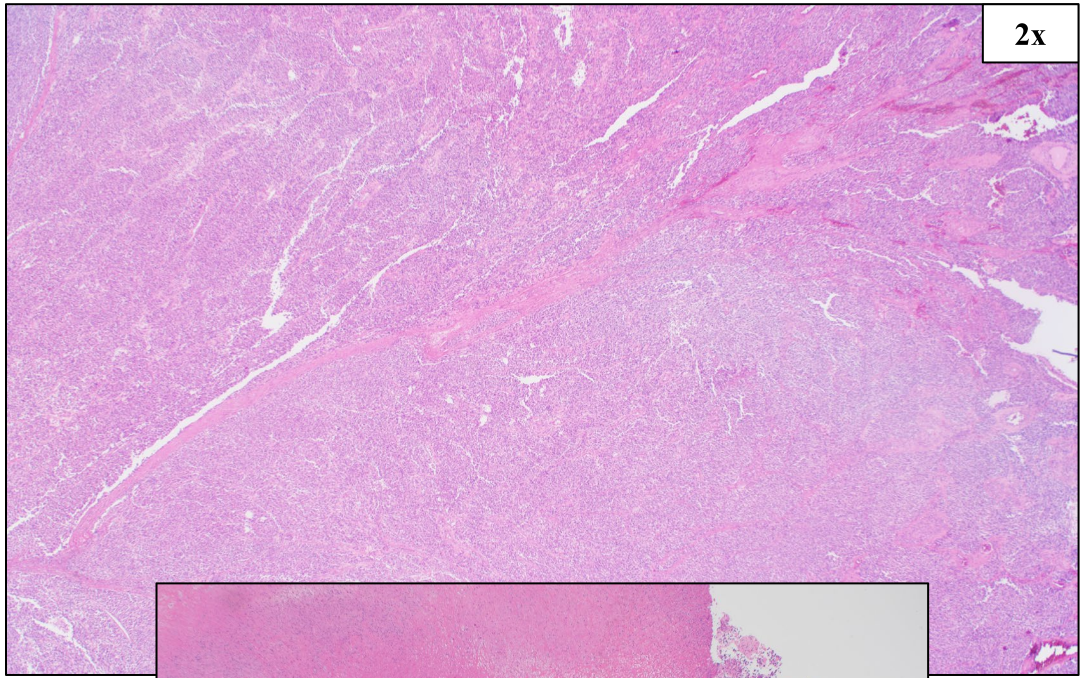

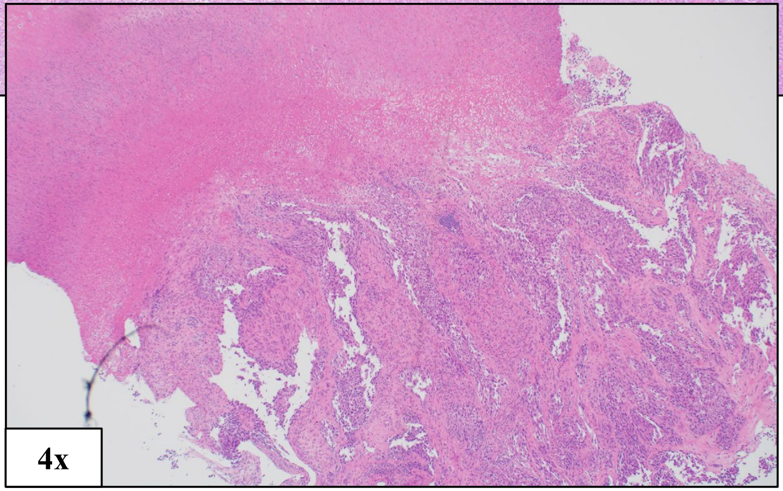

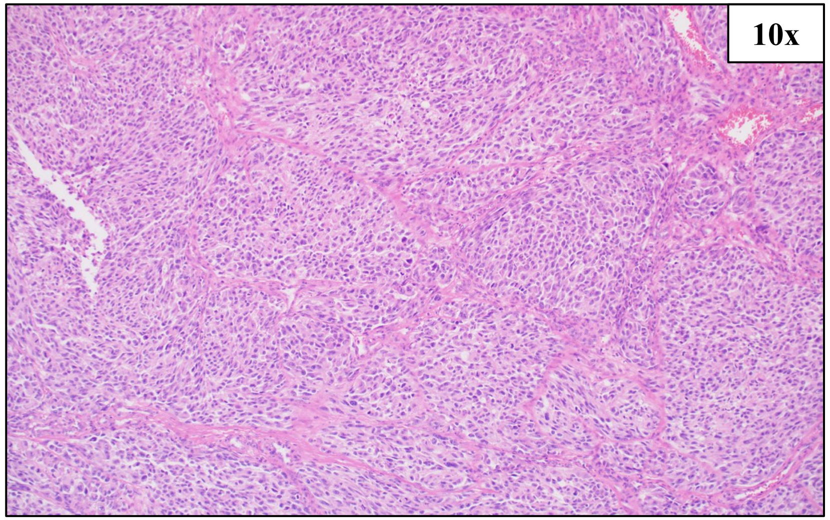

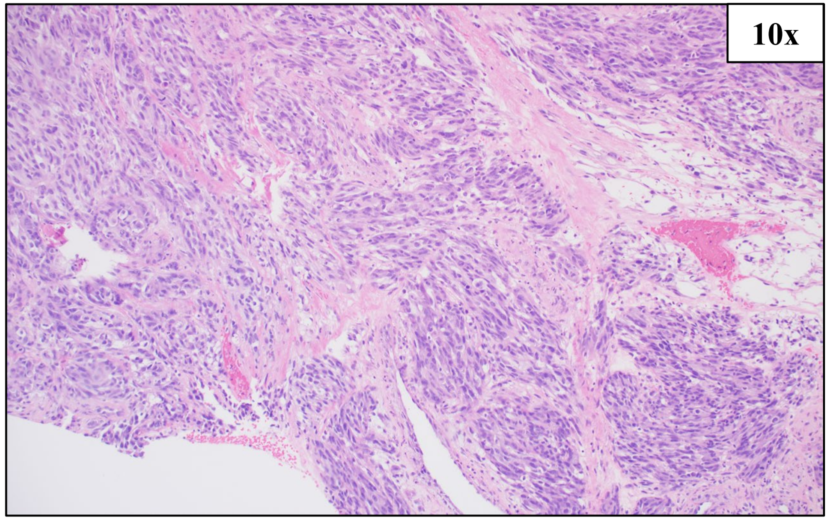

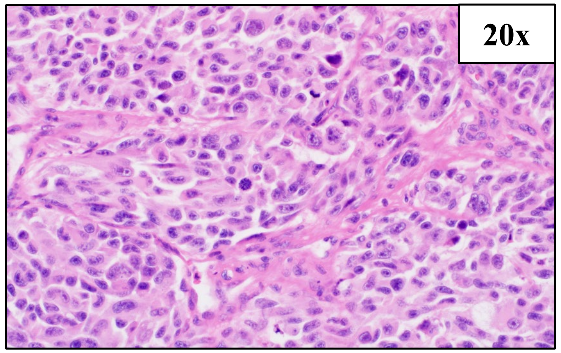

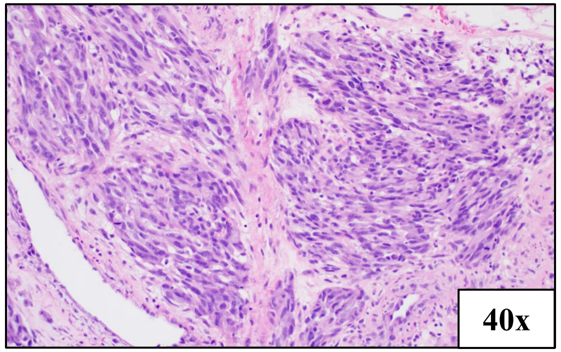

IHC Stains:

S100+, MART1+, SOX10+

High Amp: CRKL

HCI-AM090, Acral Melanoma

SNV (%VAF)

MAP2K1 c.362G>C, p.C121S (66%)

CNV

Amplification ≥8 copies: CRKL
Amplification: TERT, HRAS, CCND1, CDK4
Loss: ARID1B, CDKN2A, PTEN, BRCA2, RB1, SPRED1

PDX Tumor Histology, Passages 1 and 3

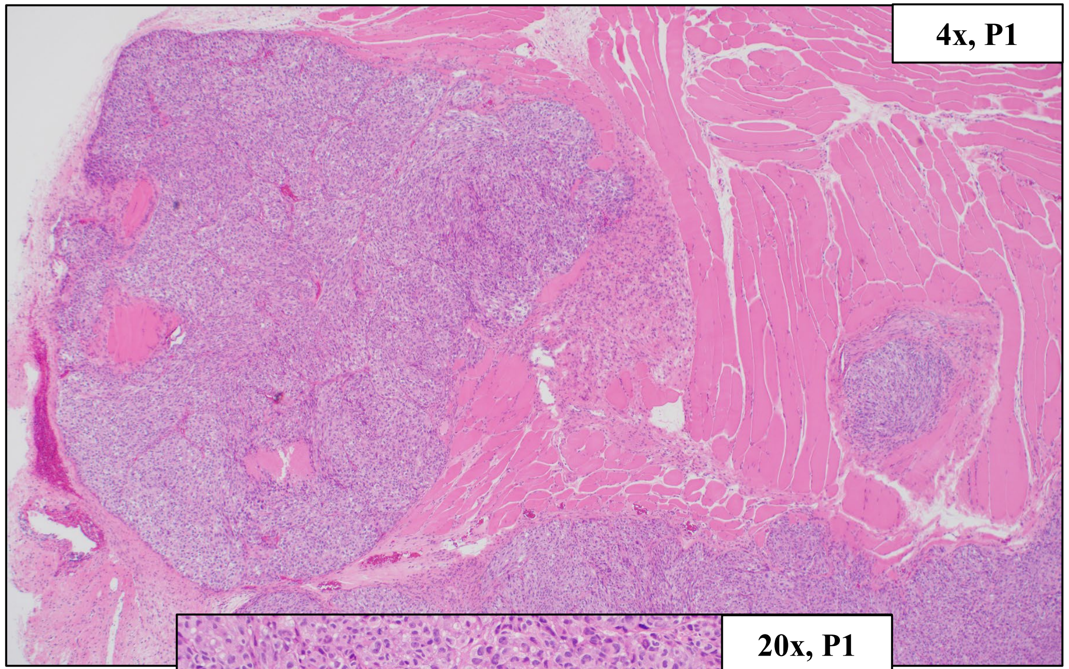

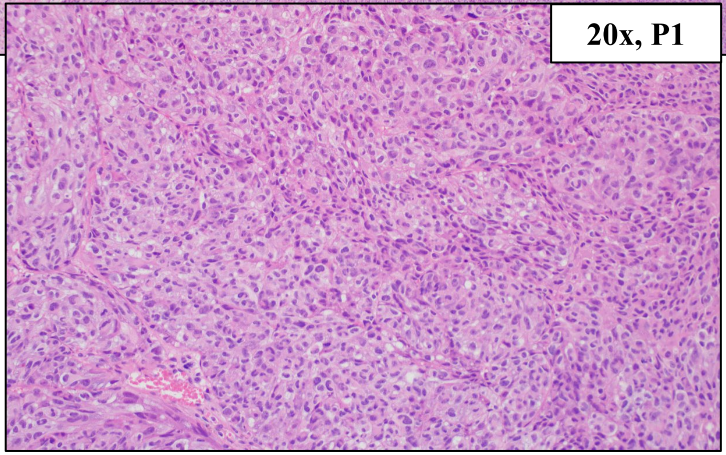

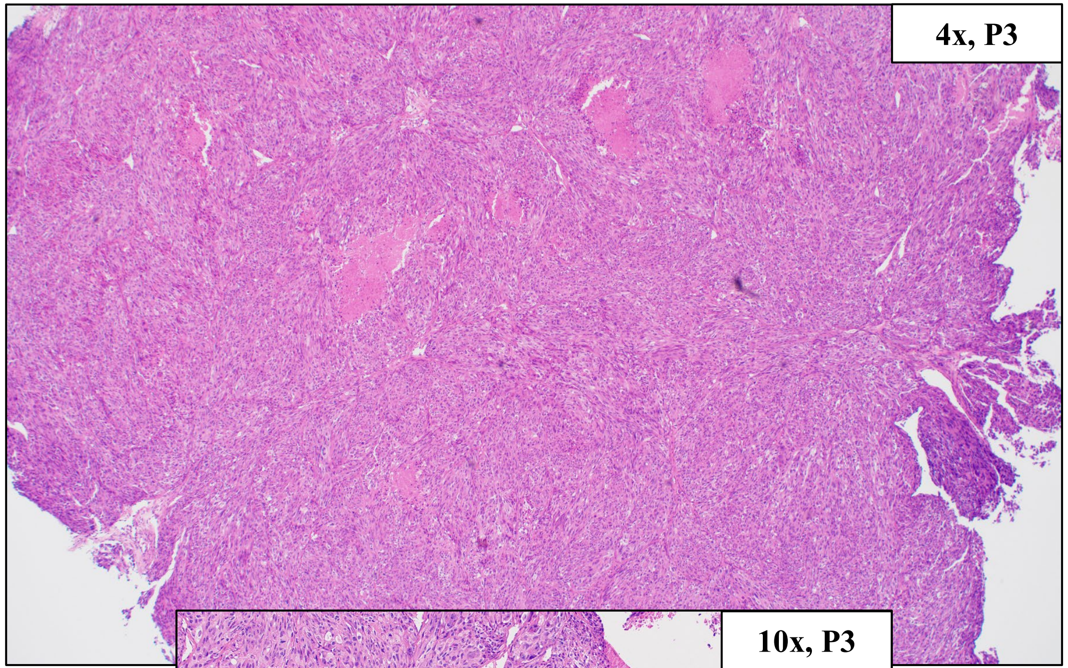

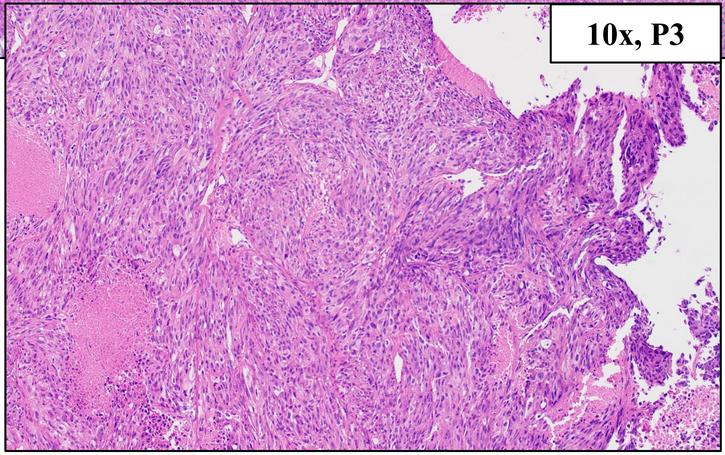

High Amp: CRKL

HCI-ASM091, Acral Melanoma

Primary Tumor Location

Left Thumbnail

PDX Tumor Location

Primary Tumor

Other Known Metastatic Locations

None

Clinical Tumor Histology

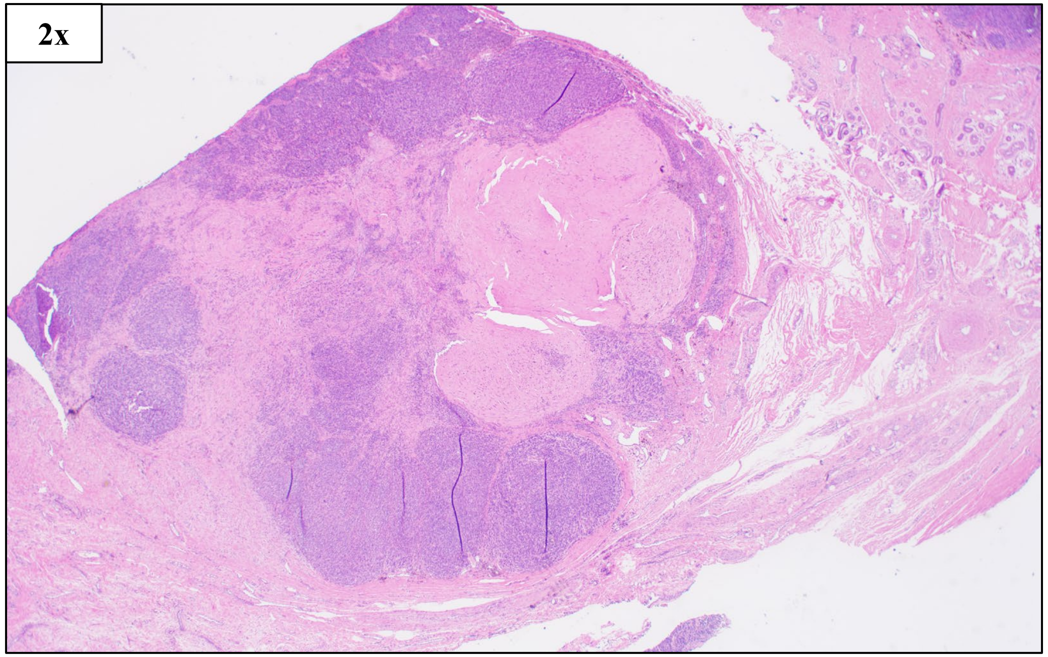

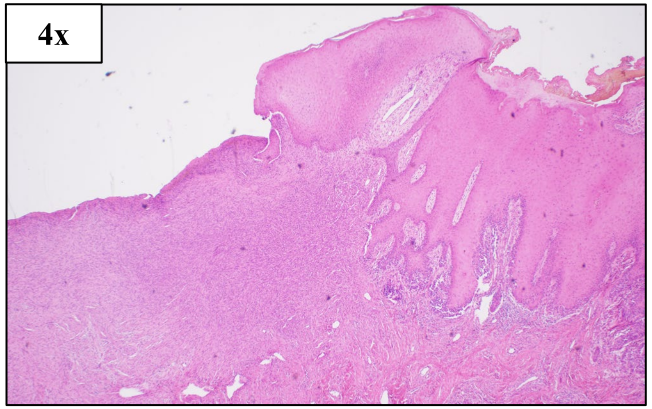

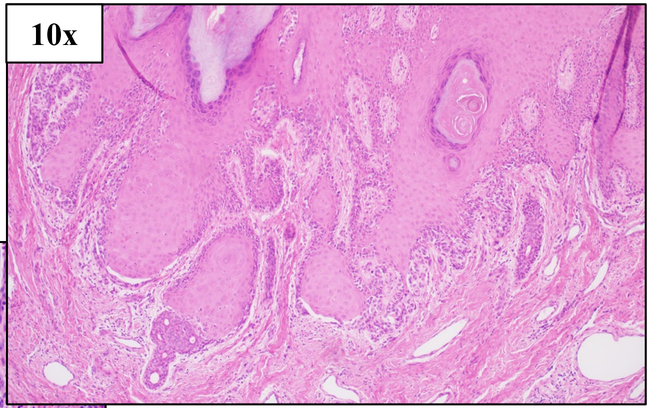

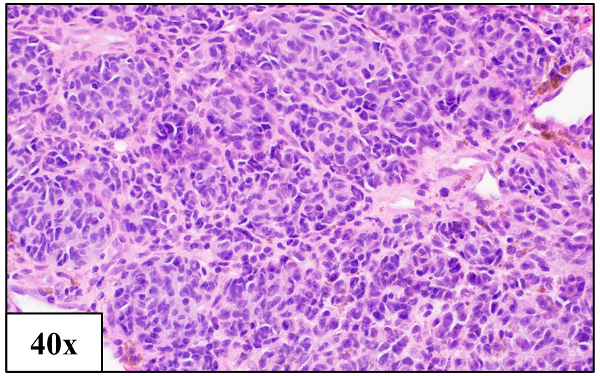

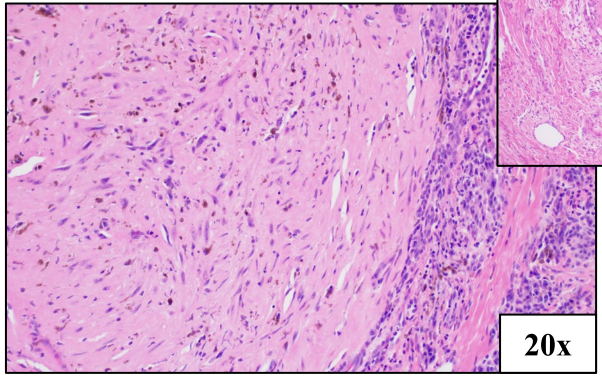

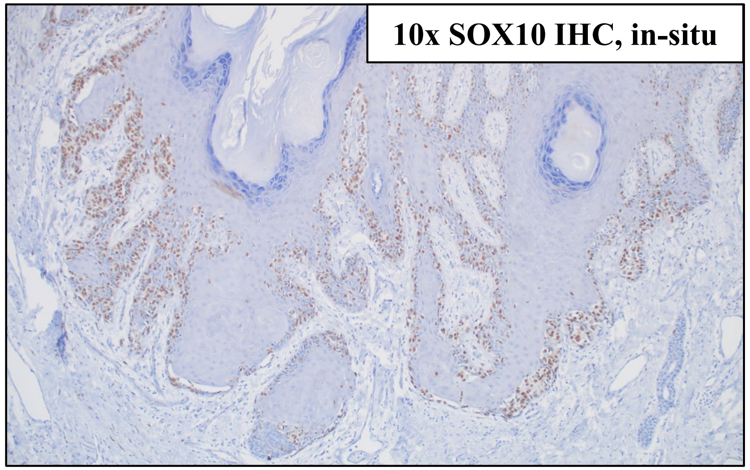

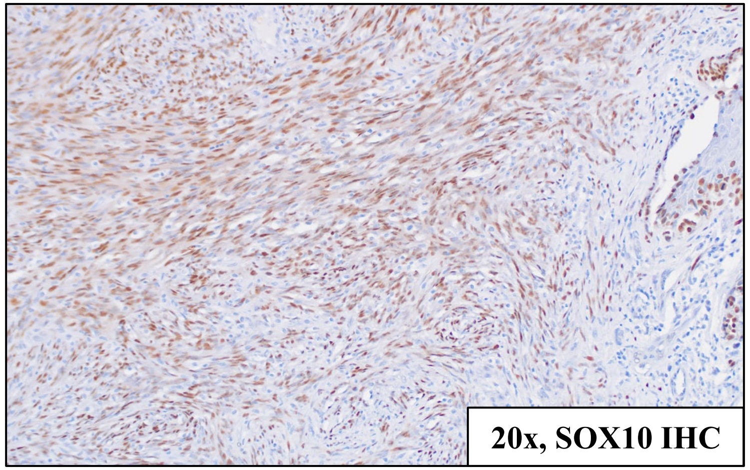

IHC Stains:

SOX10+

High Amp: GAB2

HCI-ASM091, Acral Melanoma

SNV (%VAF)

No Tier 1 or 2 mutations

CNV

Amplification ≥8 copies: GAB2
Amplification: HRAS, CCND1, BRCA1
Loss: ARID1B
Deep deletion: CDKN2A

PDX Tumor Histology, Passages 1 and 2

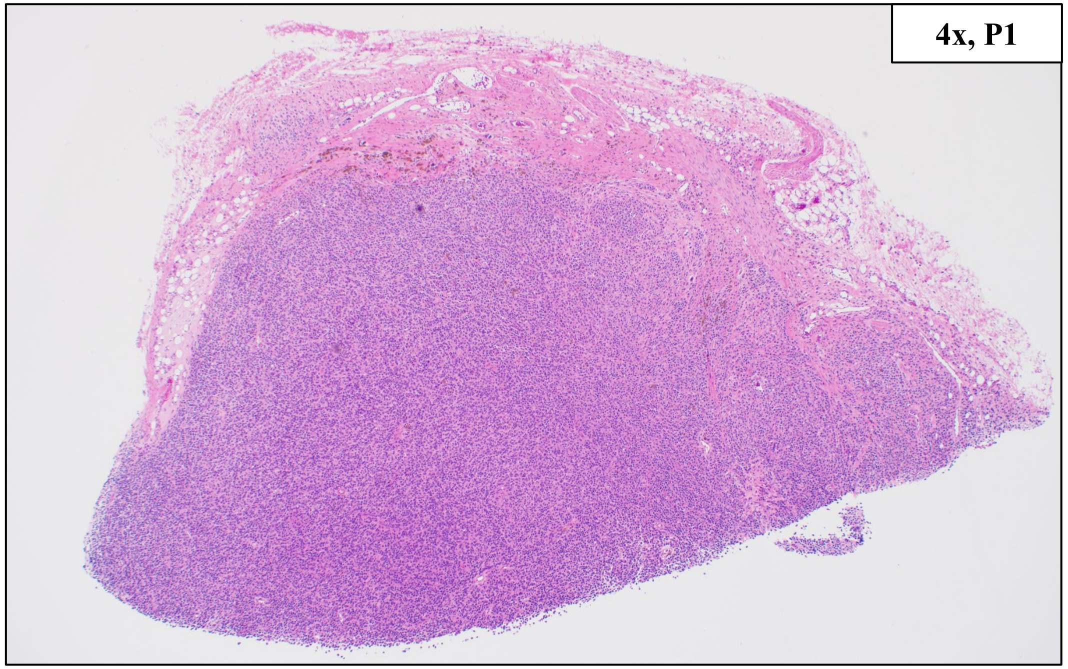

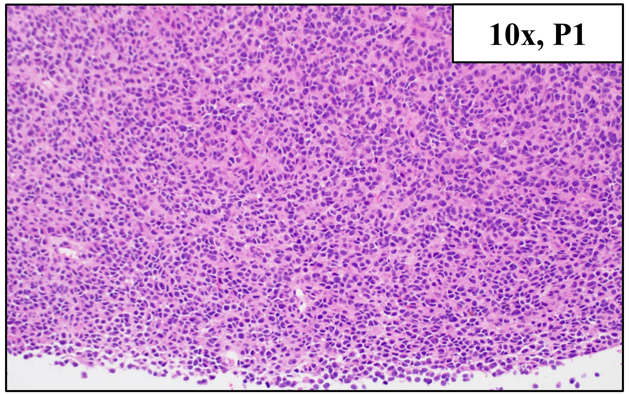

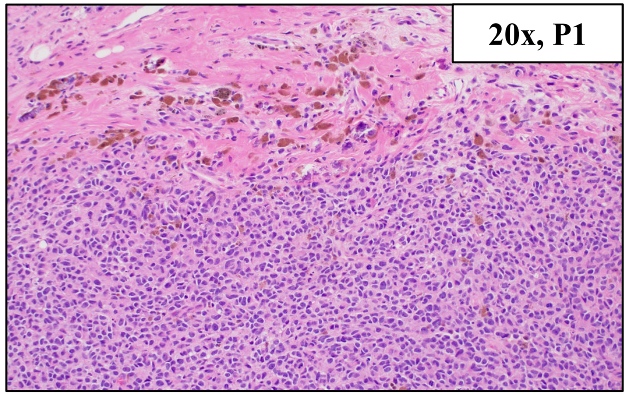

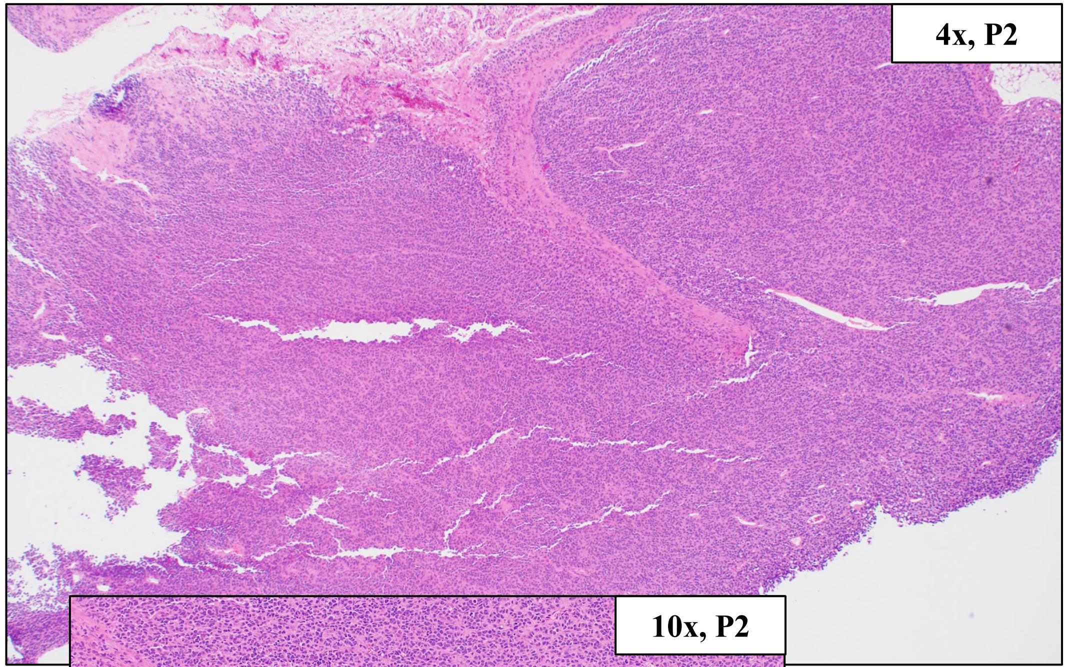

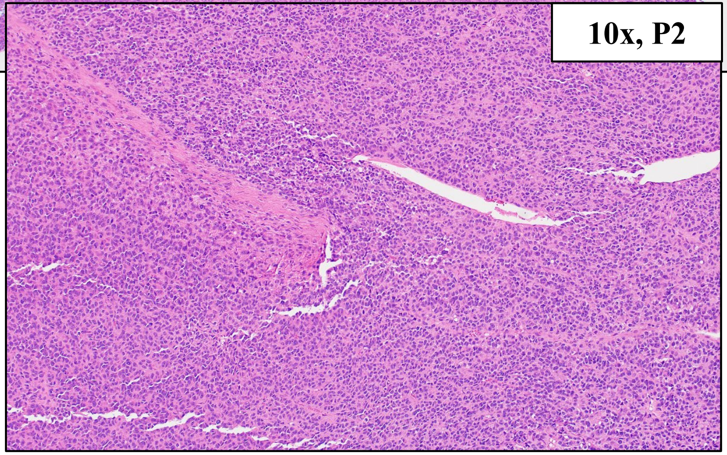

High Amp: GAB2

HCI-AM092, Acral Melanoma

Primary Tumor Location

Right Lateral Ankle at Acral Skin Junction

PDX Tumor Location

Primary Tumor

Other Known Metastatic Locations

Right Inguinal and Obturator Lymph Nodes

Clinical Tumor Histology

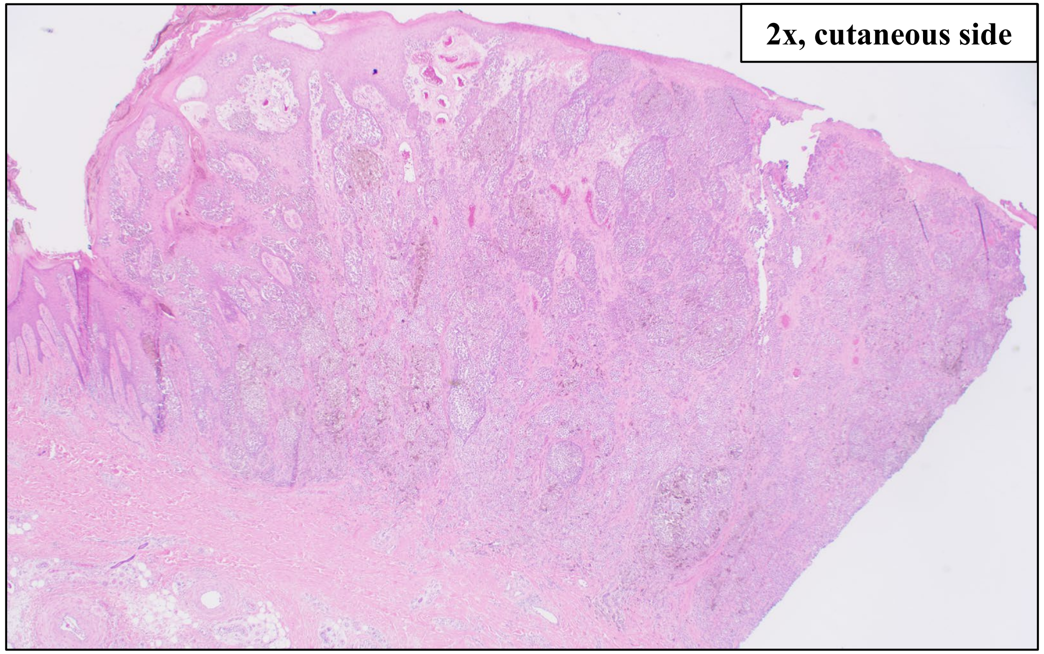

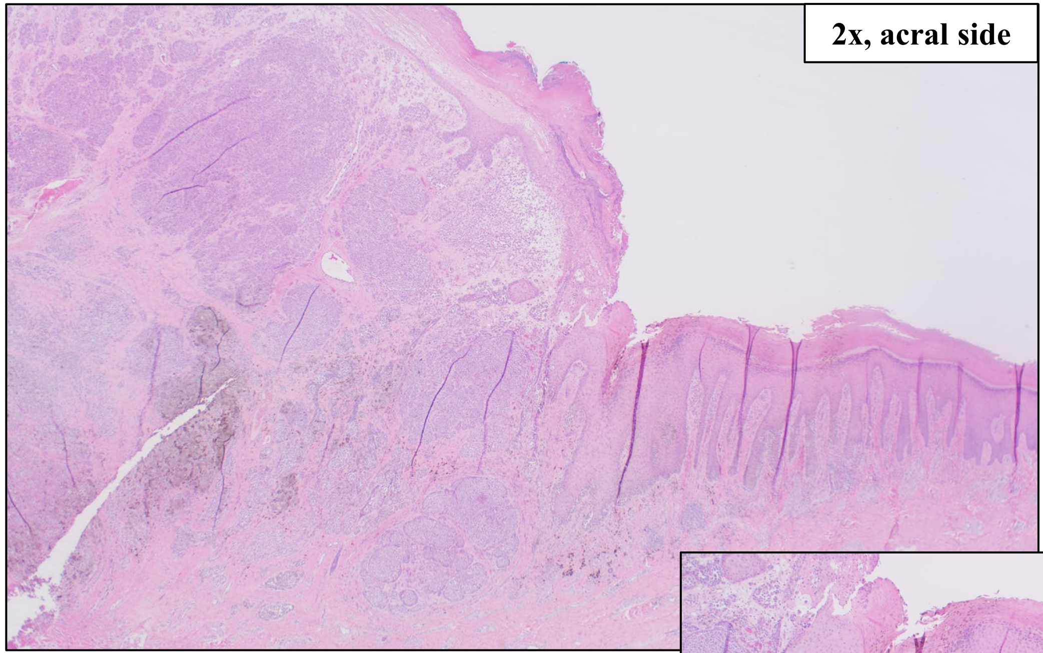

IHC Stains:
SOX10+, MelanA+

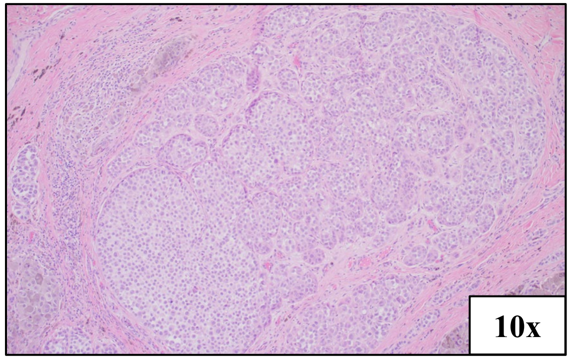

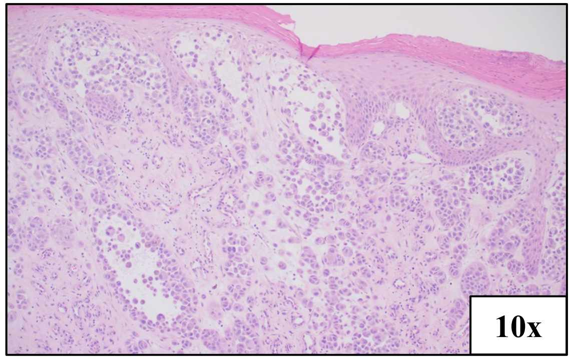

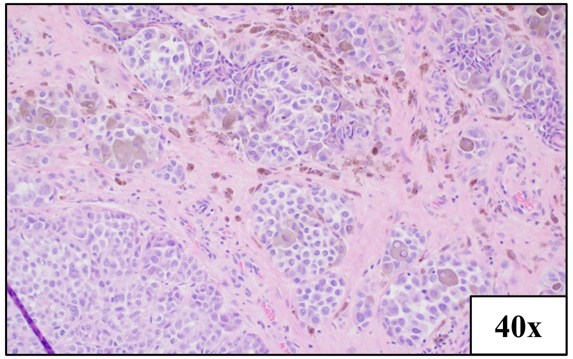

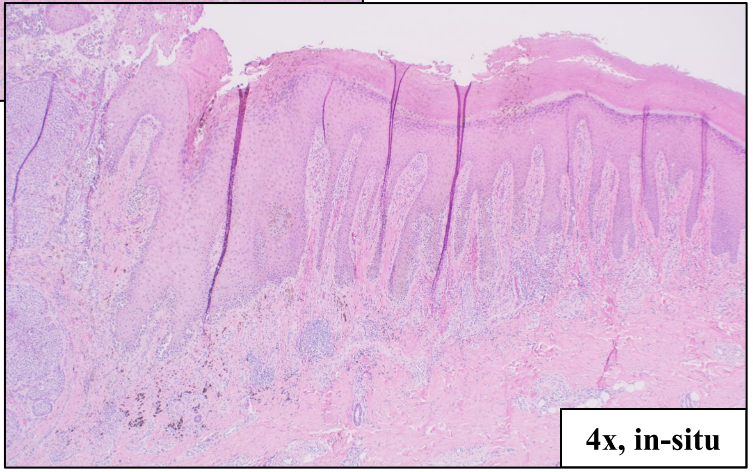

BRAF V600E

HCI-AM092, Acral Melanoma

SNV (%VAF)

BRAF c.1799T>A, p.V660E (65%)
CDKN2A c.301G>T, p.G101W (98%)

CNV

Loss: ARID1B, CDKN2A, HRAS, TP53
Deep deletion: PTEN

PDX Tumor Histology

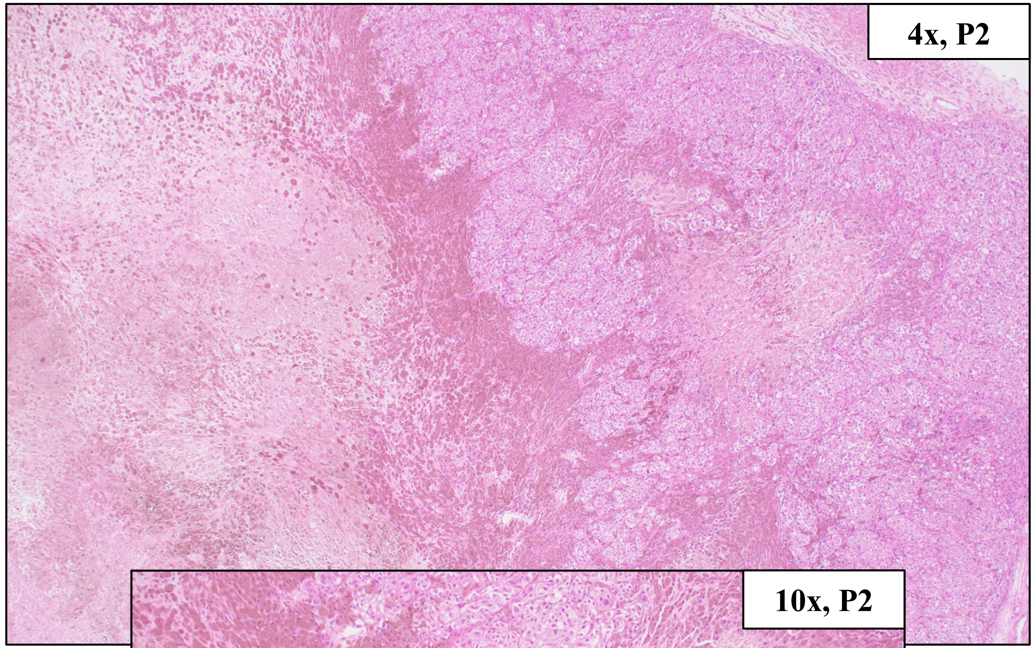

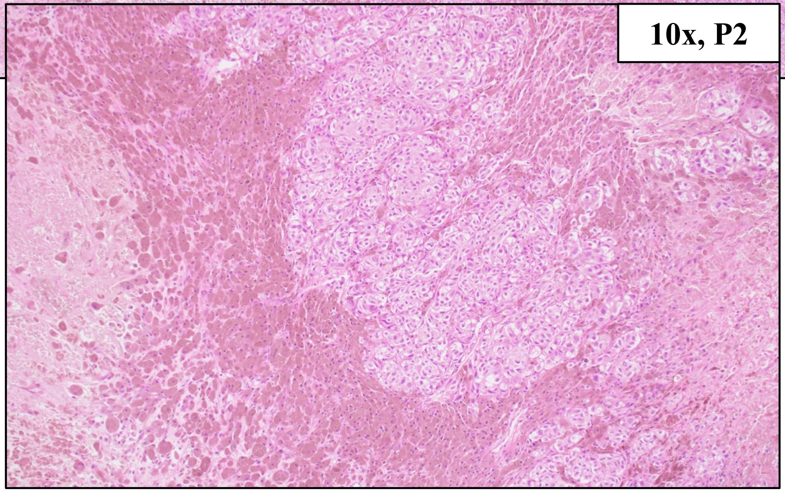

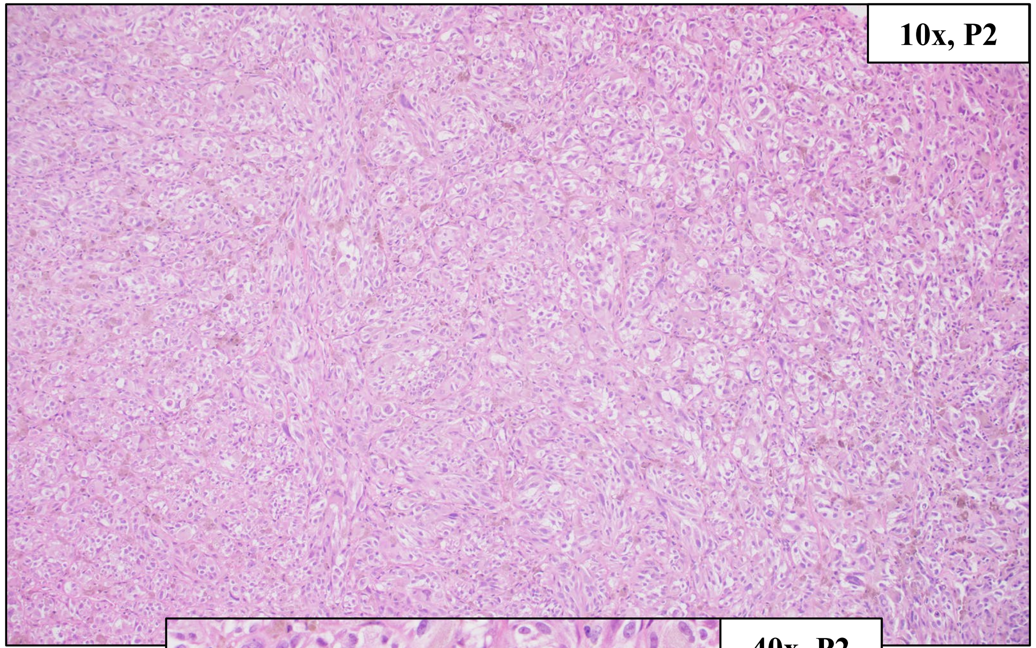

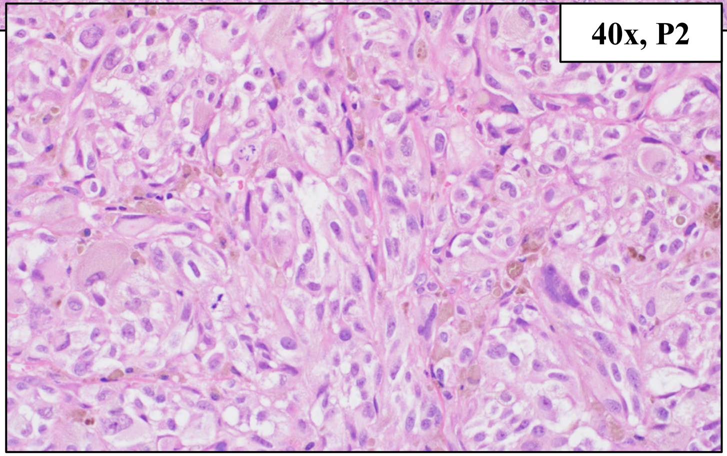

BRAF V600E

HCI-AM093, Acral Melanoma

<u>Primary Tumor Location</u>

Left 5th Toe, Lateral Volar Skin

<u>PDX Tumor Location</u>

Primary Tumor

<u>Other Known Metastatic Locations</u>

Left Medial Calf Skin and Lymph Nodes, Peritoneal Carcinomatosis

<u>Clinical Tumor Histology</u>

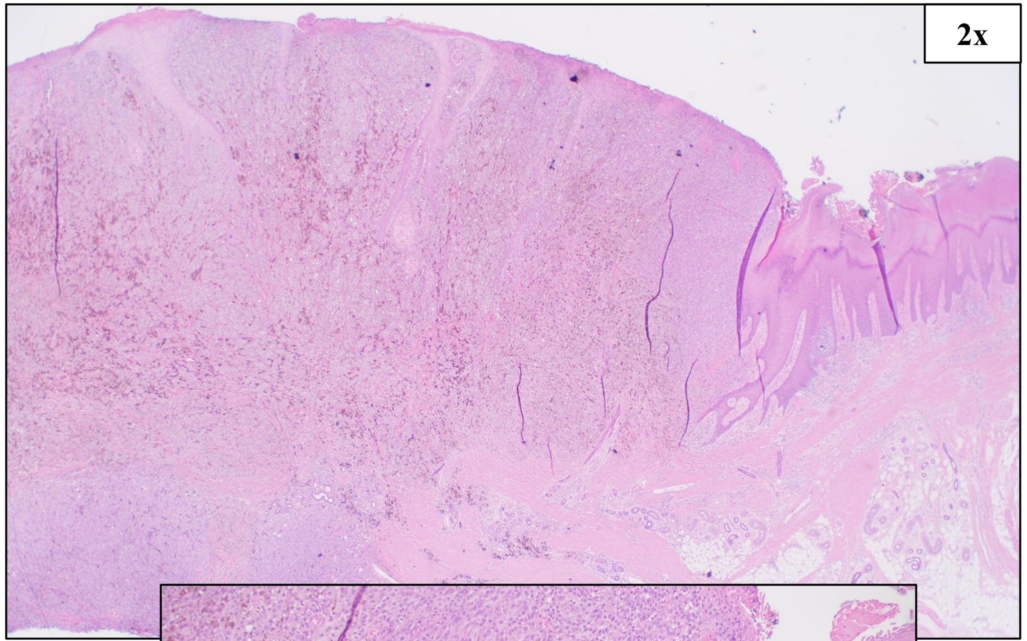

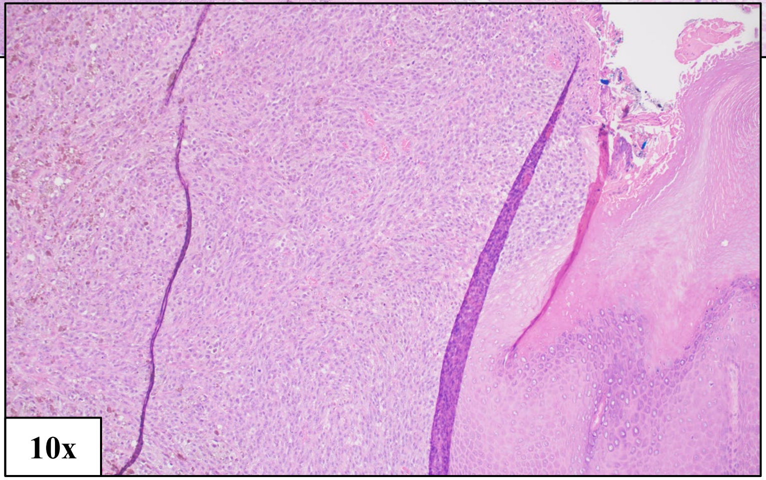

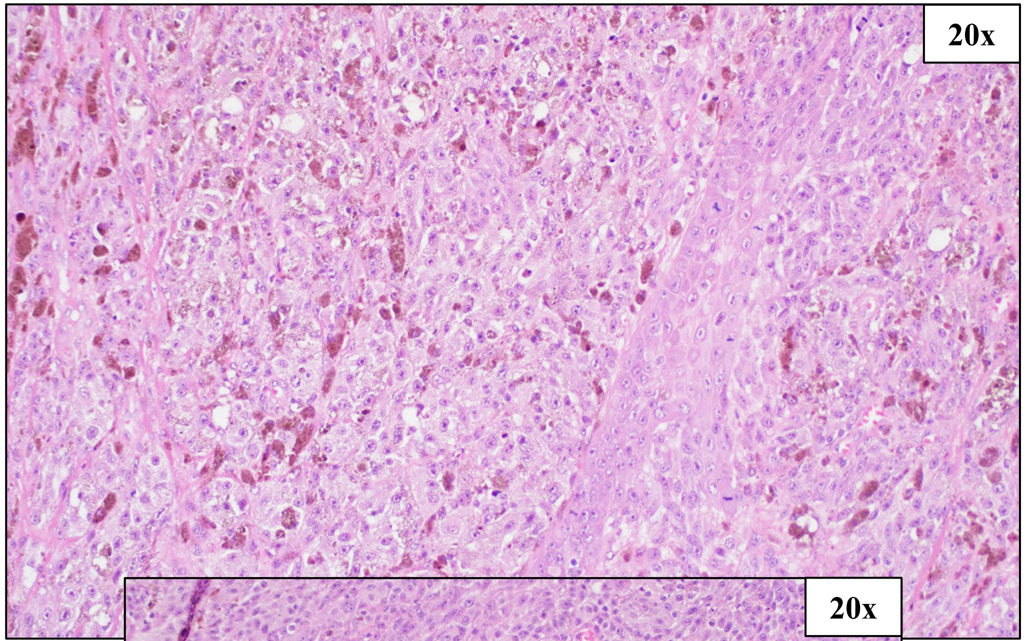

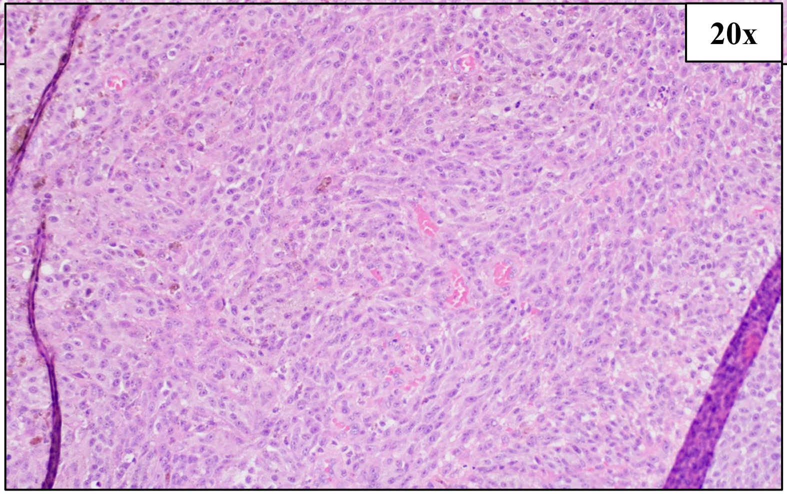

<u>IHC Stains:</u>

SOX10+

NRAS Q61R

HCI-AM093, Acral Melanoma

SNV (%VAF)

NRAS c.182A>G, p.Q61R (99%)

CNV

Amplification: TERT, CRKL
Loss: MET, CDKN2A, PTEN, GAB2

PDX Tumor Histology

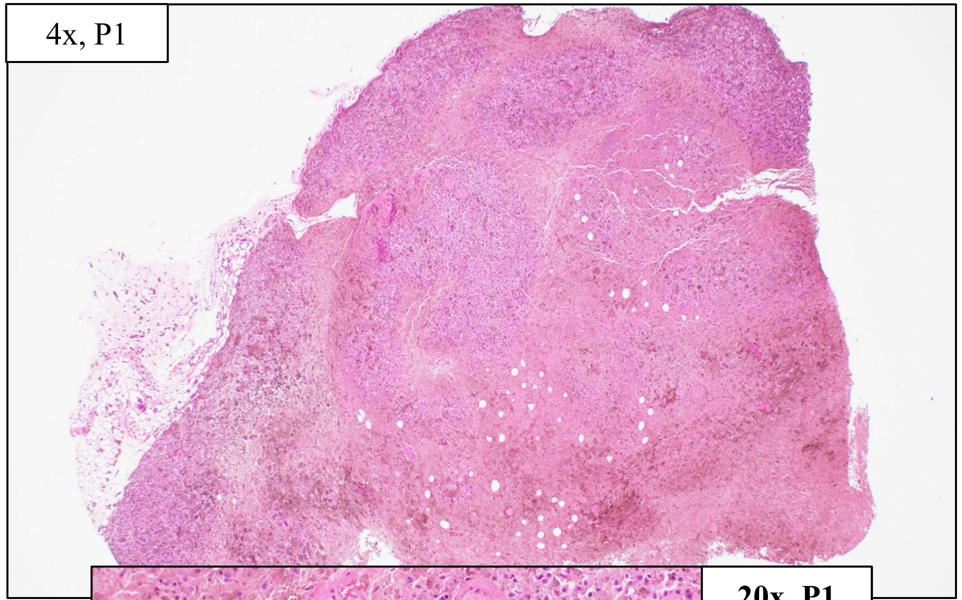

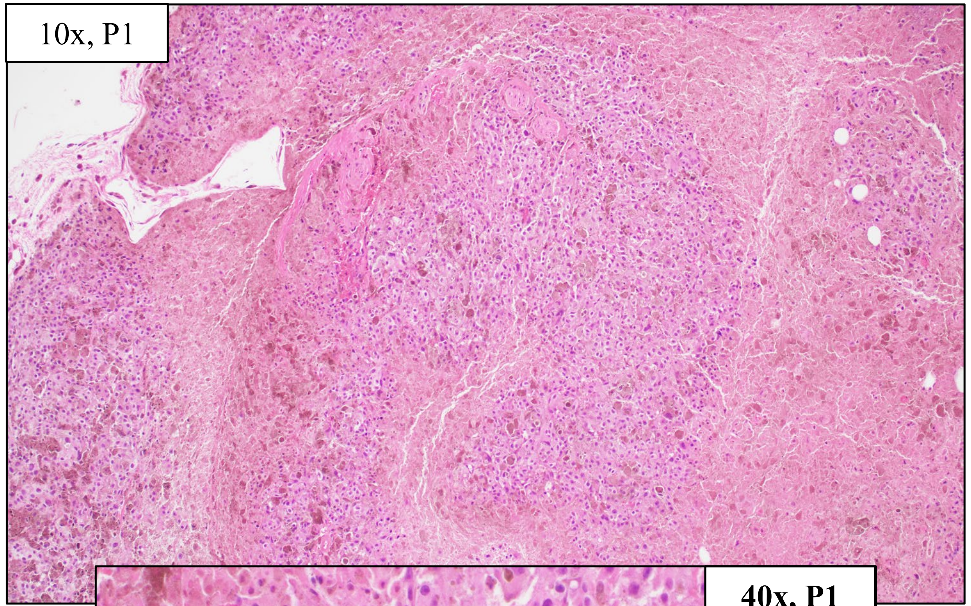

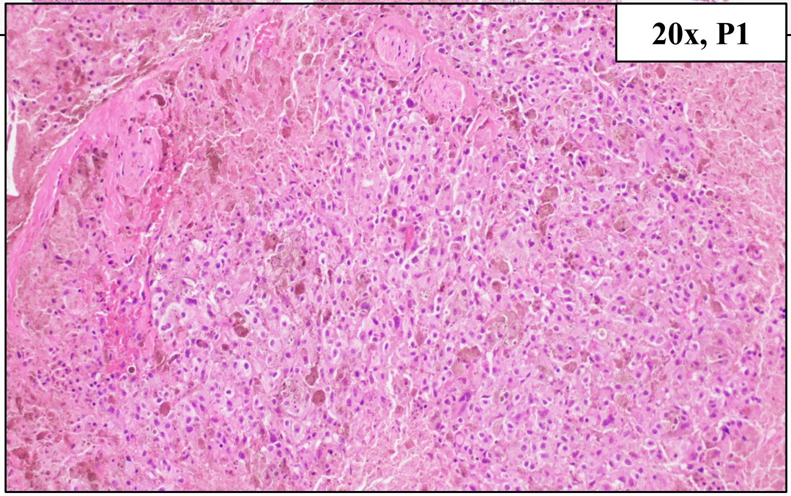

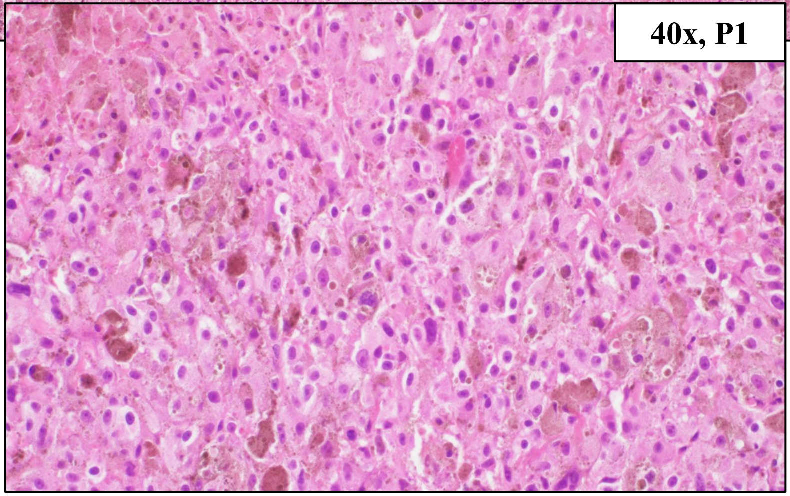

NRAS Q61R
