# Supplementary material for: Receptor tyrosine kinase inhibition leads to regression of acral melanoma by targeting the tumor microenvironment
Source: J Exp Clin Cancer Res. 2024 Dec 3;43:317. doi: 10.1186/s13046-024-03234-1 (PMC11613472; doi:10.1186/s13046-024-03234-1)
Supplement: Supplementary file 7 — Supplementary Material 7. Figure S7. Larger images of TfR1 and CD31 immunohistochemistry stains. H&E and TfR1 stains were imaged with a 10x objective, and CD31 was imaged with a 20x objective. Scale bars indicate length in microns. [file 13046_2024_3234_MOESM7_ESM.pdf]

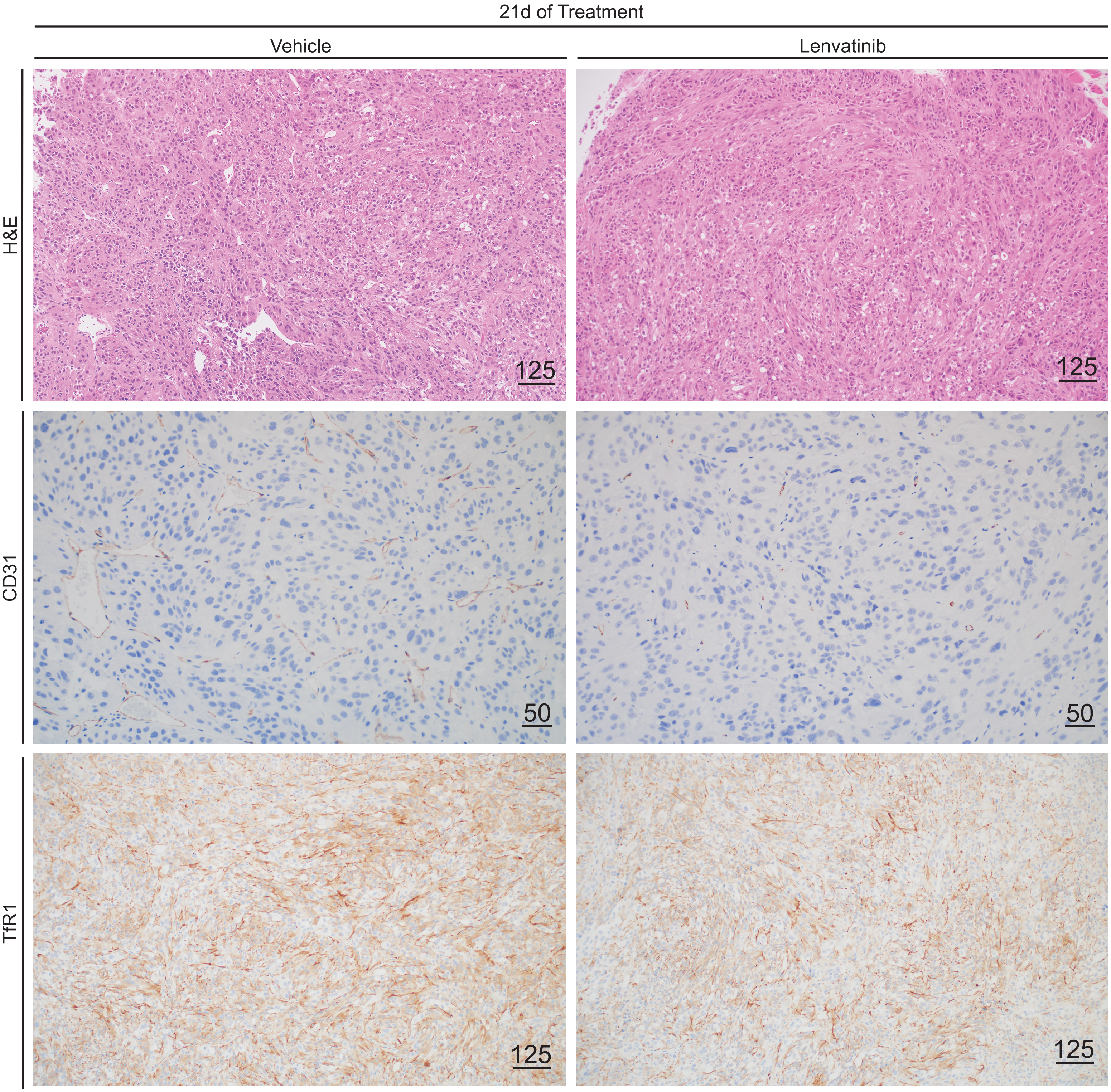
21d of Treatment
Vehicle
Lenvatinib
H&E
CD31
TfR1
125
125
50
50
125
125
